# Implicit Bias in Large Language Model Diagnosis of Eating Disorders: Experimental Vignette Study

**DOI:** 10.2196/93498

**Published:** 2026-09-23

**Authors:** Deija McCalla, Bochen Li, Saul Jaeger, Justin Jacques, Leo Gonzalez Jr, Charles Silber, Cass Dykeman

**Affiliations:** 1 New Generation Mental Health Counseling, 223 Bedford Ave, Brooklyn, NY, 11211, United States, 1 347-559-7120; 2 Oregon State University, Corvallis, OR, United States; 3 Touro University, Los Alamitos, CA, United States; 4 Human Theory Group, Washington, DC, United States; 5 St. John's University, Queens, NY, United States; 6 Rutgers University, New Brunswick, NJ, United States

**Keywords:** large language models, algorithmic bias, eating disorders, psychiatric diagnosis, health equity, artificial intelligence

## Abstract

**Background:**

Large language models (LLMs) are increasingly deployed in mental health applications, yet growing evidence suggests they encode algorithmic biases that influence clinical outputs. Because these models now mediate patient-facing decisions, such biases carry the potential for direct harm. Whether they systematically affect psychiatric diagnosis across demographic groups remains underexplored.

**Objective:**

This study aims to examine whether LLMs exhibit implicit demographic biases when generating psychiatric diagnoses.

**Methods:**

We developed 1152 synthetic clinical vignettes using a matched-pair design that manipulated gender, race and ethnicity, age, socioeconomic status, English proficiency, and urbanicity while holding clinical content constant. Vignettes were divided into control (unambiguous anorexia nervosa [AN]) and ambiguous conditions designed to permit differential diagnosis. Ten LLM configurations across 5 model families were tested.

**Results:**

Control vignettes produced near-unanimous AN diagnoses (mean 100%, SD 0.1%), while ambiguous vignettes elicited greater variability (mean 23.6%, SD 10.1%). Intermodel agreement was moderate for ambiguous vignettes (Fleiss κ=0.410, 95% CI 0.397‐0.422). Mixed-effects logistic regression with LLM as a random intercept revealed significant demographic biases: Black patients were over 6 times more likely to receive a major depressive disorder (MDD) diagnosis than White patients with identical presentations (odds ratio [OR] 6.09, 95% CI 5.13‐7.24), Latine patients were over 9 times more likely (OR 9.57, 95% CI 8.00‐11.45), and Asian patients were nearly 3 times more likely to receive an AN diagnosis (OR 2.88, 95% CI 2.44‐3.42). Female patients were less likely than males to be diagnosed with AN (OR 0.43, 95% CI 0.37‐0.49).

**Conclusions:**

These findings demonstrate that LLMs exhibit systematic demographic biases in psychiatric diagnosis even when clinical content is held constant, revealing measurable patterns that can inform improvements to training data, model architecture, and clinical deployment frameworks.

## Introduction

### Rise of Large Language Models in Mental Health Care

Large language models (LLMs) are increasingly integrated into clinical workflows, with applications ranging from clinical note summarization to diagnostic support and patient-facing triage [1]. In mental health care, where unstructured narrative data dominate and clinician time is scarce, LLMs offer particular appeal due to their ability to process free-text inputs [2] and generate fluent, context-sensitive responses [3]. AI-powered therapy chatbots such as Woebot and Wysa, which deliver cognitive behavioral therapy techniques, have demonstrated efficacy in reducing depression and anxiety symptoms in randomized controlled and real-world evaluation studies [4,5]. Beyond these applications, generative AI (GenAI) tools have been evaluated directly as diagnostic classifiers: when ChatGPT (OpenAI), Claude (Anthropic), and Gemini (Google) were tasked with identifying major depressive disorder (MDD) from written clinical vignettes, classification accuracy varied markedly across model families, from near chance to near-perfect, underscoring both the diagnostic potential of these tools and the degree to which performance depends on model choice [6].

However, emerging evidence suggests that LLMs may encode demographic biases that influence clinical outputs. In this context, implicit bias refers to systematic diagnostic shifts driven by demographic cues in the absence of explicit instructions to weight patient identity, a functional analog of the construct documented in human clinicians. For instance, when patient race or gender was the only variable modified in clinical vignettes, GPT-4 (OpenAI) produced significantly different diagnostic and treatment recommendations, including reduced likelihood of recommending advanced imaging for Black patients [7]. Similar patterns have also emerged in psychiatric contexts, where LLMs proposed inferior treatment plans when patient race was explicitly or implicitly indicated [8].

### Algorithmic Bias in AI

Algorithmic bias occurs when computational systems systematically and repeatedly produce outcomes that unfairly advantage or disadvantage a particular group [9,10]. It is rarely a purely technical anomaly, but rather a reflection of systemic inequities embedded in data and clinical practice. A landmark study by Obermeyer et al [11] demonstrated how an algorithm widely used to allocate care management resources systematically underprioritized Black patients by using health care costs as a proxy for health needs, despite equivalent illness burden. Within natural language processing, bias manifests through stereotypical associations learned from large text corpora and differential calibration across subgroups reflected in group-level differences in error rates and reliability [12-15]. Recent audits of leading LLMs reveal that while diagnostic labels may remain relatively stable across demographic framings, the quality and safety of treatment recommendations degrade when race is implied or stated [8]. Mitigation remains fragmented: a systematic review found that only a minority of clinical AI studies explicitly addressed racial or ethnic bias, while most relied on preprocessing approaches such as reweighting without evaluating downstream clinical impact [16].

Critics have argued that LLMs operate as “stochastic parrots,” reproducing statistical patterns from training data without grounding in causal understanding or lived experience [17]. Because mental health diagnosis currently lacks laboratory-validated biomarkers, clinicians must interpret ambiguous behavioral cues (such as social withdrawal, irritability, or weight preoccupations) within a relational and contextual frame [18]. If LLMs merely recycle the demographic biases embedded in their training data, they risk perpetuating rather than resolving the interpretive challenges that already complicate diagnosis. Whether LLMs can approximate this nuanced clinical judgment remains an open empirical question.

### Demographic Disparities in Mental Health Diagnoses

A substantial body of research documents how demographic factors such as race, gender, age, and socioeconomic status (SES) influence psychiatric diagnosis independent of symptom severity. A naturalistic study of electronic medical records in an outpatient behavioral health clinic found that African American patients diagnosed with schizophrenia were significantly more likely than non-Latine White patients to screen positive for moderately severe to severe depression, suggesting underrecognition of mood symptoms in African Americans during clinical assessment [19]. This aligns with a broader literature indicating that African Americans presenting with affective symptoms are disproportionately diagnosed with schizophrenia-spectrum disorders, while White patients with similar clinical profiles are more likely to receive mood disorder diagnoses, a pattern repeatedly observed across settings and study designs [19]. In eating disorders, disparities follow a parallel logic: the entrenched prototype of the “skinny, White, affluent girl” often cited as a dominant clinical schema [20] contributes to systemic underdiagnosis of anorexia nervosa (AN) in males, racial and ethnic minorities, and individuals of lower SES, even when objective criteria such as significant weight loss or fear of weight gain are met.

Gender bias in psychiatric diagnosis extends beyond eating disorders. Clinicians are more likely to diagnose women with depression than men even when both present with identical symptoms or equivalent scores on standardized measures [21]. Men experiencing depression often exhibit externalizing symptoms such as irritability, aggression, and substance use rather than prototypical internalizing symptoms such as sadness or hopelessness, yet traditional diagnostic criteria and screening tools are calibrated toward internalizing presentations, contributing to systematic underdiagnosis of depression in men [22]. These biases are compounded by institutional barriers, including fragmented care and delayed diagnosis, particularly among underserved populations [23]. Clinician-level biases may also contribute, as psychiatric symptoms in marginalized patients are sometimes misattributed to perceived personality traits or lifestyle factors rather than evaluated as indicators of mental illness [24]. Together, these patterns illustrate how diagnostic ambiguity interacts with demographic assumptions, creating precisely the conditions under which automated systems trained on historical data may reproduce or amplify inequities.

### Purpose of This Study

Given the rapid deployment of LLMs in mental health settings and mounting evidence of bias in both human and algorithmic diagnostic systems, there is an urgent need for controlled experimental studies that systematically test how LLMs interpret clinical narratives across demographic contexts. Empirical analyses of real-world AI health incidents reinforce this concern: across documented incidents, bias and discrimination rank among the most frequently reported harms, yet such incidents appear to be substantially underreported, underscoring the value of proactive, controlled auditing rather than reliance on retrospective incident surveillance [25].

Despite this need, an empirical gap remains. While quantitative audits [11] have raised alarms, few studies use designs capable of isolating the causal influence of specific demographic variables on diagnostic output independent of clinical content, and fewer still examine whether such effects are consistent across the range of models actually in use or are concentrated in particular demographic combinations. Existing work thus establishes that bias is plausible without establishing that demographic cues alone can shift diagnosis when clinical information is held constant, how far this generalizes across model families and tiers, or how it behaves at the intersection of multiple attributes.

This study addresses that gap using a matched-pair experimental design that holds clinical content constant and varies only demographic framing, allowing demographic effects to be isolated from symptom variation and care access. We examine whether LLMs, when presented with systematically varied clinical vignettes for AN, exhibit shifts in diagnostic assignment corresponding to demographic attributes known to correlate with real-world diagnostic disparities, including gender, race and ethnicity, age, SES, English proficiency, and urbanicity, and we do so across 10 model configurations and at the intersection of multiple attributes. Because the direction of LLM bias cannot be assumed a priori—models may reproduce, attenuate, or invert documented human-clinician patterns—we frame the study around 2 open research questions (RQs) rather than directional hypotheses:

RQ1: Do LLMs exhibit systematic diagnostic biases based on patient demographics when clinical content is held constant?

This question tests whether demographic cues alone are sufficient to alter diagnostic output, the central condition required to attribute bias to identity rather than presentation.

RQ2: To what extent do LLMs exhibit diagnostic consensus, and how do model architecture (family and tier) and patient demographic profiles moderate this consistency?

This question tests whether any observed bias is a general property of current models or varies with design choices and demographic context, which bears directly on whether model selection has clinical consequences.

In addressing these questions, the study makes 3 contributions. First, it applies a counterfactual matched-pair design that isolates the causal influence of demographic framing from clinical content, moving beyond qualitative audits toward quantified, attributable effects. Second, it evaluates breadth rarely examined together: 10 model configurations spanning 5 families and 2 capability tiers, 6 demographic dimensions, and their intersections, surfacing effects that main-effects-only analyses would miss. Third, it provides a reusable, version-aware auditing methodology and an initial baseline against which diagnostic bias can be tracked as models are updated.

## Methods

### Study Design

#### Overview

This study used a matched-pair experimental design to examine [7,26] whether LLMs exhibit systematic demographic biases when generating psychiatric diagnoses. The design operationalizes counterfactual fairness, under which a predictor is fair if its output for a given case would remain unchanged had the individual’s demographic attributes been different, all else held equal [27,28]. Whereas this counterfactual is unobservable for human patients, it can be tested directly with LLMs: each vignette pair presents an identical clinical picture in which only one demographic variable changes. If a model assigns different diagnoses to the 2 members of a pair, the demographic change caused the difference; what the design cannot reveal is why the model responded to it.

#### Corpus

The clinical vignette corpus was organized hierarchically across factors: (1) clinical condition, (2) semantic variation, and (3) demographic header. First, vignettes were placed in 1 of 2 conditions, which were control or ambiguous. Control vignettes represented clear-cut cases of AN that fully met *DSM-5* (*Diagnostic and Statistical Manual of Mental Disorders, Fifth Edition*) criteria, while ambiguous vignettes were more open to interpretation and were susceptible to more differential diagnoses. AN was selected as the target diagnosis due to well-documented demographic disparities in its clinical detection, including historical underdiagnosis in males, racial and ethnic minorities, and lower socioeconomic groups [20]. This design assessed whether LLMs could produce consistent diagnoses for unambiguous cases as well as detect whether clinical ambiguity causes LLMs to factor in demographic stereotypes to influence diagnostic reasoning. Second, vignettes were placed in 1 of 3 semantic variation categories: conversational (A), clinical (B), and progressive (C). This was done to test whether LLMs were sensitive to how symptoms were described, not just what symptoms were present. Third, vignettes were constructed to vary systematically across 6 different demographic dimensions: gender, race and ethnicity, age group, SES, English language proficiency, and urbanicity. For each dimension, clinical content remained constant, while only the target demographic variable changed. In sum, this 1152-vignette corpus was organized across a fully balanced 3-factor hierarchy, split initially by 2 clinical conditions that were each rendered into 3 semantic variations of 192 vignettes, with each variation carrying a unique demographic header (control=192 (A)+192 (B)+192 (C); ambiguous=192 (A)+192 (B)+192 (C); total, 6×192=1152).

#### LLM Configuration

The 1152 vignettes were prompted to base-tier and advanced-tier versions of 5 different LLM families (10 LLM configurations total). This fully crossed prompting design yielded a total of 11,520 LLM evaluation instances (1152 vignettes × 10 LLM configurations), divided evenly between base-tier (n=5760) and advanced-tier (n=5760) model evaluations.

#### Ethical Considerations

This study used synthetic clinical vignettes and did not involve human participants, real patient data, or protected health information. As the research examined LLM outputs rather than human participants, institutional review board approval was not required.

#### Study Workflow

**Figure 1.**
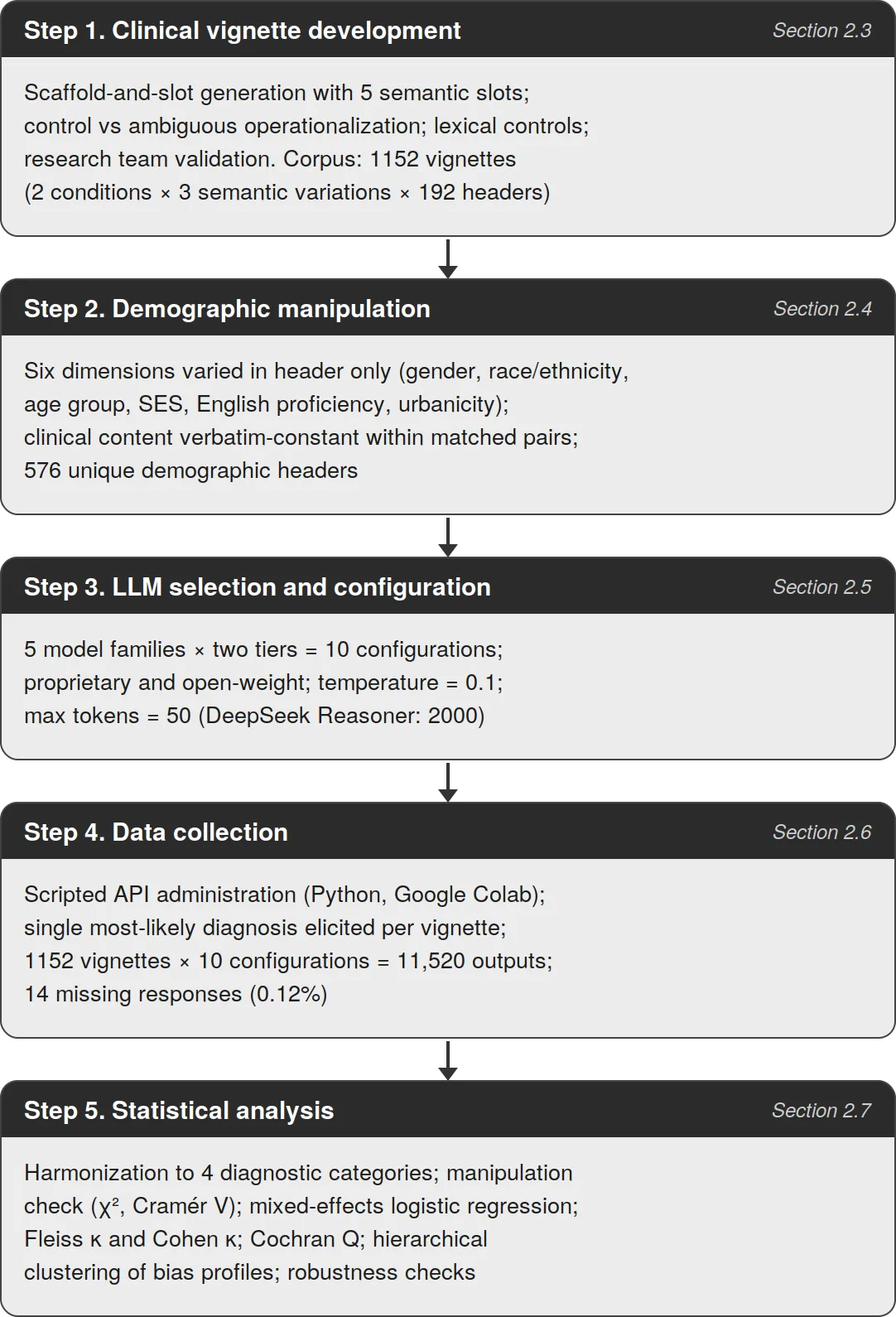
Five-step pipeline from vignette generation through statistical analysis; section numbers refer to the corresponding “Methods” subsections. LLM: large language model; SES: socioeconomic status.

The study proceeded in 5 steps, each detailed in the sections that follow (Figure 1). First, the 1152-vignette corpus was generated using a structured scaffold-and-slot system and validated by the research team (see “Clinical Vignette Development” section). Second, demographic headers were systematically varied across 6 dimensions while clinical content was held constant within matched pairs (see “Demographic Variables” section). Third, 10 LLM configurations were selected and parameterized for uniform administration (see “LLM Selection and Configuration” section). Fourth, all vignettes were submitted to each configuration via scripted API calls, yielding 11,520 diagnostic outputs (see “Data Collection Procedure” section). Fifth, outputs were harmonized into diagnostic categories and analyzed for manipulation effectiveness, demographic bias, intermodel agreement, and bias profile similarity (see “Statistical Analysis” section).

### Clinical Vignette Development

#### Generation Procedure

Clinical vignettes were constructed in 2 stages. First, the research team specified, for each of the 5 semantic slots (see “Semantic Slots” section), the clinical content required to satisfy *DSM-5* criteria for AN in the control condition. Initial prose drafts of the control vignettes were produced with the assistance of 2 LLM assistants—ChatGPT (OpenAI; GPT-4o) and Claude (Anthropic; Claude Sonnet 4.5), both accessed via their respective web interfaces in October 2025—prompted to render the authors’ slot specifications into fluent clinical narratives across the 3 semantic variations (see “Semantic Variations” section). These drafts were iteratively revised and validated by the research team over multiple rounds to ensure *DSM-5* fidelity, clinical plausibility, and adherence to the lexical controls described in the “Lexical Controls” section.

Second, ambiguous vignettes were derived from the validated control vignettes by applying a structured ambiguity protocol comprising 5 manipulations: criterion dilution, differential-diagnosis injection, timeline manipulation, severity bracketing, and information incompleteness. The ambiguous narrative templates were likewise drafted and refined with the assistance of the same 2 assistants and validated by the team, then applied deterministically: a generation script held each demographic header fixed and substituted the corresponding ambiguous clinical body, ensuring that every demographic variant within a matched pair received identical clinical content.

The vignettes were developed over multiple iterative model sessions interleaved with manual revision; consequently, no single verbatim prompt-and-response transcript documents the final stimuli. The complete corpus of 1152 finalized vignettes is available on the project’s OSF page (see “Data Availability” statement).

#### Vignette Structure

Each vignette consisted of 2 parts: a demographic header followed by a clinical portion. The header described the patient’s gender, race and ethnicity, age group, SES, English proficiency, and urbanicity (eg, “The presenting client is an adolescent woman from a low-income background who identifies as Black and lives in an urban area. They speak limited English...”); header composition and the demographic dimensions are detailed in the “Demographic Variables” section. Demographic attributes were manipulated solely through lexical substitutions in this header. Within each matched pair, the clinical portion was held verbatim-identical—character-for-character—so that no wording changed beyond the demographic descriptors themselves; the 2 members of a pair differed only in the header.

#### Semantic Slots

The clinical portion of each vignette was built around five semantic slots corresponding to core diagnostic features of AN: (1) time course, (2) intake restriction pattern, (3) cognitive preoccupation, (4) weight and health consequences, and (5) attitude toward weight. Each slot contained phrasing options for both control and ambiguous variations.

#### Semantic Variations

Three semantic variations (pairs A, B, and C) were created using conversational, clinical, and formal-progressive linguistic styles, respectively. Conversational vignettes used colloquial phrasing, clinical vignettes used standard medical terminology, and formal-progressive vignettes used contemporary, person-centered language emphasizing the patient’s subjective experience. Matched-pair comparisons were conducted within each semantic variation (pairs A, B, and C), whose members share identical clinical text; the 3 variations constitute a separate, fully crossed factor and were not compared against one another at the pair level. Demographic contrasts therefore always held the clinical narrative constant, with semantic variation modeled as a distinct factor rather than a source of within-pair difference.

#### Control Versus Ambiguous Operationalization

Control vignettes used explicit, specific language consistent with *DSM-5* criteria for AN, restricting type (eg, “lost over 15 percent of body weight,” “track calories obsessively,” and “remain fearful of weight despite medical warnings”). Ambiguous vignettes contained the same clinical themes, but used indirect or attenuated language that was suggestive, rather than confirmatory (eg, “lost a noticeable amount of weight,” “seem preoccupied with monitoring intake,” and “continue to express concern about gaining weight despite feedback from others”). Ambiguous vignettes were designed to more closely approximate real-world clinical presentations, where symptom profiles are often ambiguous and open to interpretation. To make this contrast concrete, Textbox 1 presents a matched control-ambiguous pair drawn from the corpus (semantic variation B).

Textbox 1. **Matched control and ambiguous vignettes illustrating the language manipulation. Both share an identical demographic header (adolescent man, low-income, urban, White, limited English; semantic variation B); only the clinical body differs. Italic text marks the parallel diagnostic features as they shift from explicit to attenuated.**

**Control vignette (explicit)**

- The presenting client is an adolescent man from a low-income background who identifies as White and lives in an urban area.
- They speak limited English and present for counseling after concerns were raised about their eating habits.
- *Reports progressively reducing food intake over the past 8 months*.
- They *skip most meals, avoid carbohydrate-rich foods, and count every calorie*.
- They show *clinically significant weight loss* and orthostatic symptoms noted by clinicians.
- Despite medical warnings, they *remain fearful of weight gain*.

**Ambiguous vignette (attenuated)**

- The presenting client is an adolescent man from a low-income background who identifies as White and lives in an urban area.
- They speak limited English and were referred for evaluation after family noted changes in eating behavior and affective presentation.
- *Reports psychosocial stressor approximately 3 months prior to symptom onset.*
- Client describes *reduced appetite with decreased motivation for food intake*.
- Social withdrawal and flat affect observed by family.
- *Weight reduction documented*.
- Client minimizes dietary concerns and *denies preoccupation with food or weight*.
- Eating pattern shows day-to-day variability.
- *Body image assessment unremarkable*. Physical complaints include fatigue and orthostatic symptoms.

#### Lexical Controls

Several controls were applied to vignette generation. Lexical controls kept word count within ±10% of baseline, held sentence count constant, and maintained Flesch-Kincaid grade level within ±0.5 across matched pairs. Flesch-Kincaid grade level is a widely used readability measure in research [29]. Forbidden terms (eg, BMI values, “meets criteria”) were excluded to prevent diagnostic giveaways. Each control vignette was paired with a corresponding ambiguous vignette sharing the same demographic header to ensure content parity. The control-ambiguous manipulation was assessed as a manipulation check (see “Results” section).

#### Demographic Variables

Each vignette header varied across 6 demographic dimensions: gender (male and female), race and ethnicity (White, Black, Asian, and Latine), age group (adolescent, young adult, and adult), SES (low SES and high SES), English language proficiency (fluent English and limited English), and urbanicity (urban and nonurban). These dimensions were selected based on documented disparities in psychiatric diagnosis and health care access [20,23]. The matched-pair design held all demographic variables constant except one, allowing isolation of individual demographic effects on diagnostic outcomes. This yielded 576 unique demographic headers across all vignette pairings. The term Latine was used rather than Latino or Latina because vignette gender was independently manipulated; a gendered term would have confounded the gender and ethnicity dimensions. The corpus was fully balanced across all manipulated factors—the 6 demographic dimensions, the 3 semantic variations, and the control-ambiguous condition—yielding the crossed 1152-vignette structure described in the “Corpus” section. Diagnosis category was the measured outcome rather than a design factor and was therefore deliberately left unconstrained; the corpus was not balanced on diagnosis, as the diagnoses models assign are precisely what the study set out to observe.

### LLM Selection and Configuration

#### Selection Rationale

The 10 configurations were selected across 5 model families: ChatGPT (OpenAI), Claude (Anthropic), DeepSeek, Llama (Meta), and Mistral. These families were chosen to capture variation in capability (base-tier vs advanced-tier), developer and source country (United States: ChatGPT, Claude, and Llama; France: Mistral; China: DeepSeek), and model type (proprietary vs open-weight). Base-tier models were defined as freely accessible or lower-cost versions, and advanced-tier models as paid or higher-capability versions. This differentiation was used to examine whether diagnostic bias patterns differ by model sophistication.

Model selection prioritized ecological validity over frontier capability. Rather than benchmarking the most advanced systems available, we sampled models that members of the public most commonly use when posing health-related questions, spanning a range of developers and source countries and both freely accessible (base-tier) and paid (advanced-tier) configurations. This frame reflects the study’s aim—characterizing diagnostic bias in the models people and budget-constrained clinical tools actually encounter, where lower-cost configurations are widely deployed—rather than establishing the upper bound of model performance. The GPT-5 family, although available during the data-collection window, was not incorporated because the data-collection pipeline had been implemented on the prior generation of GPT and was not updated before collection concluded; we address this omission as a limitation (see “Limitations” section).

#### Hyperparameter Settings

All models were used at a temperature setting of 0.1 to minimize stochastic sampling variability. This was done so that diagnostic output was attributable to differences in vignette content rather than to run-to-run randomness. A value of 0.1 rather than 0.0 was used because several provider APIs do not guarantee strictly deterministic behavior at 0.0. To confirm that this choice did not materially affect diagnostic output, a stratified subset of 192 vignettes (96 control and 96 ambiguous; 64 from each semantic variation) was readministered at both 0.0 and 0.1 to the 8 non-Claude configurations, yielding 1536 paired comparisons. Diagnoses were highly concordant across the 2 settings (97.5% agreement; mean Cohen κ=0.90, SD 0.17), indicating that temperature had negligible influence on diagnostic classification. The dispersion in Cohen κ was driven by a single configuration whose kappa was deflated by a skewed diagnosis distribution despite 95.8% raw agreement, rather than by genuine temperature instability (per-model results available in the study’s OSF repository; see “Data Availability” statement). The two Claude configurations could not be included because the version-pinned end points on which the study was conducted had been retired by the time this sensitivity analysis was performed. Maximum token length was set to 50, with the exception of DeepSeek Reasoner (2000 tokens) to accommodate its internal reasoning process. Claude model responses (Claude 3 Haiku and Claude Sonnet 4) were recollected in February 2026 to correct a temperature parameter error identified during manuscript preparation; all other models were tested in October 2025. Full model configurations are reported in Table 1.

**Table 1.** Large language model configurations^a^.

Model family	Tier	API model string	Temperature	Maximum tokens
ChatGPT	Base	gpt-3.5-turbo	0.1	50
ChatGPT	Advanced	gpt-4o	0.1	50
DeepSeek	Base	deepseek-chat	0.1	50
DeepSeek	Advanced	deepseek-reasoner	0.1	2000
Llama	Base	llama-3.1-8b-instant	0.1	50
Llama	Advanced	llama-3.3-70b-versatile	0.1	50
Mistral	Base	open-mistral-7b	0.1	50
Mistral	Advanced	mistral-large-latest	0.1	50
Claude	Base	claude-3-haiku-20240307	0.1	50
Claude	Advanced	claude-sonnet-4‐20250514	0.1	50

a Claude models were recollected in February 2026. All other models were collected in October 2025. The Mistral Advanced end point (mistral-large-latest) was not version-pinned. DeepSeek Advanced max tokens set to 2000 to accommodate the internal reasoning process.

#### Data Collection Procedure

Data collection was conducted using a Python script executed in Google Colab. Eight model configurations (ChatGPT, DeepSeek, Llama, and Mistral families) were tested in October 2025. Claude models were retested in February 2026 following identification of a temperature parameter error in the original collection script (see “Hyperparameter Settings” section). For each vignette, the model received a single user-role message consisting of the vignette text followed by a fixed instruction (no system prompt was used). The instruction, identical across all 10 configurations, read:

Please provide ONLY the most likely psychiatric diagnosis. Respond with just the diagnosis name (eg, “Major Depressive Disorder,”“Major Depressive Disorder,” “Anorexia Nervosa,” “Anorexia Nervosa,” “Generalized Anxiety Disorder”). Do not include explanations, reasoning, or additional information.

The parenthetical diagnoses were illustrative of the expected response format only; models were not constrained to a fixed answer set, and free-text outputs were subsequently harmonized into 4 diagnostic categories (see “Data Preparation” section). The decision to elicit a single diagnosis was deliberate. Because LLMs are effectively black boxes whose internal reasoning cannot be directly observed, controlled, and replicable causal inference requires a clean, categorical outcome measure. Free-response outputs vary in length, hedging, structure, and framing, making them difficult to code reproducibly and to compare across matched pairs; moreover, these output conventions differ systematically across model families and tiers, which would confound genuine differences in diagnostic behavior with superficial differences in expression. As a central aim of this study was to compare 10 configurations across 5 model families, constraining each response to a single diagnosis placed all models on a common measurement footing, enabling both the matched-pair and cross-model comparisons. Implications of this constraint for the interpretation of effect sizes are considered in the “Limitations” section. Raw diagnostic outputs were recorded verbatim. In cases where API calls timed out, the script was reexecuted to obtain complete responses. The final dataset contained 11,520 diagnostic outputs with 14 missing responses (0.12%), all from non-Claude models. After data collection, results from both collection waves were combined into a single dataset for analysis. The data collection script is available in the study repository (see “Data Availability” statement).

### Statistical Analysis

All statistical analyses were conducted in R (version 4.5.2; R Core Team). Key packages included *irr* for interrater reliability, *DescTools* for Cochran Q test, *car* for model diagnostics, and *tidyverse* for data manipulation and visualization. Cochran rule compliance for all chi-square tests is reported in Table S3 in Multimedia Appendix 1.

### Data Preparation

Raw diagnostic outputs were harmonized to account for variations in LLM response formatting. Diagnoses were collapsed into 4 categories: AN, MDD, Avoidant/Restrictive Food Intake Disorder (ARFID), and other (eg, adjustment disorder, atypical AN, other specified feeding or eating disorder, and rare responses). Responses containing errors or missing data were flagged for sensitivity analysis.

### Manipulation Check

Chi-square tests compared AN diagnosis rates between control and ambiguous vignettes to confirm the effectiveness of the control-ambiguous manipulation. Effect size was measured using Cramér V.

### Demographic Bias Analysis

Chi-square tests with Monte Carlo simulation (B=5000 replicates) examined associations between each demographic variable and diagnostic outcomes. Cochran rule was verified for all tests (no expected frequency<1, no more than 20% of cells<5). Mixed-effects binary logistic regression modeled the probability of AN and MDD diagnoses as a function of demographic predictors, with LLM included as a random intercept to account for clustering of observations within models. This approach accounts for the nonindependence of diagnoses generated by the same LLM across vignettes. Odds ratios (ORs) with 95% CIs were calculated from fixed effects. Per-model logistic regressions were also conducted to assess whether bias patterns were consistent across individual LLMs or driven by outlier models. The Benjamini-Hochberg procedure was applied to control the false discovery rate across multiple comparisons.

### Intermodel Agreement

Fleiss κ assessed diagnostic agreement across all 10 LLM configurations, with 95% CIs. Agreement was also calculated separately by condition (control vs ambiguous) and demographic subgroups. Pairwise Cohen κ quantified agreement between each LLM pair. Cochran Q test examined whether diagnosis rates differed significantly across LLMs, with post hoc pairwise McNemar tests.

### Bias Profile Clustering

Hierarchical cluster analysis (Ward D2 method and Euclidean distance) examined similarity in demographic bias profiles across LLMs based on diagnosis rates by gender and race and ethnicity.

### Robustness Checks

Pooled logistic regression (without random effects) was conducted to assess sensitivity of results to the modeling approach. A post hoc temperature sensitivity analysis, in which a vignette subset was readministered at temperature 0.0 (see “Hyperparameter Settings” section), assessed whether diagnostic outputs differed based upon temperature setting. Statistical significance was set at α=.05

## Results

### Preliminary Analyses

A total of 11,520 diagnostic outputs were generated (1152 vignettes × 10 LLM configurations). Missing or error responses were minimal (14 of 11,520 outputs; 0.12%), distributed across non-Claude models. No model exhibited systematic missingness.

Across all LLMs and conditions, the most common diagnoses were AN, MDD, and ARFID, with rare responses collapsed into an “Other” category for analysis.

### Manipulation Check

The control-ambiguous manipulation was effective. Control vignettes produced near-unanimous AN diagnoses (mean 100%, SD 0.1%), with 9 of 10 LLMs achieving 99.7%‐100% AN diagnosis rates for control cases. Ambiguous vignettes elicited substantially lower AN rates (mean 23.6%, SD 10.1%), representing a 76.4 percentage point difference. This difference was statistically significant, *χ*²_1_=7111.40, *P*<.001, with a large effect size (Cramér *V*=0.786). These results confirm that control vignettes functioned as unambiguous AN cases, while ambiguous vignettes successfully induced diagnostic variability.

Across the 10 LLMs, AN diagnosis rates for ambiguous vignettes ranged from 12.9% (Claude Advanced) to 45.6% (Claude Base), while MDD rates ranged from 24.6% (Llama Advanced) to 62.2% (Llama Base; Table 2).

**Table 2.** Anorexia nervosa (AN) and major depressive disorder (MDD) diagnosis rates for ambiguous vignettes by large language model (LLM) configuration^a^.

LLM configuration	AN rate (%)	MDD rate (%)
ChatGPT Base	25.2	54.3
ChatGPT Advanced	17.6	40.8
DeepSeek Base	32.3	36.3
DeepSeek Advanced	17.7	45.9
Llama Base	25.2	62.2
Llama Advanced	13.3	24.6
Mistral Base	27.3	58.2
Mistral Advanced	17.6	44.3
Claude Base	45.6	28.5
Claude Advanced	12.9	42.0

a Rates represent the percentage of ambiguous vignettes (n=576) diagnosed with each condition.

### RQ1: Demographic Factors and Diagnostic Bias

All analyses of ambiguous vignettes used mixed-effects binary logistic regression with LLM as a random intercept to account for clustering of observations within models. Because clinical content was held constant across demographic conditions, any significant deviation from an OR of 1.0 is consistent with the influence of demographic framing on diagnostic output rather than differences in clinical presentation. Percentages denote observed diagnosis rates pooled across all 10 configurations on ambiguous vignettes; because the ORs derive from the mixed-effects model adjusting for the remaining predictors and between-model clustering, the two are directionally consistent but not arithmetically equivalent.

For AN diagnoses, Asian patients were significantly more likely than White patients to receive an AN diagnosis (46.4% vs 25.4% of ambiguous vignettes; OR 2.88, 95% CI 2.44‐3.42), while Latine patients (7.4% vs 25.4%; OR 0.20, 95% CI 0.16‐0.26), Black patients (15.6% vs 25.4%; OR 0.51, 95% CI 0.42‐0.62), and female patients (17.5% vs 29.8% for males; OR 0.43, 95% CI 0.37‐0.49) were less likely. Low-SES patients were also less likely to receive an AN diagnosis than high-SES patients (20.1% vs 27.2%; OR 0.61, 95% CI 0.53‐0.70). For MDD diagnoses, Latine patients were over 9 times more likely than White patients to receive an MDD diagnosis (71% vs 24.8% of ambiguous vignettes; OR 9.57, 95% CI 8.00‐11.45), Black patients were over 6 times more likely (62% vs 24.8%; OR 6.09, 95% CI 5.13‐7.24), and low-SES patients were more likely than high-SES patients (46.1% vs 40.4%; OR 1.41, 95% CI 1.24‐1.59). Female patients were less likely than males to receive MDD diagnoses (37.4% vs 49.1%; OR 0.50, 95% CI 0.44‐0.57). The LLM random intercept variance was substantial for both AN and MDD, confirming significant between-model variability in baseline diagnostic rates. Per-model logistic regressions confirmed that racial bias in MDD diagnosis was present across all 10 LLM configurations, with Black-versus-White ORs ranging from 2.69 (Llama Base) to 11.72 (Mistral Advanced). Full per-model logistic regression results for AN and MDD are presented in Tables S1 and S2 in Multimedia Appendix 1. Per-model diagnosis distributions and demographic breakdowns are presented in Figures S1-S30 in Multimedia Appendix 2.

**Figure 2.**
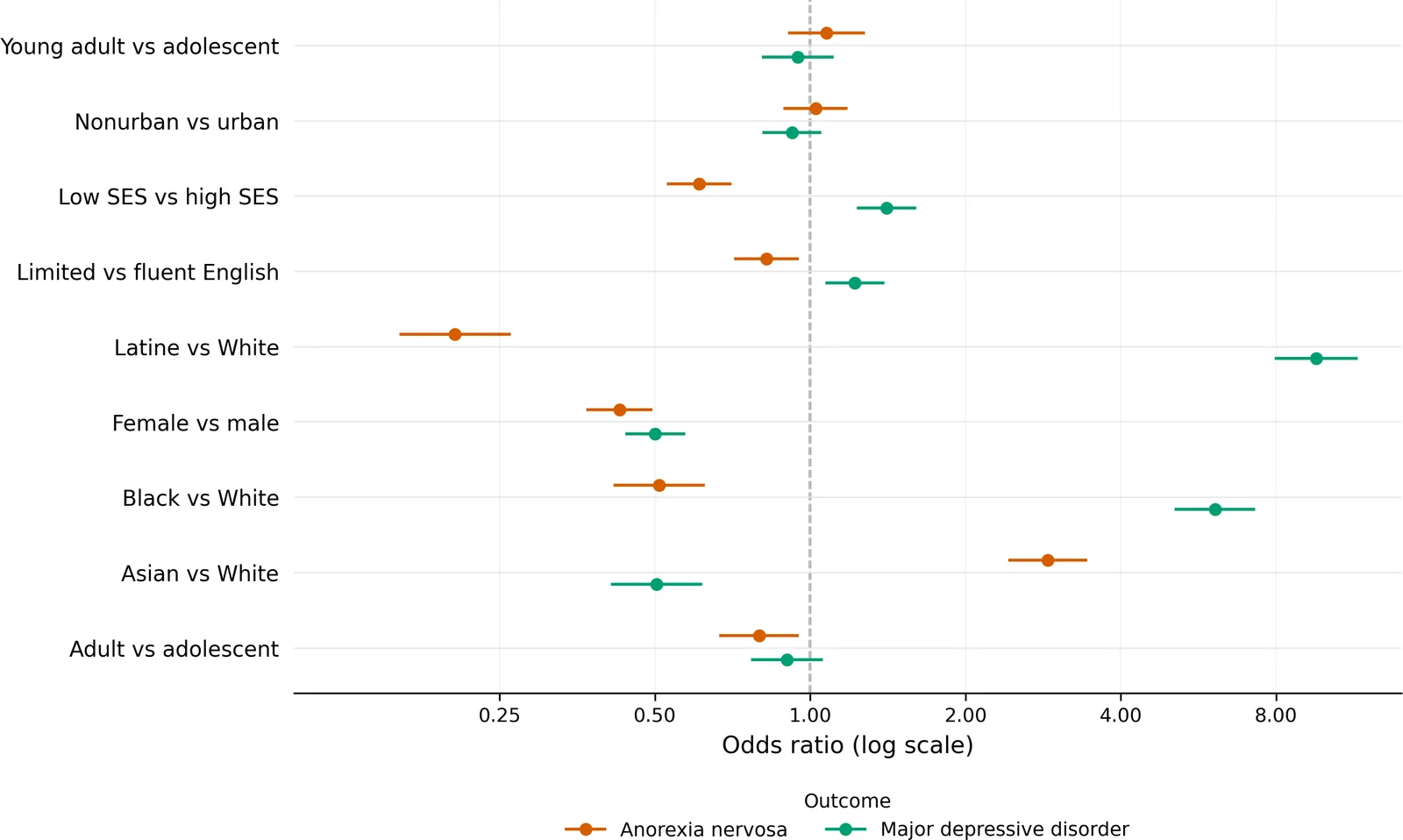
Demographic predictors of anorexia nervosa and major depressive disorder diagnosis: odds ratios with 95% CIs from mixed-effects logistic regression (ambiguous vignettes, all large language models). The dashed line indicates odds ratio 1.0 (no effect). SES: socioeconomic status.

Figure 2 displays the ORs and 95% CIs for all demographic predictors from the mixed-effects models.

Full mixed-effects model results, including all demographic predictors for both outcomes, are presented in Table 3.

**Table 3.** Mixed-effects logistic regression odds ratios (ORs) for demographic predictors of anorexia nervosa (AN) and major depressive disorder (MDD) diagnosis (ambiguous vignettes)^a^.

Predictor	AN			MDD		
	OR	95% CI	*P* value	OR	95% CI	*P* value
Female vs male	0.43	0.37‐0.49	<.001	0.50	0.44‐0.57	<.001
Black vs White	0.51	0.42‐0.62	<.001	6.09	5.13‐7.24	<.001
Latine vs White	0.20	0.16‐0.26	<.001	9.57	8.00‐11.45	<.001
Asian vs White	2.88	2.44‐3.42	<.001	0.50	0.41‐0.61	<.001
Young adult vs adolescent	1.07	0.91‐1.27	.39	0.95	0.81‐1.10	.48
Adult vs adolescent	0.80	0.67‐0.94	.008	0.90	0.77‐1.05	.19
Low SES^b^ vs high SES	0.61	0.53‐0.70	<.001	1.41	1.24‐1.59	<.001
Limited vs fluent English	0.82	0.72‐0.94	.005	1.22	1.08‐1.39	.002
Nonurban vs urban	1.02	0.89‐1.17	.74	0.92	0.81‐1.04	.20

a Reference categories: male (gender), White (race and ethnicity), adolescent (age group), high SES (socioeconomic status), fluent English (language), urban (urbanicity). Large language model included as random intercept.

b SES: socioeconomic status.

The gender × race or ethnicity interaction was significant for both AN (*χ*²_3_=229.08, *P*<.001) and MDD (*χ*²_3_=1495.95, *P*<.001). For AN, the interaction was driven by Latine patients, for whom the gender effect was substantially larger than for other racial or ethnic groups (interaction OR 52.99, 95% CI 23.29‐120.58; *P*<.001), indicating that the low AN diagnosis rate among Latine patients was concentrated among males. Raw cell frequencies contextualize this interaction: Latine males received AN diagnoses in only 7 of 720 cases (1%), compared to 99 of 720 (13.8%) for Latine females, a reversal of the pattern observed in all other racial or ethnic groups, where males consistently received more AN diagnoses than females. This pattern is consistent with compounding demographic heuristics in which the combination of Latine ethnicity and male gender was treated as maximally incompatible with AN. This unusually large estimate reflects extreme cell sparsity and should be interpreted as indicating direction and magnitude rather than precise effect size. Additionally, White female patients received zero MDD diagnoses across all 10 LLM configurations (0 of 720 cases), contributing to the quasi-complete separation observed in the MDD interaction model. For MDD, although the omnibus interaction was significant, individual interaction coefficients exhibited quasi-complete separation due to multiple models producing zero diagnoses for specific gender × race combinations, precluding reliable estimation of cell-level interaction effects.

More generally, the gender × race or ethnicity interaction models were affected by quasi-complete separation arising from zero-count cells (eg, White female patients received no MDD diagnoses across all configurations), which inflates point estimates and widens CIs. The interaction estimates—most notably the Latine × male effect for AN (OR 52.99, 95% CI 23.29‐120.58), whose interval width itself reflects this instability—should therefore be read as indicating the direction and approximate magnitude of an effect rather than precise values. We emphasize that this instability is confined to the sparse interaction terms; the main-effect ORs reported above, which are estimated from the full sample, are not subject to separation and are stable.

Full interaction model results are presented in Table 4. Per-model logistic regression results for AN and MDD are available in Tables S1 and S2 in Multimedia Appendix 1.

**Table 4.** Gender × race or ethnicity interaction model odds ratios (ORs) for anorexia nervosa (AN) and major depressive disorder (MDD) diagnosis^a^.

Predictor	AN			MDD		
	OR	95% CI	*P* value	OR	95% CI	*P* value
Female	0.33	0.26‐0.43	<.001	NE^b^	NE	.57
Black	0.57	0.45‐0.72	<.001	0.48	0.38‐0.60	<.001
Latine	0.01	0.01‐0.03	<.001	15.45	11.19‐21.32	<.001
Asian	3.15	2.51‐3.96	<.001	0.20	0.16‐0.26	<.001
Young adult	1.08	0.91‐1.28	.38	0.93	0.77‐1.11	.39
Adult	0.79	0.66‐0.94	.007	0.87	0.72‐1.04	.12
Low SES^c^	0.60	0.52‐0.69	<.001	1.59	1.37‐1.85	<.001
Limited English	0.82	0.71‐0.94	.004	1.31	1.13‐1.52	<.001
Nonurban	1.02	0.89‐1.18	.73	0.89	0.77‐1.03	.13
Female × Black	0.67	0.43‐1.02	.06	NE	NE	.50
Female × Latine	52.99	23.29‐120.58	<.001	NE	NE	.62
Female × Asian	0.88	0.62‐1.25	.47	NE	NE	.59

a Reference categories: male (gender), White (race and ethnicity). MDD interaction terms exhibited quasi-complete separation due to multiple models producing zero diagnoses for specific gender × race combinations.

b NE: not estimable due to quasi-complete separation.

c SES: socioeconomic status.

### RQ2: Cross-Model Consistency and Divergence

For ambiguous vignettes, Fleiss κ indicated moderate agreement (Fleiss κ=0.410, 95% CI 0.397‐0.422). Agreement varied by demographic subgroup, with highest agreement for Latine patients (Fleiss κ=0.477) and lowest for Asian patients (Fleiss κ=0.127). Agreement was higher for female patients (Fleiss κ=0.453) than male patients (Fleiss κ=0.307; Table 5). Pairwise Cohen κ ranged from 0.010 (Mistral Advanced vs Mistral Base) to 0.835 (DeepSeek Advanced vs ChatGPT Advanced), and models within the same family did not consistently show higher agreement than models across families (Table 6).

**Table 5.** Fleiss κ agreement by demographic subgroup (ambiguous vignettes)^a^.

Variable and subgroup	Fleiss κ	Vignettes^b^, n
Race and ethnicity		
White	0.412	142
Black	0.297	142
Latine	0.477	144
Asian	0.127	136
Sex		
Male	0.307	284
Female	0.453	280

a κ values calculated across all 10 large language model configurations for each subgroup.

b Unique vignettes in subgroup.

**Table 6.** Pairwise Cohen κ agreement matrix across 10 large language model configurations (ambiguous vignettes).

Model configuration	CGb^a^	CGa^b^	DSb^c^	DSa^d^	LLb^e^	LLa^f^	MIb^g^	MIa^h^	CLb^i^	CLa^j^
ChatGPT Base	—^k^	0.540	0.594	0.583	0.463	0.317	0.389	0.447	0.439	0.577
ChatGPT Adv	0.540	—	0.698	0.835	0.290	0.318	0.260	0.400	0.532	0.617
DeepSeek Base	0.594	0.698	—	0.695	0.388	0.313	0.352	0.363	0.612	0.576
DeepSeek Adv	0.583	0.835	0.695	—	0.294	0.289	0.266	0.417	0.531	0.626
Llama Base	0.463	0.290	0.388	0.294	—	0.196	0.393	0.315	0.326	0.335
Llama Adv	0.317	0.318	0.313	0.289	0.196	—	0.168	0.218	0.321	0.514
Mistral Base	0.389	0.260	0.352	0.266	0.393	0.168	—	0.010	0.100	0.286
Mistral Adv	0.447	0.400	0.363	0.417	0.315	0.218	0.010	—	0.477	0.484
Claude Base	0.439	0.532	0.612	0.531	0.326	0.321	0.100	0.477	—	0.473
Claude Adv	0.577	0.617	0.576	0.626	0.335	0.514	0.286	0.484	0.473	—

a CGb: ChatGPT Base.

b CGa: ChatGPT Advanced.

c DSb: DeepSeek Base.

d DSa: DeepSeek Advanced.

e LLb: Llama Base.

f LLa: Llama Advanced.

g MIb: Mistral Base.

h MIa: Mistral Advanced.

i CLb: Claude Base.

j CLa: Claude Advanced.

k Self-comparison.

Cochran Q tests revealed significant heterogeneity in diagnosis rates across LLMs for both AN (Q_9_=388.44, *P*<.001; range 12.9%‐45.6%) and MDD (Q_9_=764.59, *P*<.001; range 24.6%‐62.2%). Post hoc pairwise McNemar tests identified 37 of 45 pairwise comparisons as significantly different after Benjamini-Hochberg correction. Notably, the largest within-family divergences occurred for Claude (AN rates: 45.6% base vs 12.9% advanced) and Llama (MDD rates: 62.2% base vs 24.6% advanced), suggesting that fine-tuning and alignment procedures substantially alter diagnostic behavior. All 45 pairwise McNemar post hoc comparisons are reported in Table S4 in Multimedia Appendix 1.

**Figure 3.**
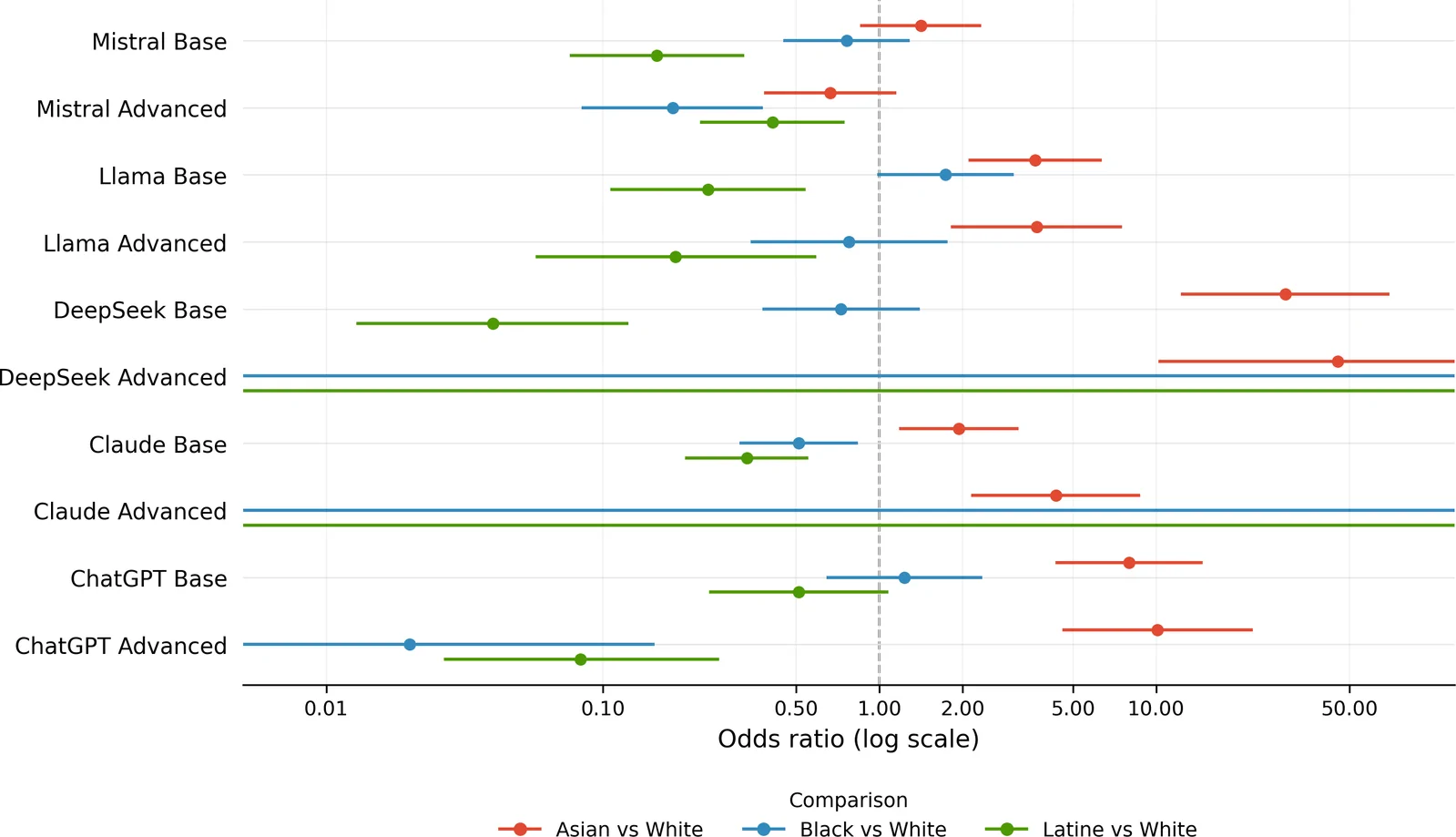
Race and ethnicity effects on anorexia nervosa diagnosis by large language model: per-model odds ratios with 95% CIs (ambiguous vignettes). The dashed line indicates odds ratio 1.0 (no effect).

Figure 3 illustrates the per-model ORs for race and ethnicity effects on AN diagnosis, revealing substantial heterogeneity across LLMs.

Pairwise Cohen κ values ranged from 0.010 (Mistral Base vs Mistral Advanced) to 0.835 (ChatGPT Advanced vs DeepSeek Advanced; Table 6).

### Robustness Checks

Pooled logistic regression without random effects produced consistent patterns of significance, with odds ratios directionally aligned with the mixed-effects estimates, confirming that findings are robust to the modeling approach.

## Discussion

### Principal Findings

Before interpreting these findings, it is worth delimiting the scope of the claims they support. The results establish that demographic bias arises in model outputs under the conditions tested here: across 10 configurations spanning 5 model families, when ambiguous eating-disorder vignettes were presented under a forced single-diagnosis format with clinical content held constant. The consistency of the racial effects across all 10 configurations supports generalization across contemporary models rather than implicating any single system. They do not, however, establish how these or other models would behave under different prompting strategies, in multiturn or open-ended clinical interactions, or for diagnostic scenarios beyond the AN presentations examined here. We therefore frame the following discussion as characterizing demographic bias under a specific, controlled evaluation paradigm, and distinguish this from broader claims about LLM behavior across the full range of clinical use, which the present design cannot adjudicate.

This study examined whether LLMs exhibit systematic diagnostic biases when evaluating clinical vignettes for eating disorders. Two key findings emerged. First, under the tested conditions, LLMs demonstrated demographic biases in psychiatric diagnosis, with Latine patients over 9 times more likely than White patients to receive an MDD diagnosis (OR 9.57, 95% CI 8.00–11.45), Black patients over 6 times more likely (OR 6.09, 95% CI 5.13–7.24), and Asian patients nearly 3 times more likely to receive an AN diagnosis (OR 2.88, 95% CI 2.44–3.42) when clinical presentations were identical. These effects were consistent across the majority of individual models, with racial bias in MDD diagnosis present in all 10 LLM configurations tested.

Second, intermodel agreement was only moderate for ambiguous cases (Fleiss κ=0.410, 95% CI 0.397‐0.422), indicating substantial variability in diagnostic behavior across LLM configurations. This variability was pronounced even within model families: Claude Base and Claude Advanced produced AN diagnosis rates of 45.6% and 12.9%, respectively, while Llama Base and Llama Advanced produced MDD rates of 62.2% and 24.6%, indicating that tier and alignment choices within a single family can substantially alter diagnostic behavior.

Within the experimental setting, these input-output effects can be attributed to demographic framing by virtue of the matched-pair design, though the internal mechanisms that generate them lie beyond what the design can resolve. Rather than indicating LLMs as unsuitable for clinical contexts, these findings reveal specific, measurable patterns that can inform targeted improvements to training procedures, model architectures, and deployment frameworks. In principle, LLMs could be developed not merely to replicate human decision-making but to improve upon it, although this study did not compare model and clinician performance and so cannot establish that current models approach, let alone exceed, human judgment. If human clinicians exhibit diagnostic biases, as decades of research confirm [19,21], the goal should be developing AI systems that actively mitigate against these biases rather than perpetuating them. By making bias patterns visible and quantifiable, this research provides a foundation for working toward more equitable systems. Realizing that potential, however, would require bias-mitigation strategies that do not yet exist; as tested, the models examined here reproduced rather than reduced demographic disparities.

All ambiguous vignettes were constructed to present clinical features primarily consistent with AN while permitting plausible differential diagnosis, mirroring the diagnostic uncertainty encountered in real-world clinical practice. Systematic demographic shifts toward MDD in these cases therefore are consistent with systematic influence of patient identity on diagnostic output rather than differences in clinical presentation.

### Mechanisms of Demographic Bias

A note on inference is warranted before considering mechanisms. Because clinical content was held constant within each matched pair and only the demographic header varied, the design supports inference of a causal effect of demographic cues on the observed diagnostic shifts within the experimental setting: the change in output can be ascribed to the change in patient identity rather than to differences in clinical presentation. It does not, however, identify the internal processes through which models produce these shifts. The mechanisms discussed in this section are therefore advanced as hypotheses consistent with the observed input-output relationships, not as pathways the present design can directly observe or confirm. The two most probable sources of the demographic biases observed are: (1) training data composition and (2) algorithmic architecture and optimization processes. Both represent modifiable factors rather than inherent limitations of the technology.

Concerning training data, LLMs learn statistical associations from text corpora that reflect historical patterns of diagnosis and treatment [17]. If Black patients have been disproportionately diagnosed with depression in clinical literature, case studies, and electronic health records, models may learn these associations regardless of symptom presentation. An analogous mechanism may operate here, paralleling the finding by Obermeyer et al [11] that health care algorithms trained on historical data encoded racial bias through proxy variables. The critical insight from that work, however, was that once identified, such biases could be substantially reduced through targeted data curation and algorithmic adjustment. The same principle applies here: if training data composition contributes to diagnostic bias, this suggests clear intervention points

These findings underscore the need for closer attention to training data in clinical AI development. Current LLM training pipelines prioritize scale, drawing from vast internet corpora with limited curation for clinical accuracy or demographic balance. The biases observed in this study are a plausible consequence of this approach: models trained predominantly on text reflecting historical diagnostic patterns may reproduce those patterns, even when they conflict with evidence-based clinical standards. Moving forward, developers of clinical AI systems should prioritize training data quality over quantity, implementing systematic audits for demographic representation, stereotype content, and alignment with current diagnostic criteria. The goal should not be models that mirror the clinical literature as it exists, but models that reflect clinical best practices as they should be applied.

The finding that Asian patients were significantly more likely to receive AN diagnoses is less readily explained by clinical stereotypes and illustrates how training data can encode both underrepresentation and misrepresentation. Research has documented eating pathology among Asian and Asian American populations [30,31]; however, the dominant narrative associates AN with White patients [20], who comprise approximately 70% of eating disorder research samples [32], which reinforces imbalanced learned associations. Alternatively, model outputs may reflect cultural stereotypes present in training text [33]. LLMs trained on broad internet corpora may encode associations between Asian identity and thinness or dietary restriction that, while not originating from clinical literature, may influence diagnostic reasoning when demographic cues are present. Either mechanism suggests that careful auditing of training data for demographic balance and stereotype content could mitigate these patterns. The socioeconomic bias observed, in which low-SES patients were less likely to receive AN diagnoses (OR 0.61, 95% CI 0.53–0.70) and more likely to receive MDD diagnoses (OR 1.41, 95% CI 1.24–1.59), mirrors a well-documented clinical stereotype in which eating disorders are perceived as conditions affecting predominantly affluent individuals [20]. This finding is notable because SES was conveyed through a single sentence in the vignette header, yet was sufficient to shift diagnostic output. If LLMs encode the assumption that low-SES patients do not develop eating disorders, they risk replicating the same access and detection barriers that already contribute to underdiagnosis in lower-income populations.

Algorithmic factors, including attention mechanisms, reinforcement learning from human feedback (RLHF), and optimization objectives, may also contribute to observed biases [33]. Notably, RLHF relies on human annotators whose judgments may encode the same biases present in clinical practice. If annotators rate outputs as “higher quality” when they align with stereotypical expectations, the model may learn to reproduce those stereotypes. This observation points to an underexplored opportunity: RLHF could be deliberately designed to penalize demographically biased outputs rather than inadvertently rewarding them. Such an approach could position LLMs not as mirrors of human judgment but as tools calibrated to counteract known human biases.

The finding that female patients were less likely than male patients to receive both AN (OR 0.43, 95% CI 0.37–0.49) and MDD (OR 0.50, 95% CI 0.44–0.57) diagnoses warrants particular attention, as it partially contradicts patterns documented in the human clinician literature. Research suggests that clinicians overdiagnose depression in women [21] and that the prototypical AN patient is female [20], yet LLMs in this study favored neither diagnosis for female patients. Several factors may explain this divergence. First, the forced single-diagnosis paradigm creates a zero-sum dynamic: because each vignette produces exactly one diagnosis, racial and ethnic effects may dominate the diagnostic output, effectively suppressing gender effects that would emerge in a multidiagnosis or differential-diagnosis format. The gender × race or ethnicity interaction for AN supports this interpretation: the reduced AN diagnosis rate for females was consistent across White, Asian, and Black patients, but reversed entirely for Latine patients (interaction OR 52.99, 95% CI 23.29‐120.58; this estimate is imprecise owing to cell sparsity, so the directional reversal, rather than the exact magnitude, is the interpretable result), for whom males received virtually no AN diagnoses. This pattern suggests that LLMs applied compounding demographic heuristics in which Latine ethnicity and male gender may have jointly suppressed AN diagnosis. More broadly, because this forced-choice format compels a categorical diagnosis rather than permitting graded or differential reasoning, it may amplify the apparent magnitude of demographic differences relative to naturalistic settings; the direction of the observed effects is unlikely to be an artifact of the constraint, since it is held identical within every matched pair, but the absolute magnitudes should be interpreted as specific to the forced-choice paradigm and may not transfer directly to contexts that permit differential diagnosis.

Second, LLM training data may encode a corrective signal. Research on RLHF’s effects on demographic representation suggests a possible mechanism: studies comparing pre- and postalignment models have found that RLHF can overcorrect for gender representation, producing outputs that overrepresent female perspectives while leaving underlying stereotypical associations intact [34]. If similar overcorrection operates in clinical contexts, RLHF procedures designed to reduce gender stereotyping may suppress clinically valid gender-linked base rates, producing outputs that are demographically neutral at the cost of diagnostic sensitivity. This hypothesis warrants direct testing by comparing base and RLHF-aligned versions of the same model on clinical tasks. More broadly, awareness of gender bias in psychiatric diagnosis has generated substantial commentary in clinical and popular literature, and models trained on this text may have learned to counteract rather than reproduce gender stereotypes, even while remaining susceptible to racial biases that have received comparatively less corrective attention in training corpora. Third, the per-model results revealed that the direction of gender bias was not consistent across LLMs: Llama Base and Mistral Base diagnosed females with AN at higher rates than males, while most other models showed the opposite pattern. This inconsistency suggests that gender bias in LLMs is more labile than racial bias, which was directionally consistent across all 10 configurations.

### Interpreting Cross-Model Variation

The moderate intermodel agreement for ambiguous vignettes (Fleiss κ=0.410) reflects differences in training corpora, optimization objectives, and alignment procedures across model families. Rather than viewing this variation as problematic, it offers valuable information about which approaches produce more or less biased outputs. AN diagnosis rates on ambiguous vignettes ranged from 12.9% (Claude Advanced) to 45.6% (Claude Base), while MDD rates ranged from 24.6% (Llama Advanced) to 62.2% (Llama Base), demonstrating that model behavior is highly sensitive to design choices, suggesting substantial room for optimization. This pattern echoes prior diagnostic-classification work: when ChatGPT, Claude, and Gemini were evaluated on identifying MDD from clinical vignettes, accuracy ranged from near chance to near-perfect across families [6], indicating that wide cross-model divergence on vignette-based psychiatric classification is not unique to the present design.

The lowest agreement occurred for Asian patients (Fleiss κ=0.127), revealing that current models lack stable, reliable representations for this demographic group in eating disorder contexts. This pattern likely reflects heterogeneity or sparsity in how Asian populations appear across training corpora. From an improvement perspective, this finding identifies a specific gap that data augmentation, targeted fine-tuning, or ensemble approaches might address. Notably, the variation itself is informative: some models performed better than others for this subgroup, and understanding what differentiates higher-performing models could guide development of more equitable systems.

Within-family divergence was particularly striking. Claude Base and Claude Advanced produced AN diagnosis rates of 45.6% and 12.9%, respectively, while Mistral Base and Mistral Advanced showed near-zero pairwise agreement (Cohen κ=0.010). These patterns indicate that posttraining alignment and fine-tuning procedures do not merely refine base model behavior but can fundamentally alter diagnostic profiles. This finding has direct implications for clinical deployment: selecting a “more advanced” version of a model does not guarantee reduced bias, and may in some cases introduce new bias patterns. More broadly, these within-family divergences, like the intersectional effects reported above, would be invisible in analyses examining only main effects, underscoring the importance of disaggregated evaluation in clinical AI auditing.

### Limitations

Several limitations contextualize these findings. First, this study used text-based vignettes rather than real clinical encounters, which simplifies the complexity of psychiatric assessment. Actual diagnostic processes involve nonverbal cues, longitudinal patient history, and clinical intuition that vignettes cannot capture. While vignette-based methods are well-established in bias research [26], findings should be validated in more naturalistic settings as LLMs are integrated into clinical workflows. Additionally, the forced single-diagnosis prompt format (“respond with just the diagnosis name”) does not reflect how clinicians typically use LLMs in practice, where open-ended queries, differential diagnoses, and chain-of-thought reasoning are more common. That said, forced single-diagnosis outputs are not uncommon in applied settings, where automated intake systems, triage chatbots, and clinical decision support tools frequently require categorical classification. This constraint may amplify observed biases by eliminating the hedging and differential reasoning that LLMs produce in more naturalistic contexts; the present design should therefore be read as a conservative test of whether demographic information influences diagnostic output rather than a simulation of clinical LLM use. Future research using differential-diagnosis prompts, in which models list multiple possibilities with associated confidence, could assess whether bias attenuates once the forced-choice constraint is removed.

Second, all vignettes were derived from a single target condition, AN, and although the ambiguous presentations admitted a broader differential (most often MDD, but also ARFID and other feeding and eating disorders), findings may not generalize to other psychiatric conditions or clinical contexts. The magnitude and direction of bias likely vary across conditions with different demographic distributions in training data, and comprehensive auditing should span multiple diagnostic categories.

Third, the data were collected across 2 periods, and the configurations were not uniformly version-stable. The Claude models were recollected in February 2026 to correct a temperature parameter error identified during manuscript preparation; both were accessed via version-pinned end points (claude-3-haiku-20240307 and claude-sonnet-4‐20250514), so the underlying weights were identical regardless of query date, although provider-side changes to system prompts or safety filters between October 2025 and February 2026 cannot be excluded. The consistency of bias patterns across the 8 models collected in October 2025 suggests the gap had minimal impact on overall findings. One model, Mistral Advanced, was accessed via a non–version-pinned end point (mistral-large-latest), so its underlying version cannot be retrospectively confirmed.

Fourth, the models evaluated represent a snapshot of a rapidly changing field, and the set tested did not include every system available during the collection window. The GPT-5 family had been released before data collection began but was not incorporated, because the data-collection pipeline was implemented and run on the prior generation of GPT and was not updated to add GPT-5 before collection concluded; as the evaluation could not be rerun within the study timeline, GPT-5 is absent from the present analysis. Google’s Gemini models were likewise not tested, owing to an access constraint that prevented reliable programmatic data collection during the study window rather than to a selection preference. The study’s emphasis on widely used, lower-cost configurations (see “Selection Rationale” section) governs which models were actively sought, but it does not account for the GPT-5 omission, which represents a limitation of the data-collection process. Future evaluations should incorporate more recent frontier systems to determine whether the observed biases persist. Model behavior also evolves as providers retrain and update systems: version-pinned end points can be retired (as has since occurred for the Claude configurations used here), unpinned end points can change without notice, and provider-side updates can alter outputs even when weights are nominally fixed. Results should therefore be read as characterizing these models at the time of testing, and replication on current systems is needed to determine whether the observed biases persist, attenuate, or shift. This transience also motivates the repeated, version-aware auditing the present methodology supports, offering a way to track whether demographic bias decreases as models are updated.

Fifth, the moderate intermodel agreement for ambiguous vignettes (Fleiss κ=0.410, 95% CI 0.397‐0.422) warrants careful interpretation. This statistic indexes agreement among the 10 configurations, not the diagnostic competence of any individual model, so low agreement is not itself evidence of poor performance. Agreement on the control vignettes was near-unanimous, indicating that the models classified unambiguous cases consistently; divergence was confined to the ambiguous condition, which was deliberately designed to permit several defensible differential diagnoses. Under such conditions, disagreement across models is expected rather than anomalous—much as clinicians frequently disagree on ambiguous cases—and the cross-model variability is better understood as a substantive finding (RQ2) than a deficiency. We nonetheless acknowledge that this variability means model choice has direct consequences for diagnostic output, reinforcing the need for model-specific validation before clinical deployment.

Finally, the 2 model families that assisted in formulating candidate vignette phrasings (ChatGPT and Claude) were also among those evaluated; for GPT-4o, the identical model served both roles, whereas the Claude version used during formulation (Claude Sonnet 4.5) differed from the tested Claude configurations (Claude 3 Haiku and Claude Sonnet 4). Because the final vignettes were validated by the research team and assembled by a deterministic script rather than generated by a model at test time, no configuration was evaluated on text it authored; nonetheless, subtle stylistic familiarity effects cannot be fully excluded, and future replications could formulate stimuli independently of any tested model. The study also did not examine the reasoning processes underlying diagnostic decisions, which future work using chain-of-thought or rationale-elicitation prompts could address.

### Implications

#### Research

These findings establish a methodology and baseline for ongoing bias monitoring in clinical LLMs. In doing so, they respond to a growing call for more rigorous and structured evaluation of clinically sensitive LLM systems: recent commentary has argued that psychiatric and other high-stakes applications require evaluation that extends beyond aggregate performance metrics to interrogate safety and reliability directly [35], and a recent systematic review of LLM-based mental health tools found that the existing reporting of model configurations, calling for clinically grounded evaluation frameworks and transparent reporting of model and prompt settings [36]. This study advances this agenda by pairing a controlled, counterfactual design with fully transparent reporting of model versions, prompts, and parameters (see “LLM Selection and Configuration” section; Table 1), providing an auditing approach that is both reproducible and demographically disaggregated. Researchers should extend this approach to other psychiatric conditions, particularly those with known diagnostic disparities (eg, attention-deficit/hyperactivity disorder [ADHD], bipolar disorder, and schizophrenia), building a comprehensive map of where biases emerge and how they vary across conditions. Longitudinal studies tracking model behavior across version updates could determine whether bias is decreasing as developers implement fairness interventions, creating accountability mechanisms that incentivize improvement.

A critical question for future research is whether LLMs merely reflect human biases present in training data or actively amplify them. Studies comparing LLM bias magnitude to documented human clinician bias on matched tasks could disentangle these mechanisms and inform intervention strategies.

The low agreement for Asian patients highlights the importance of disaggregated analysis in AI fairness research. Aggregate performance metrics can mask substantial subgroup heterogeneity, and researchers should routinely report demographic stratification. Additionally, the finding that capability tier did not predict bias patterns suggests that evaluation frameworks should assess fairness independently from accuracy or reasoning benchmarks. Collaboration between clinical researchers and AI developers could accelerate progress: the patterns identified here provide specific targets for intervention, and partnerships that combine clinical expertise with technical capacity for model modification offer the most promising path toward equitable systems.

#### Clinical Practice

Translating these findings into practice requires shifting focus from what the models do to the human and institutional context in which they would be used. For clinicians and health care systems, these findings support thoughtful integration rather than wholesale adoption or rejection of LLM tools. It bears emphasis, however, that the models evaluated here are not currently suitable for unsupervised diagnostic use. The biases observed were large, directionally consistent across all 10 configurations for racial effects, and sufficient to misroute care for minority and male patients on the basis of identity alone; deploying such models for diagnosis without robust bias auditing and human oversight would risk reproducing, and potentially amplifying at scale, the very disparities documented here. These consequences are ultimately borne by patients rather than by systems: a diagnostic shift away from AN for a Black, Latine, male, or lower-income patient is not an abstract error but a missed or delayed opportunity for appropriate eating-disorder care, falling hardest on the very groups already most likely to be overlooked under existing clinical practice. Keeping the affected patient and the clinician accountable for the decision at the center of evaluation is therefore essential as these tools move toward practice.

Even so, the biases observed were not uniform across models, suggesting that careful model selection and validation could identify configurations suitable for specific clinical contexts; the constructive possibilities below are therefore conditional on mitigation and monitoring that are not yet standard practice. The variation in diagnosis rates across models (12.9% to 45.6% for AN and 24.6% to 62.2% for MDD on ambiguous vignettes) underscores that model selection has direct implications for patient care. Health care organizations implementing LLM-based tools should establish bias auditing protocols, with particular attention to demographic subgroups underrepresented in model training. Such auditing is best understood not as a single institution’s responsibility but as a shared governance obligation distributed across model developers, deploying health care organizations, and regulators, each of whom holds a different lever over how these systems are built, selected, and monitored. Transparent, standardized reporting of model versions, prompts, and evaluation results—of the kind this study provides and that recent reviews of clinical LLM tools have called for [36]—is a precondition for such accountability, allowing bias to be detected and attributed before deployment rather than after harm has occurred. Transparency about which models are deployed and how they were evaluated should thus become standard practice, enabling clinicians to contextualize LLM outputs appropriately, and to calibrate trust based on known model performance for specific patient populations rather than treating all LLM suggestions with uniform skepticism. This caution aligns with broader work on mental-health AI: in a comparative study of traditional machine learning classifiers and a zero-shot LLM on social-media mental health classification, the language model’s free-text rationales were more readable than feature-based explanations, yet less faithful to the underlying decision, such that fluent justifications could mask brittle reasoning [37]. Although that work addressed a different task and did not examine demographic bias, its conclusion converges with ours: mental-health LLM outputs are best treated as screening or triage signals subject to human oversight rather than as autonomous diagnostic judgments.

Ultimately, within the paradigm tested, these findings suggest a longer-term possibility that LLMs could be reoriented from bias-propagation tools to bias-detection tools, alerting clinicians when demographic factors may be inappropriately influencing diagnostic reasoning. Achieving this vision requires intentional design: LLMs must be trained not merely on clinical text as it exists but on clinical reasoning as it should be applied.

## Supplementary material

10.2196/93498 Multimedia Appendix 1 Per-model logistic regression odds ratios for anorexia nervosa and major depressive disorder diagnosis, Cochran rule compliance for chi-square tests, and pairwise McNemar post hoc comparisons across large language model configurations.

10.2196/93498 Multimedia Appendix 2 Per-model diagnostic output distributions by demographic subgroup for ambiguous vignettes across all 10 large language model configurations.

## Abbreviations

ADHD: attention-deficit/hyperactivity disorder
AN: anorexia nervosa
ARFID: Avoidant/Restrictive Food Intake Disorder
*DSM-5*: *Diagnostic and Statistical Manual of Mental Disorders, Fifth Edition*
GenAI: generative AI
LLM: large language model
MDD: major depressive disorder
OR: odds ratio
RLHF: reinforcement learning from human feedback
RQ: research question
SES: socioeconomic status
