# Supplementary material for: Implicit Bias in Large Language Model Diagnosis of Eating Disorders: Experimental Vignette Study
Source: JMIR AI. 2026 Sep 23;5:e93498. doi: 10.2196/93498 (PMC13600661; doi:10.2196/93498)

Multimedia Appendix 2

Per-Model Diagnosis Distributions and Demographic Breakdowns (Figures S1–S30)

Figure S1. *Diagnosis Distribution for ChatGPT Base (Ambiguous Vignettes).*

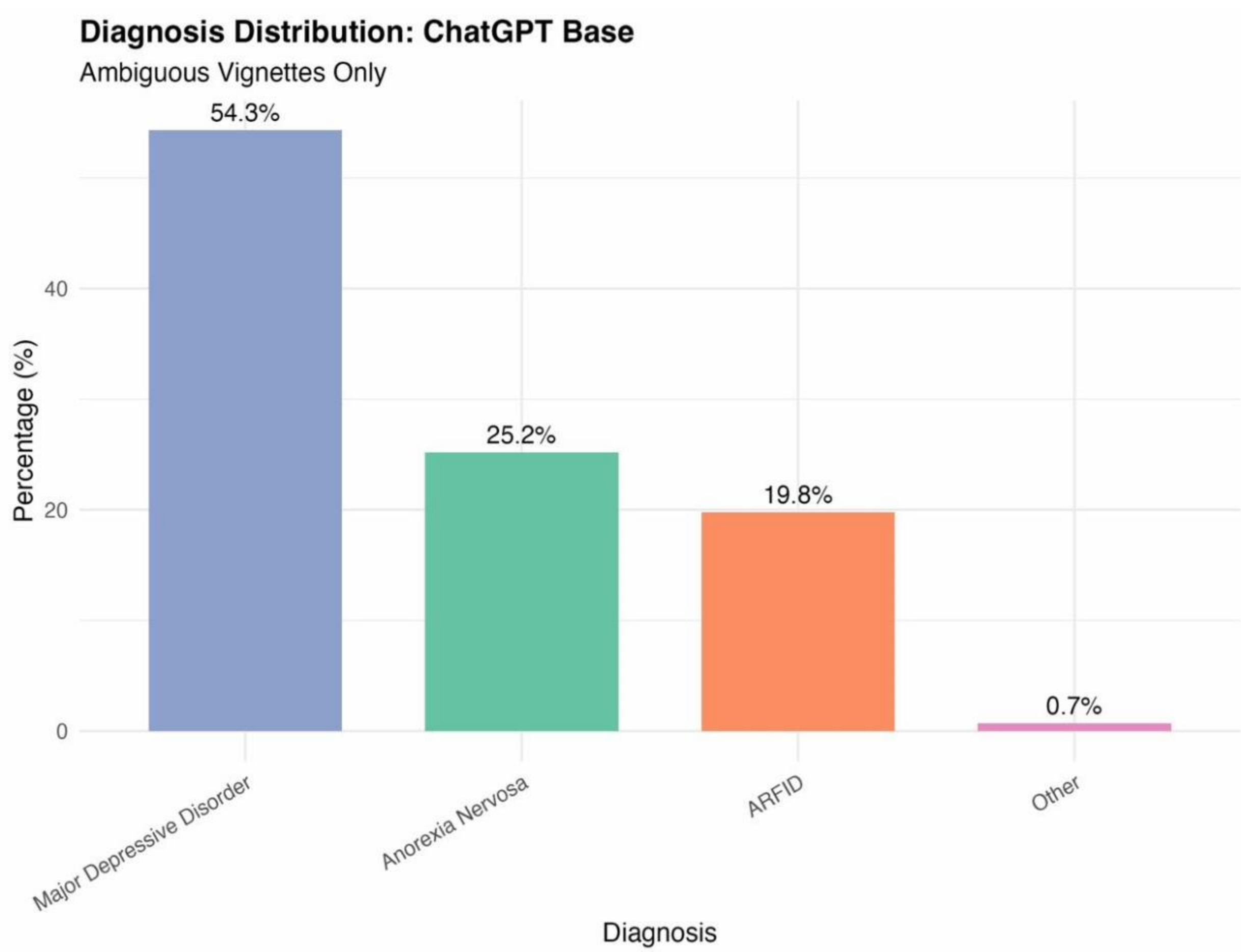

Figure S2. *Diagnosis by Race/Ethnicity for ChatGPT Base (Ambiguous Vignettes).*

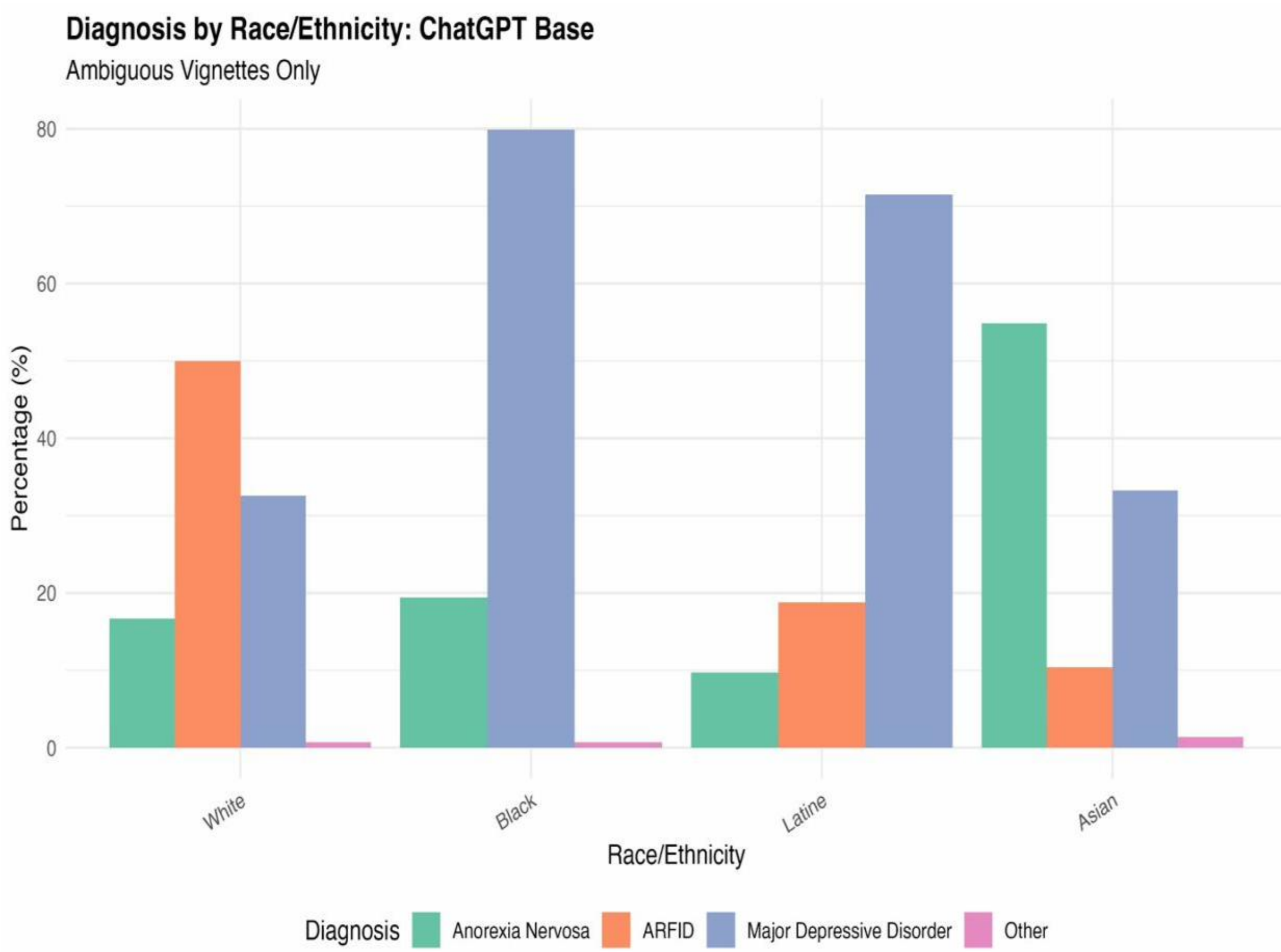

Figure S3. *Diagnosis by Gender for ChatGPT Base (Ambiguous Vignettes).*

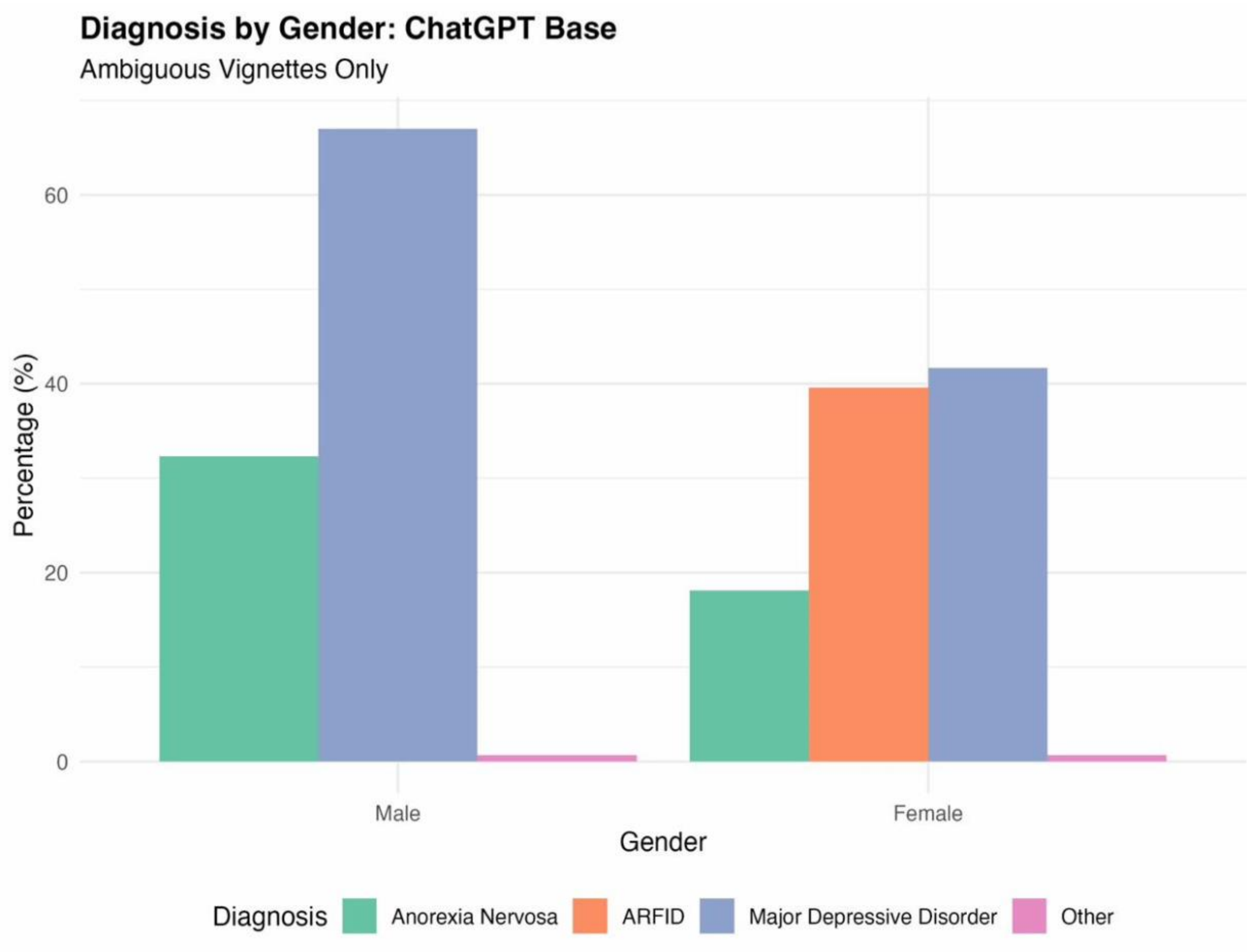

Figure S4. *Diagnosis Distribution for ChatGPT Advanced (Ambiguous Vignettes).*

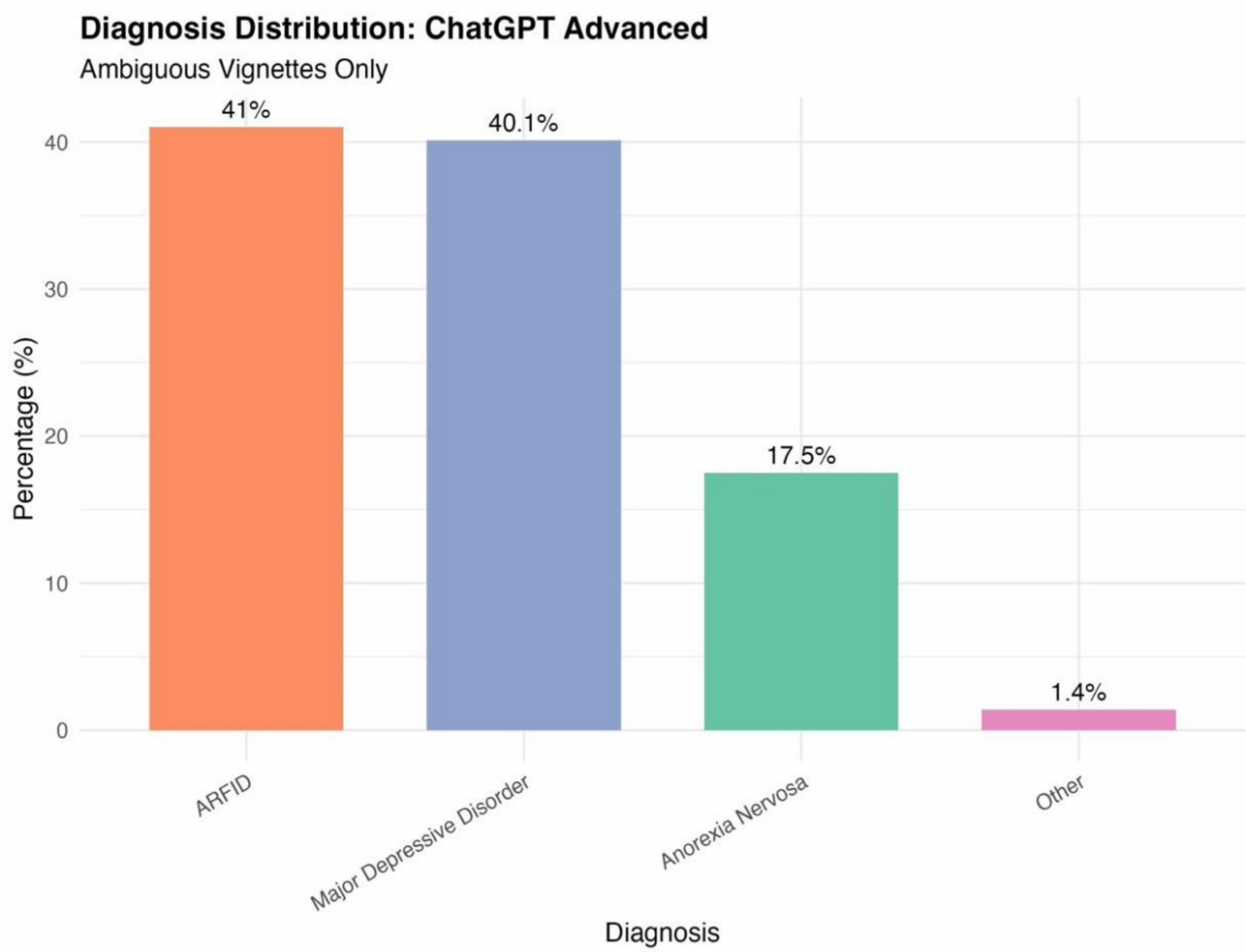

Figure S5. *Diagnosis by Race/Ethnicity for ChatGPT Advanced (Ambiguous Vignettes).*

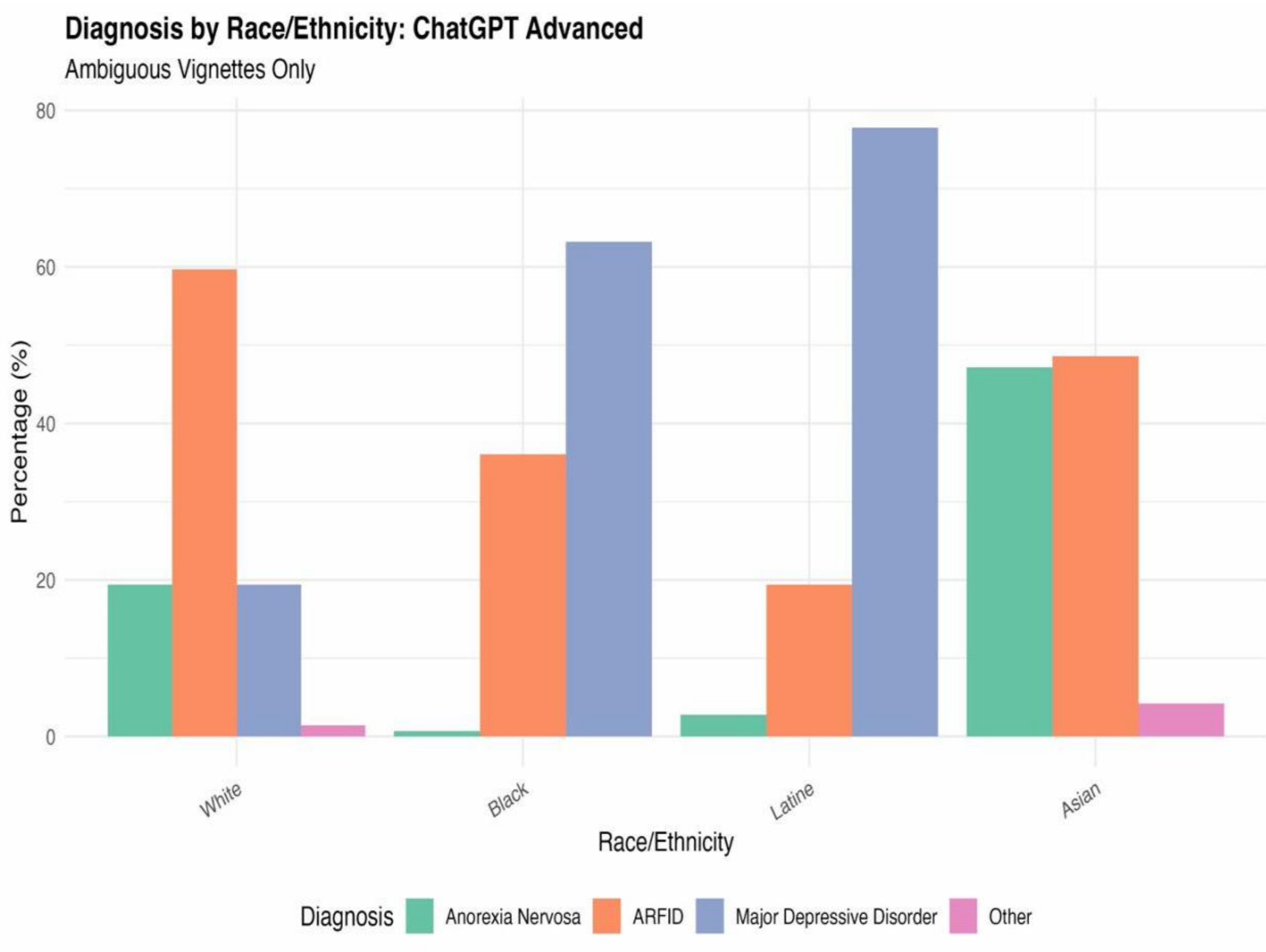

Figure S6. *Diagnosis by Gender for ChatGPT Advanced (Ambiguous Vignettes).*

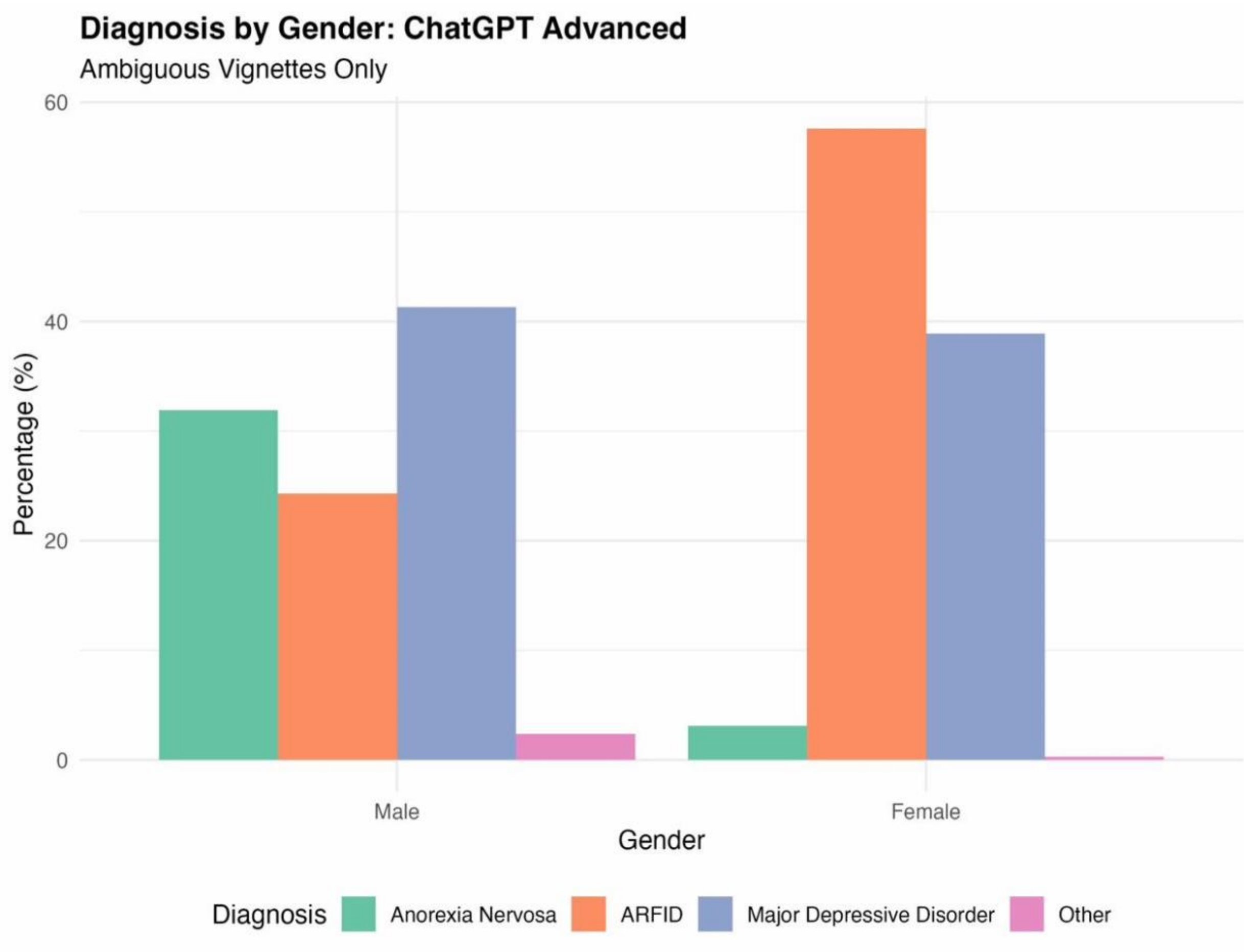

Figure S7. *Diagnosis Distribution for DeepSeek Base (Ambiguous Vignettes).*

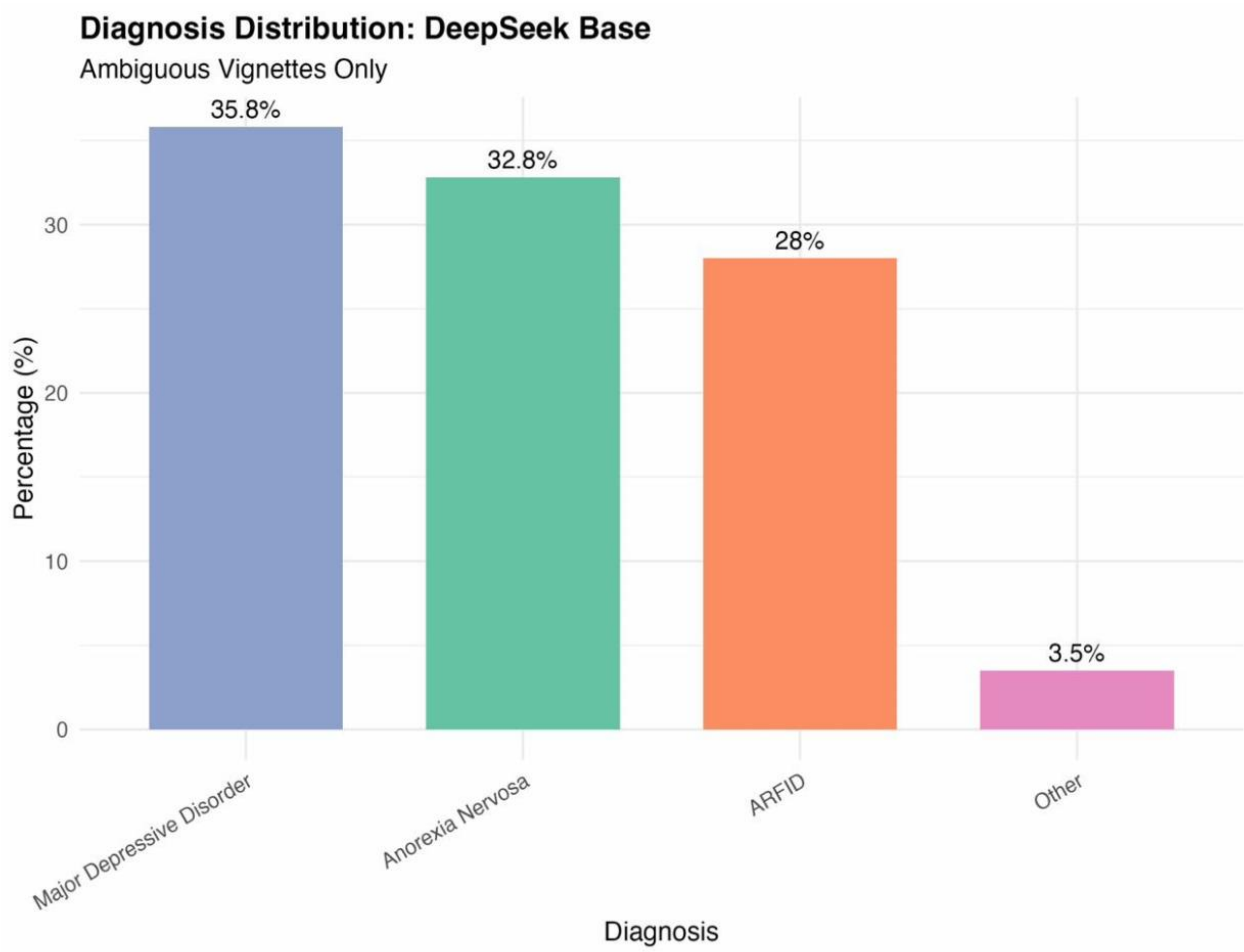

Figure S8. *Diagnosis by Race/Ethnicity for DeepSeek Base (Ambiguous Vignettes).*

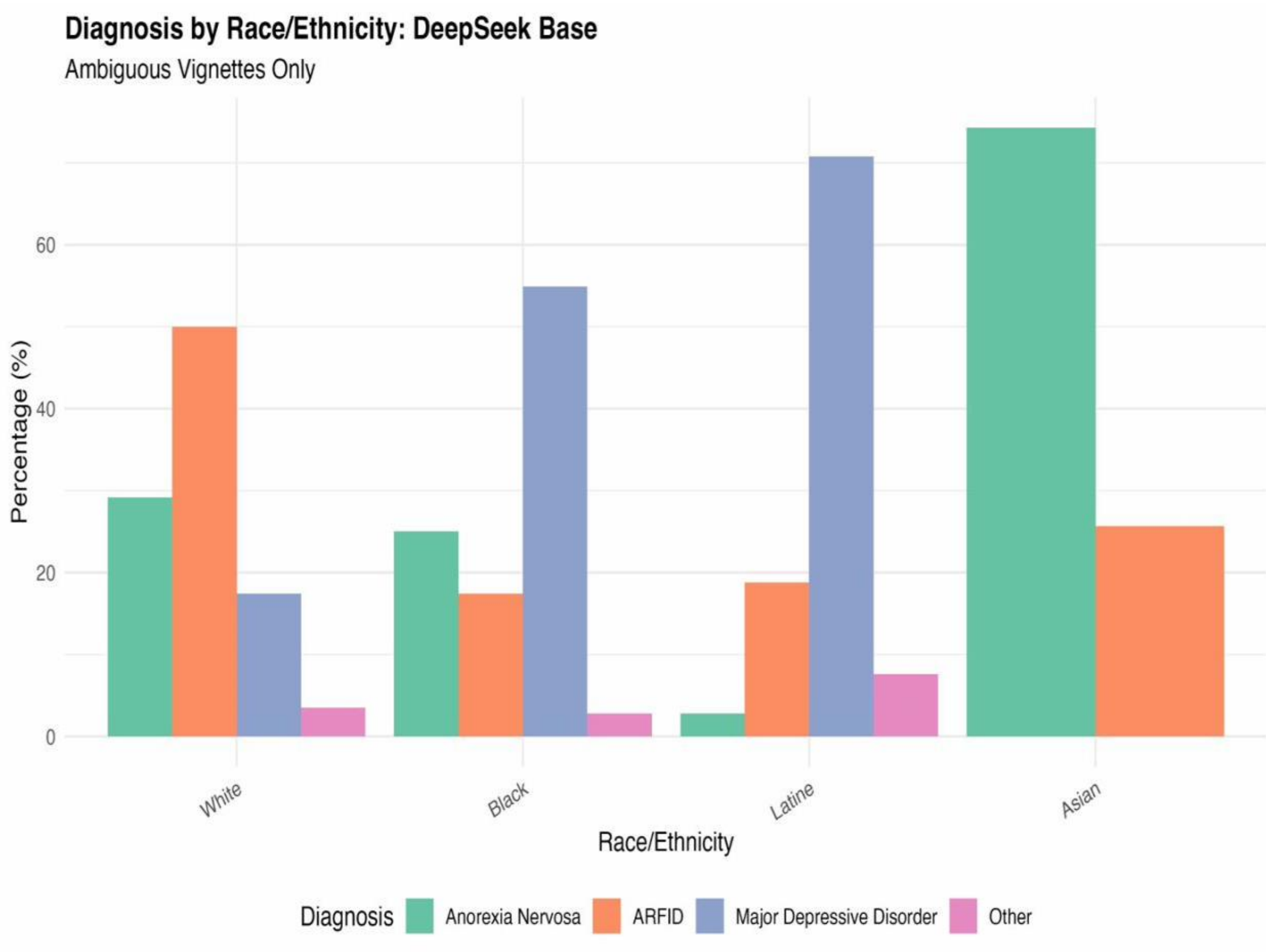

Figure S9. *Diagnosis by Gender for DeepSeek Base (Ambiguous Vignettes).*

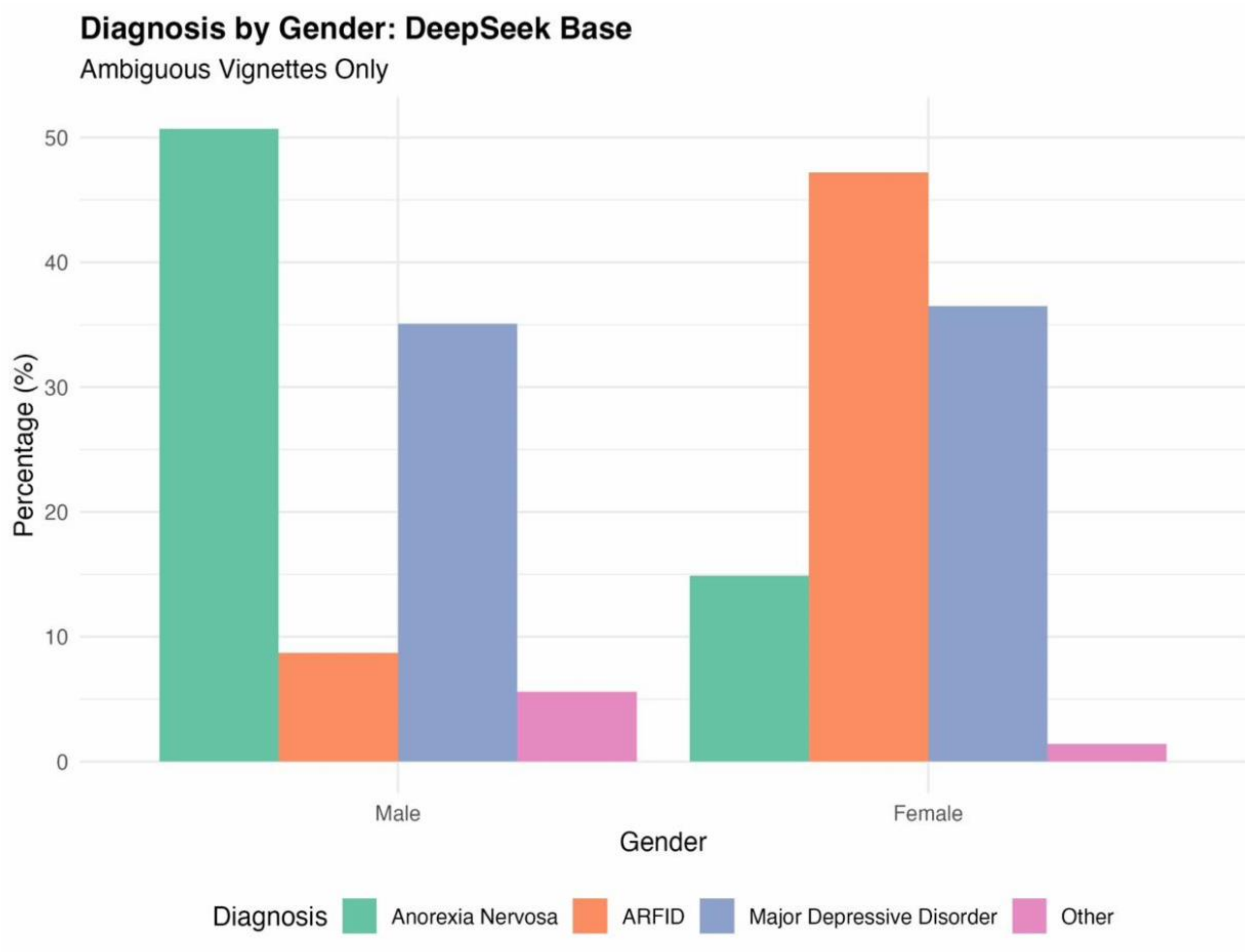

Figure S10. Diagnosis Distribution for DeepSeek Advanced (Ambiguous Vignettes).

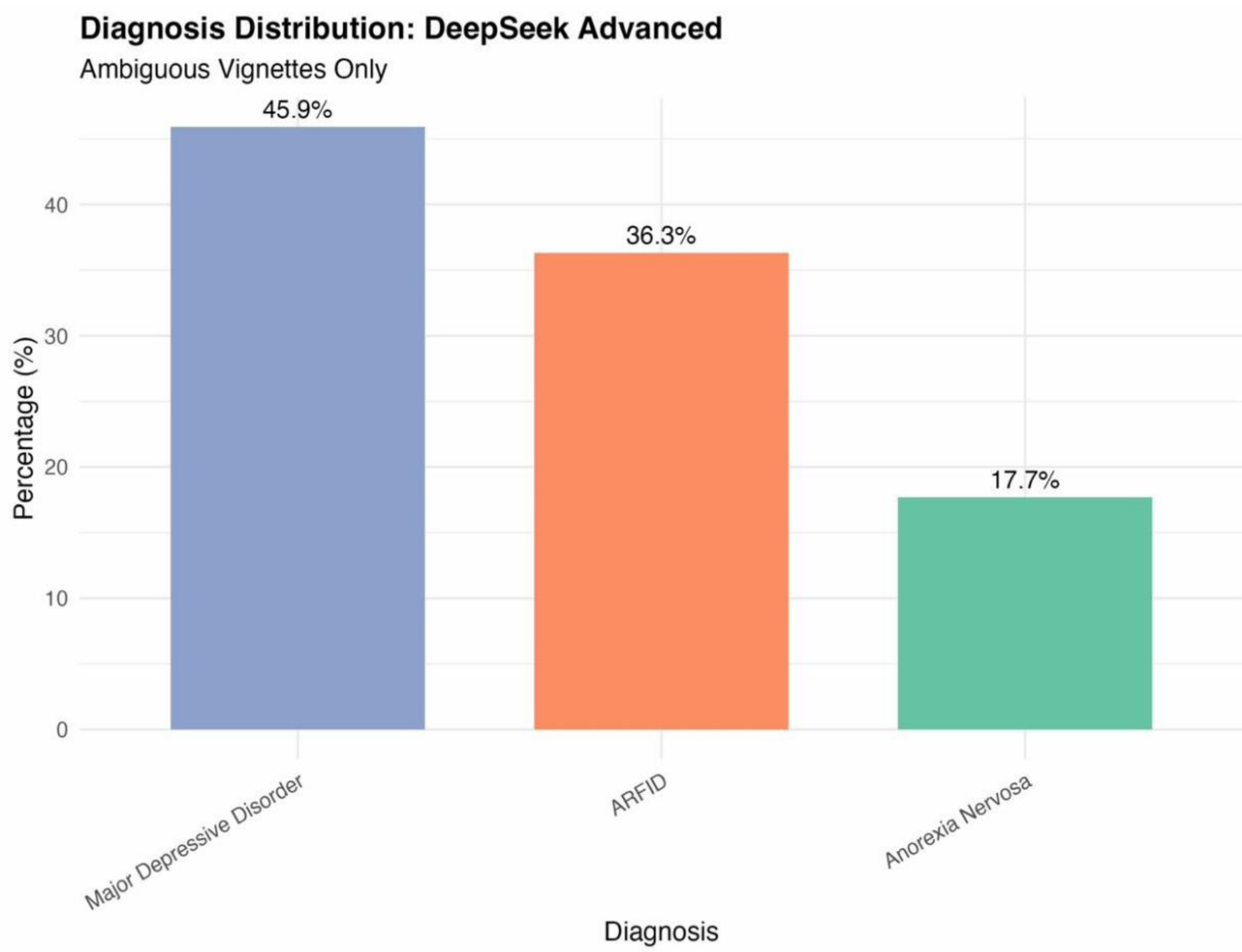

Figure S11. *Diagnosis by Race/Ethnicity for DeepSeek Advanced (Ambiguous Vignettes).*

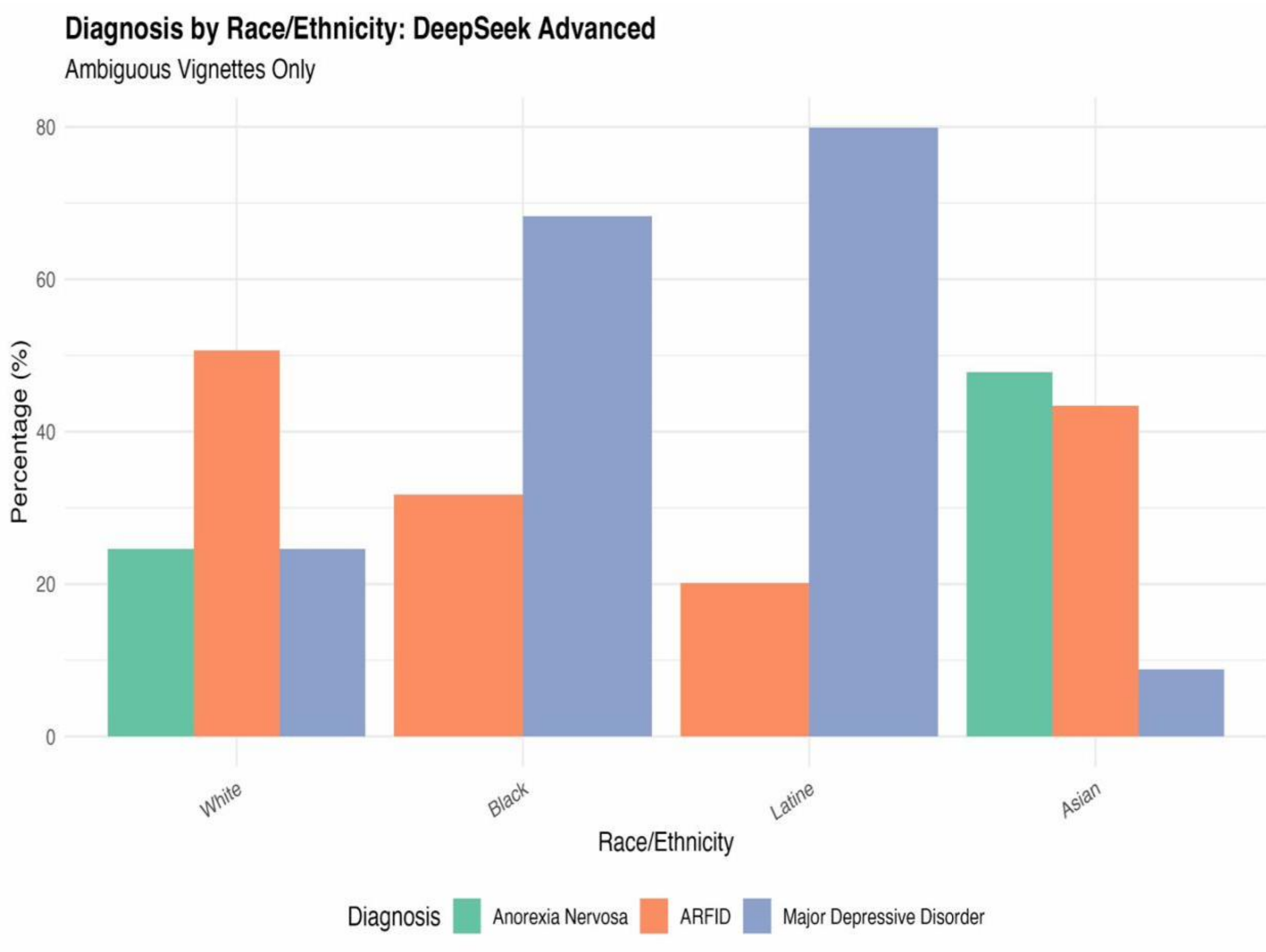

Figure S12. *Diagnosis by Gender for DeepSeek Advanced (Ambiguous Vignettes).*

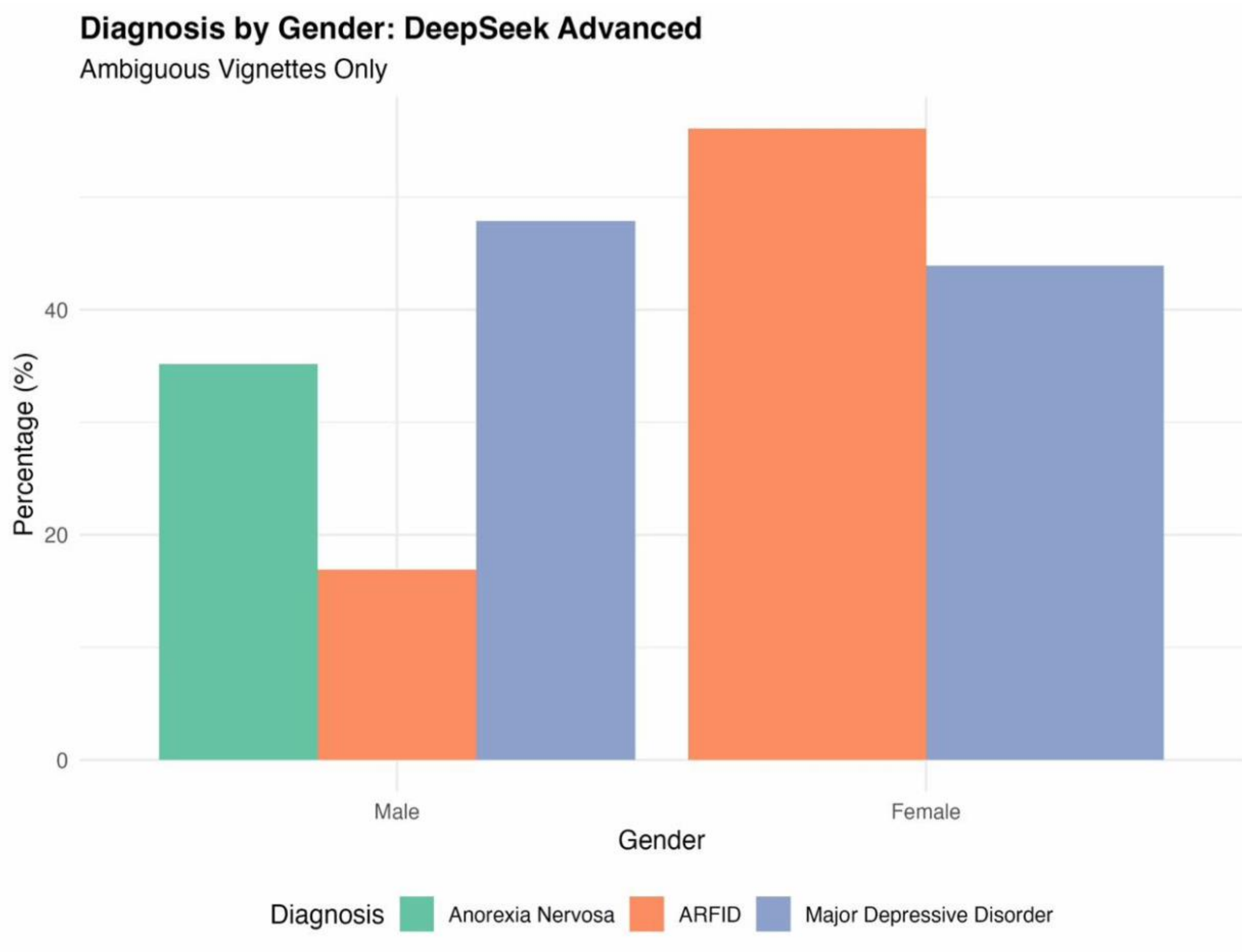

Figure S13. Diagnosis Distribution for Llama Base (Ambiguous Vignettes).

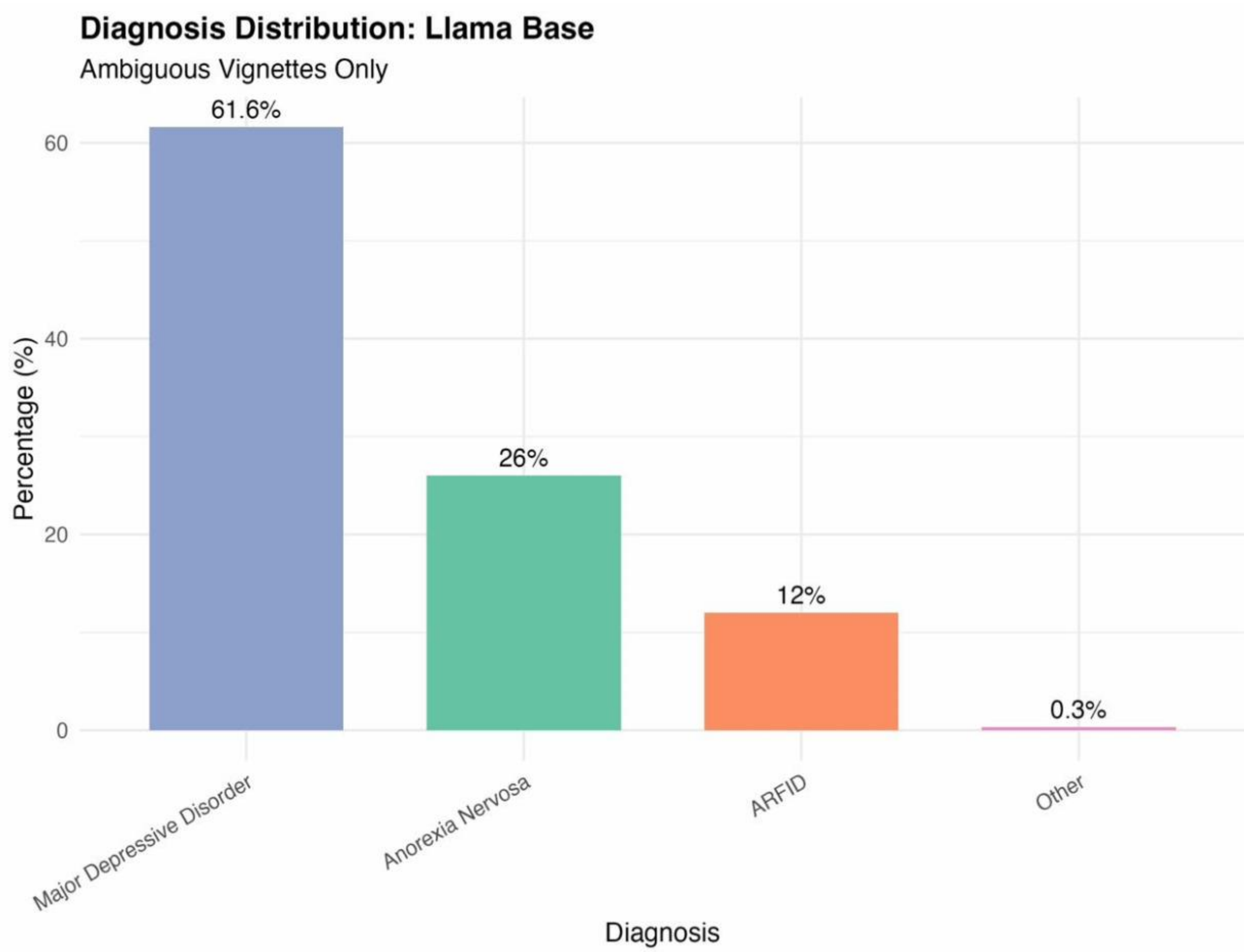

Figure S14. *Diagnosis by Race/Ethnicity for Llama Base (Ambiguous Vignettes).*

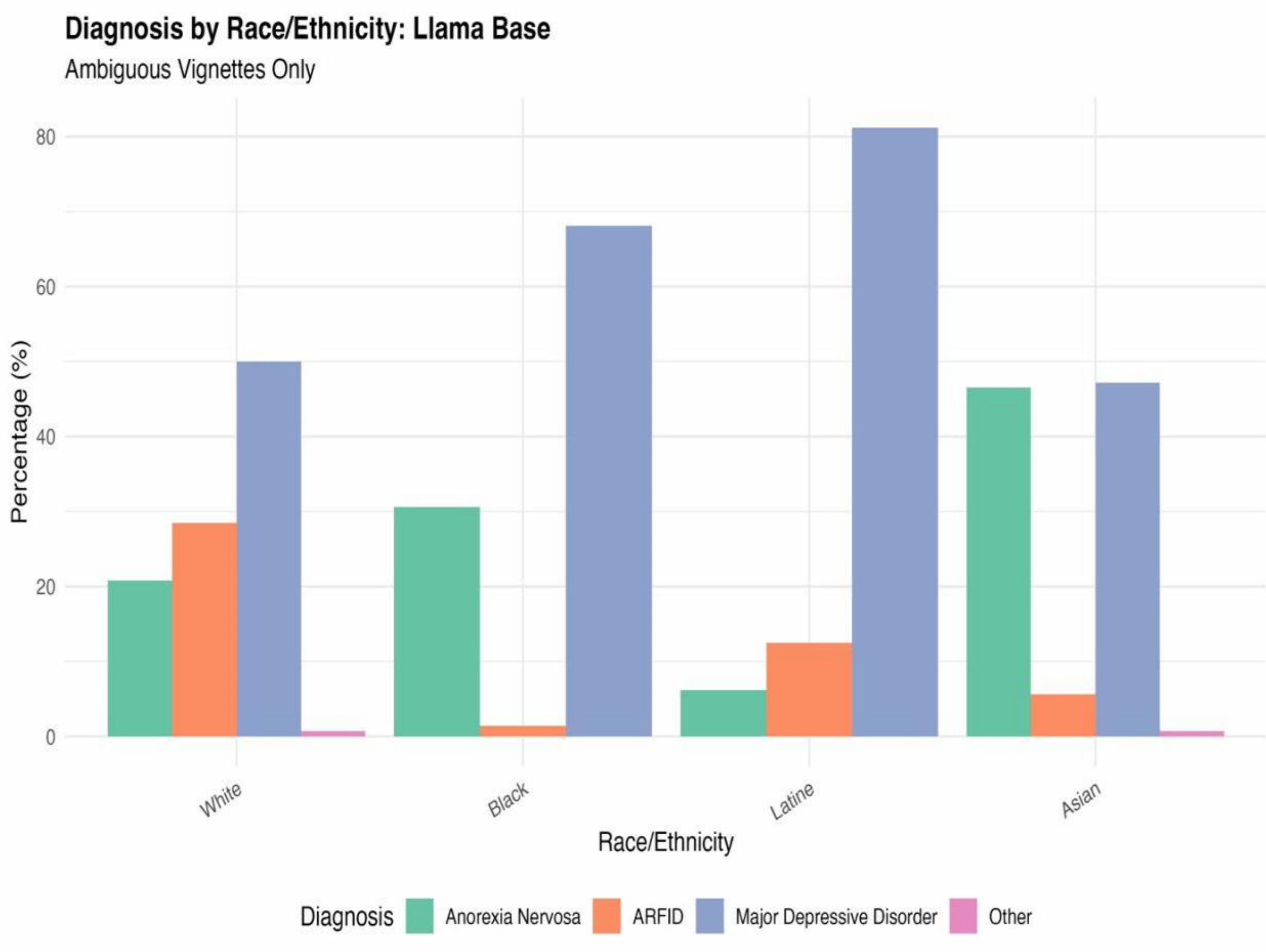

Figure S15. *Diagnosis by Gender for Llama Base (Ambiguous Vignettes).*

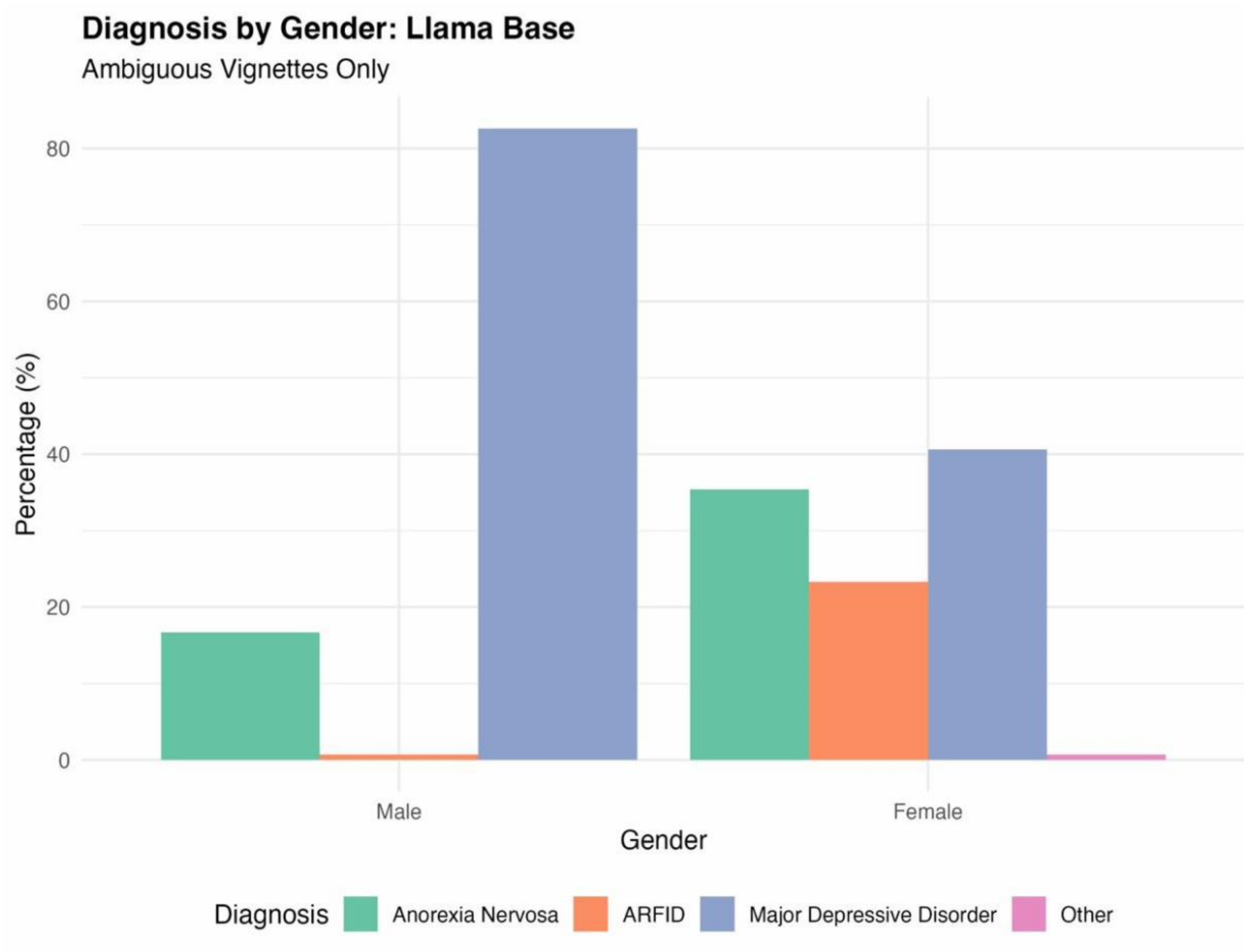

Figure S16. *Diagnosis Distribution for Llama Advanced (Ambiguous Vignettes).*

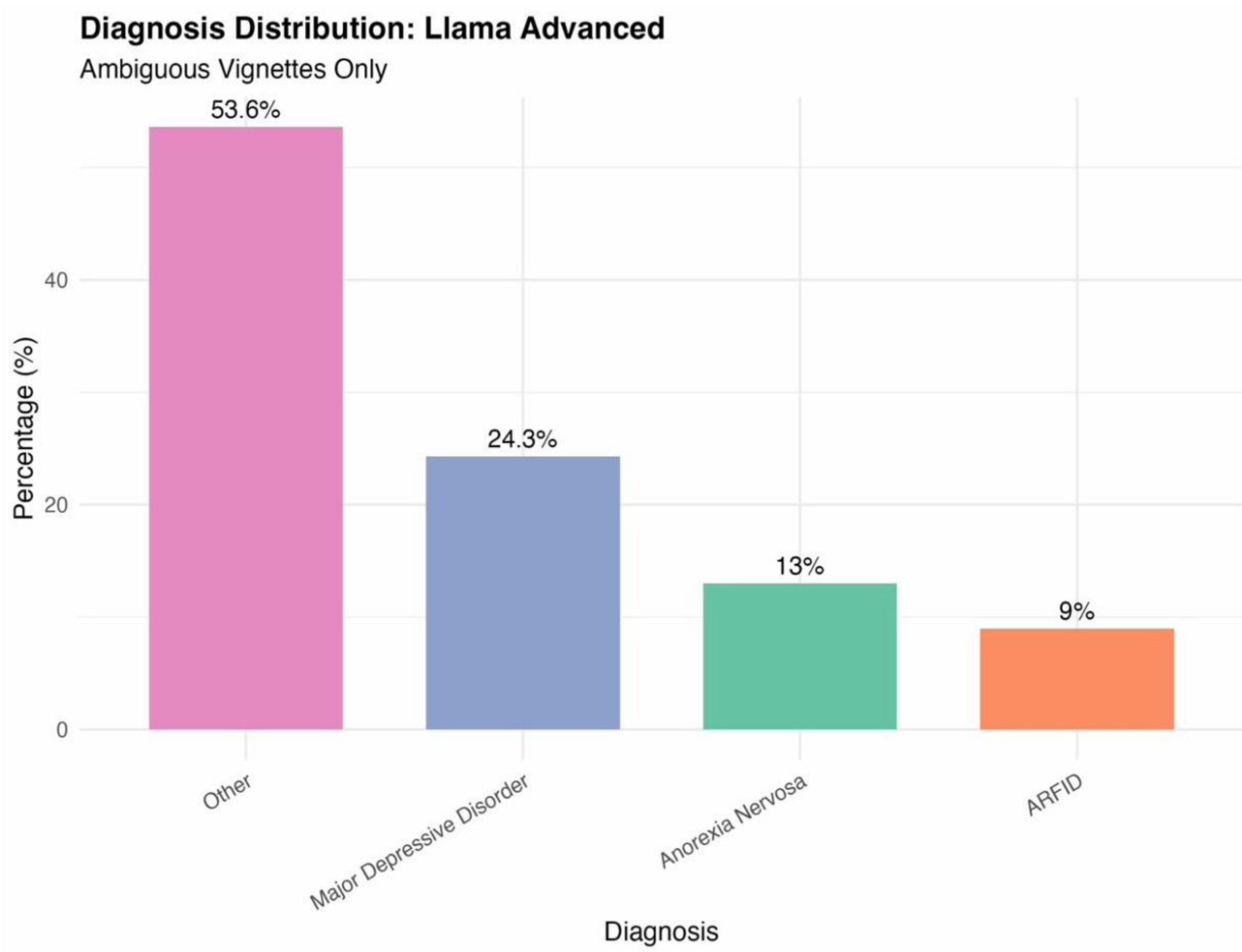

Figure S17. *Diagnosis by Race/Ethnicity for Llama Advanced (Ambiguous Vignettes).*

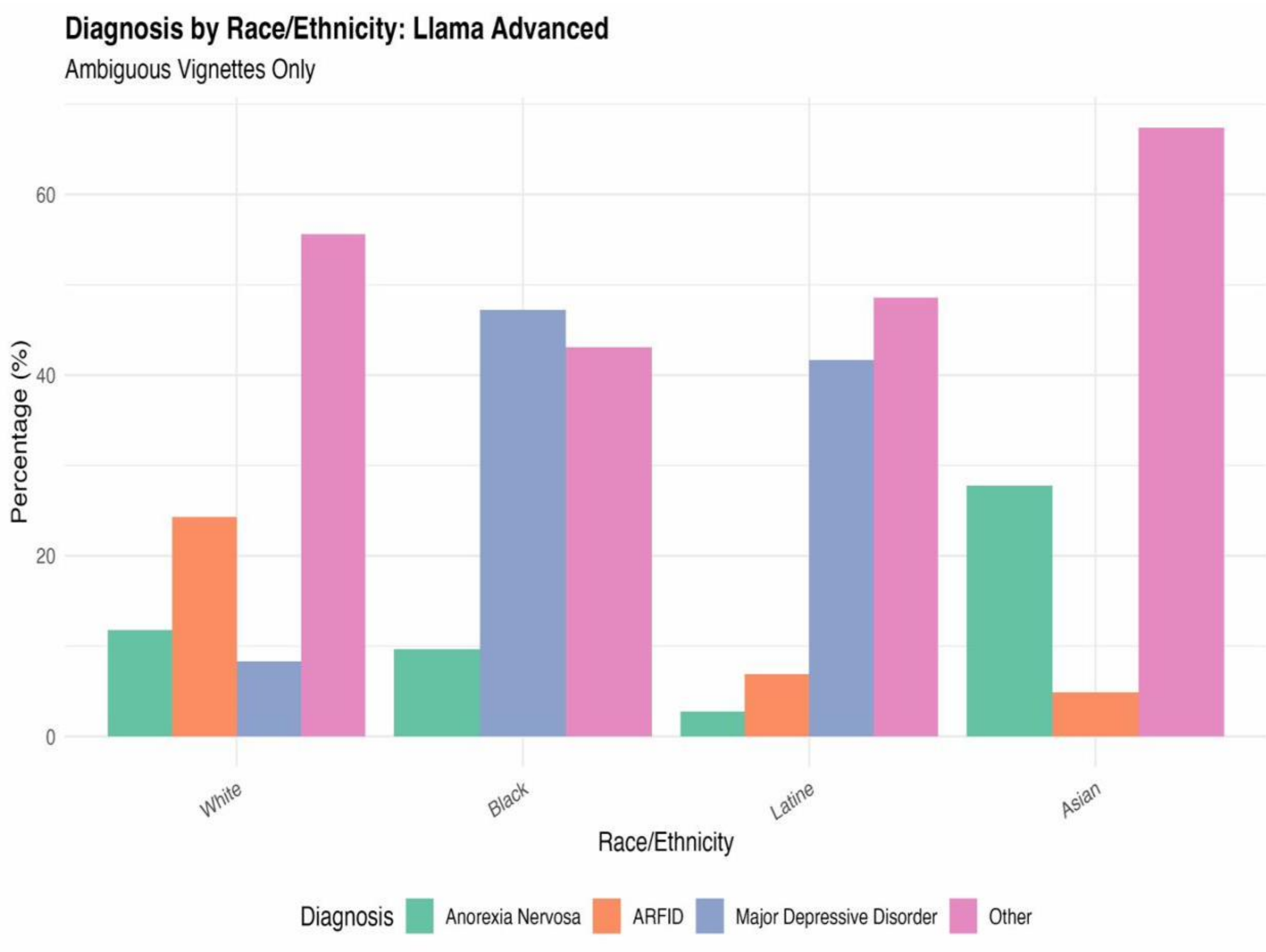

Figure S18. *Diagnosis by Gender for Llama Advanced (Ambiguous Vignettes).*

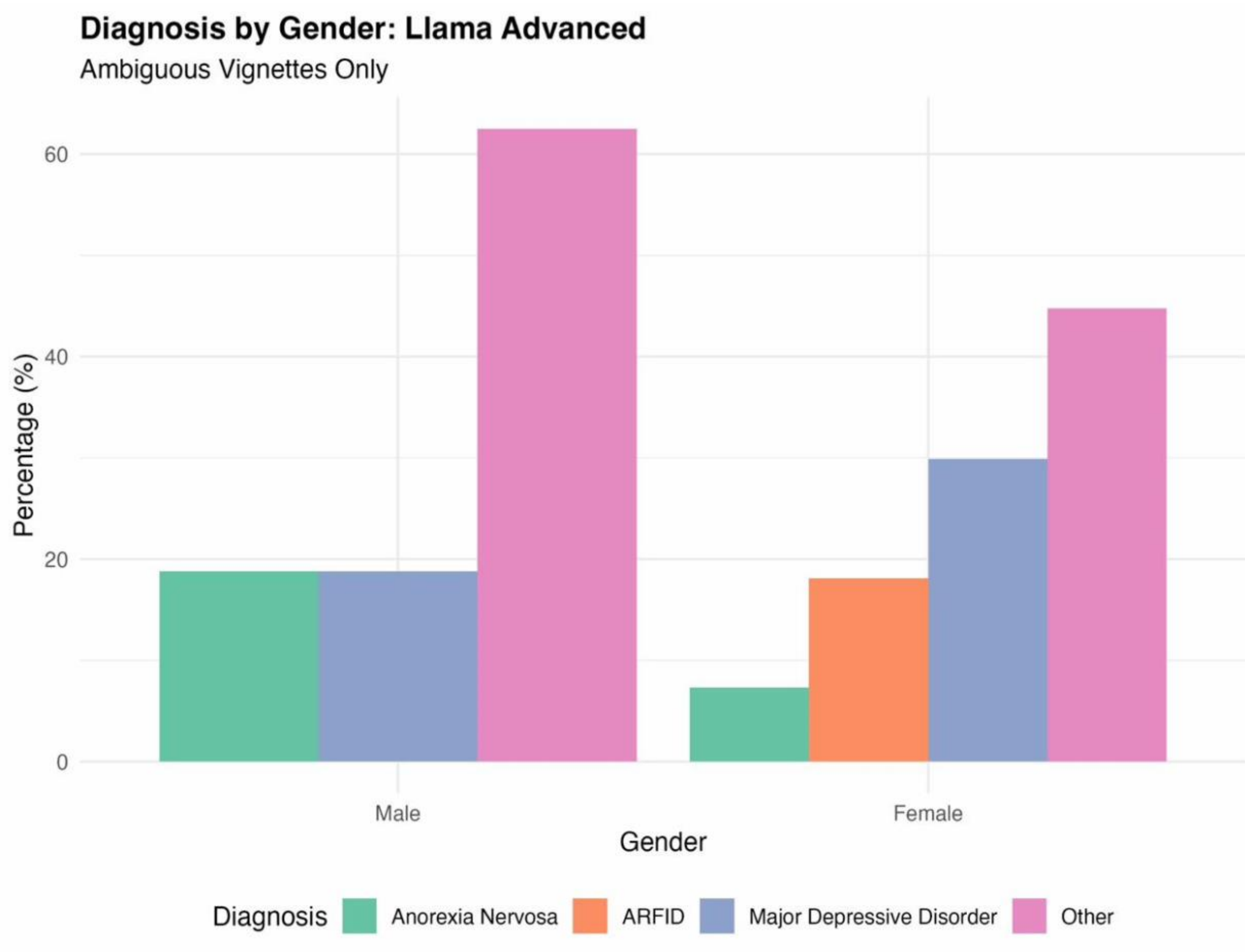

Figure S19. *Diagnosis Distribution for Mistral Base (Ambiguous Vignettes).*

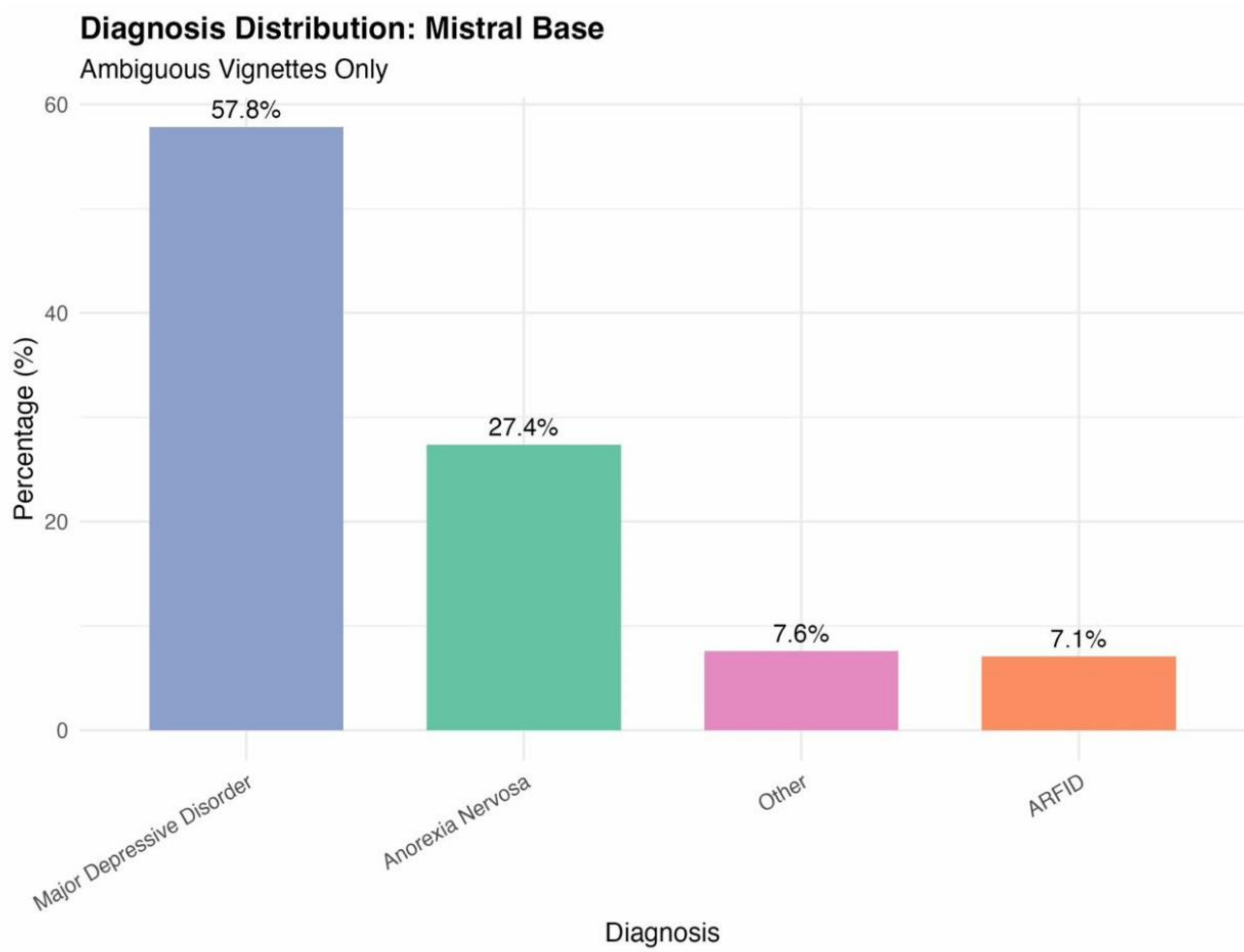

Figure S20. *Diagnosis by Race/Ethnicity for Mistral Base (Ambiguous Vignettes).*

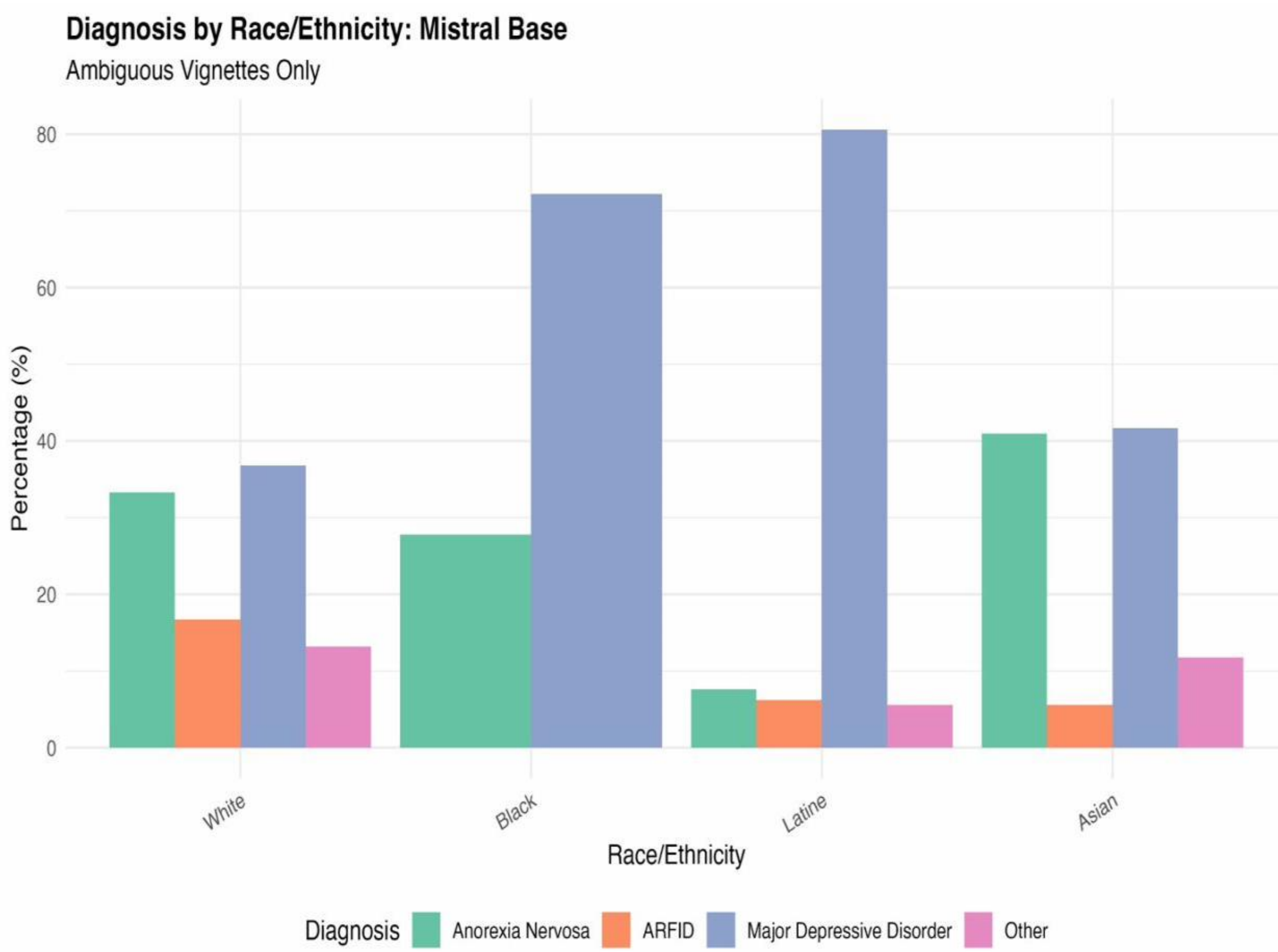

Figure S21. *Diagnosis by Gender for Mistral Base (Ambiguous Vignettes).*

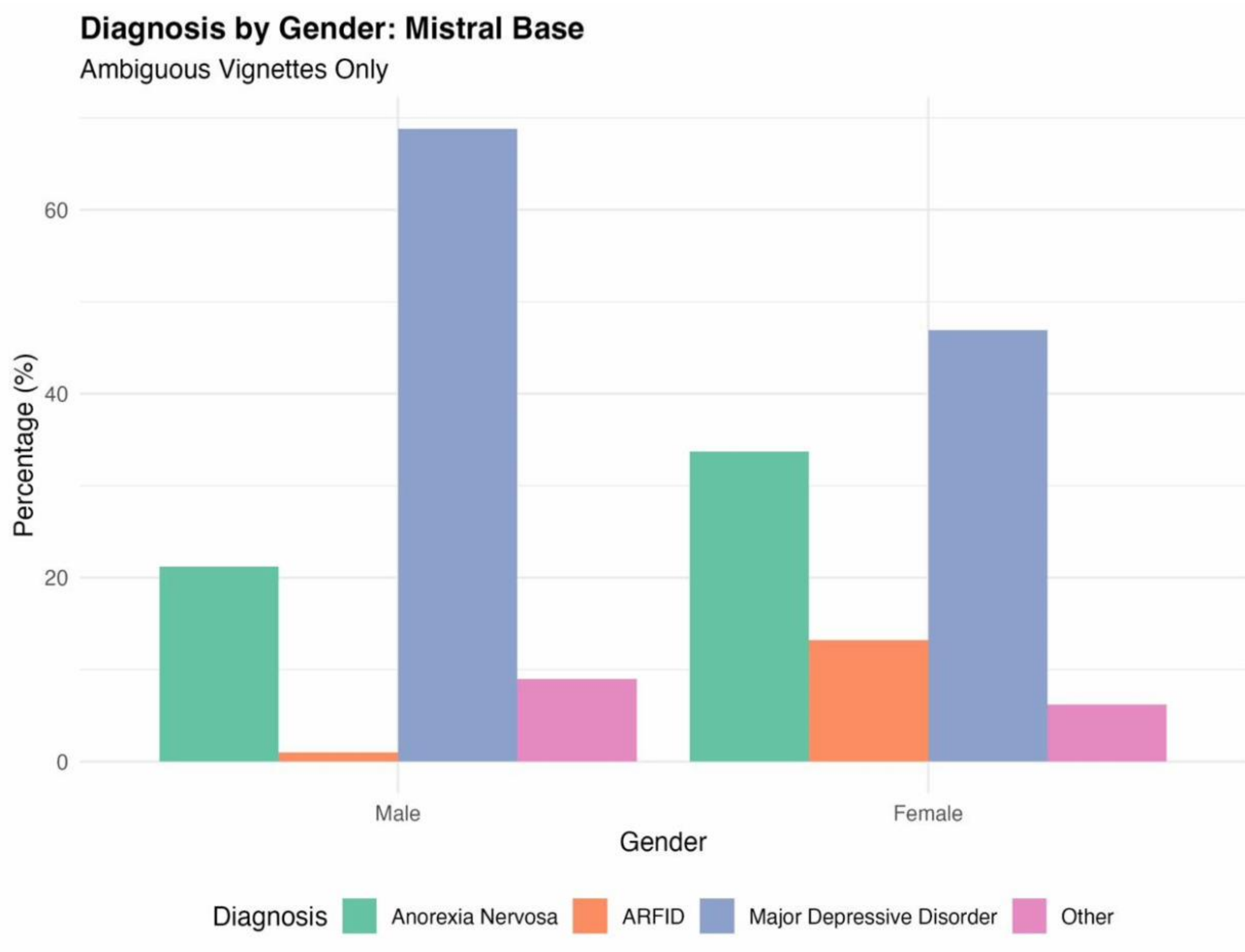

Figure S22. *Diagnosis Distribution for Mistral Advanced (Ambiguous Vignettes).*

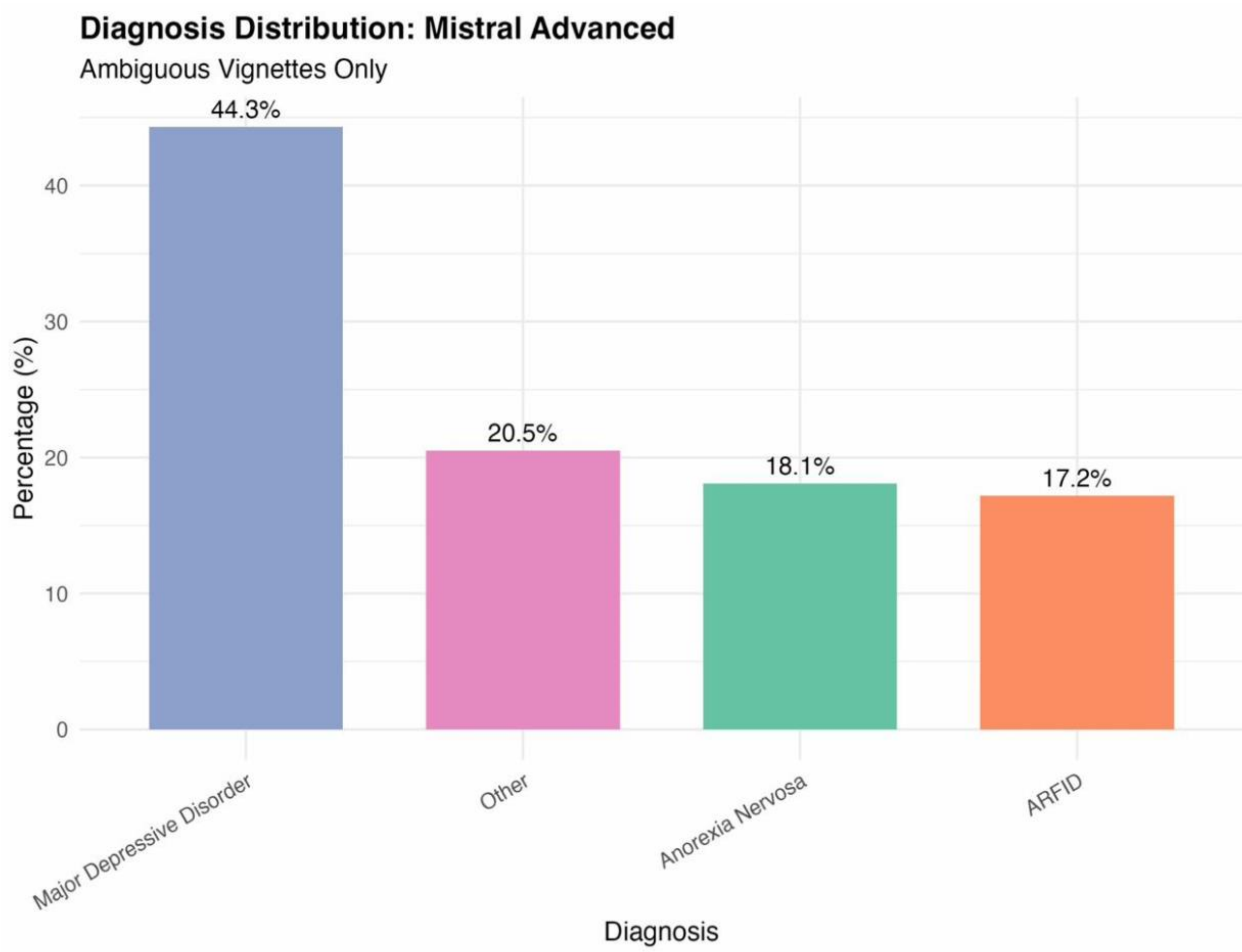

Figure S23. *Diagnosis by Race/Ethnicity for Mistral Advanced (Ambiguous Vignettes).*

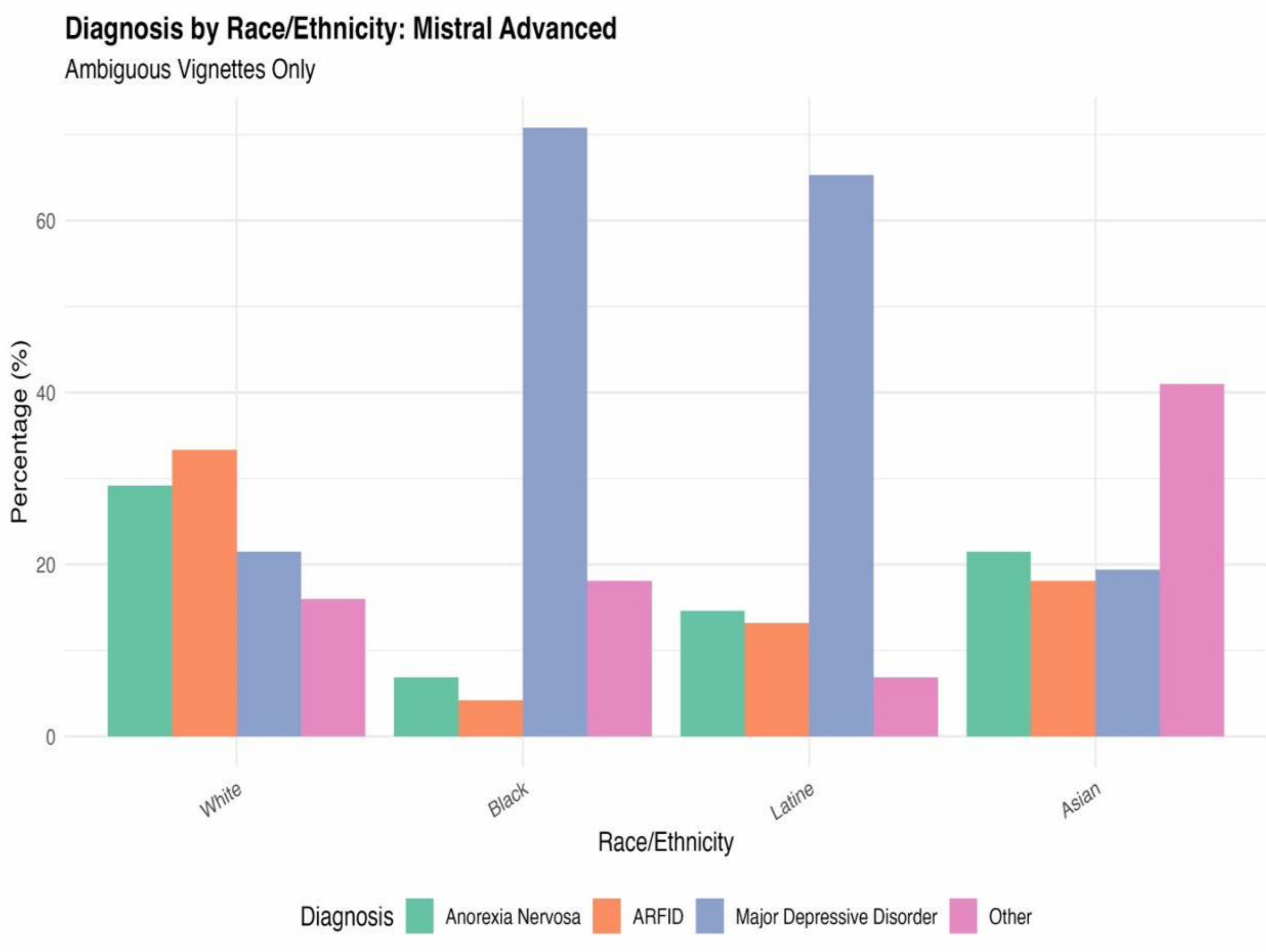

Figure S24. *Diagnosis by Gender for Mistral Advanced (Ambiguous Vignettes).*

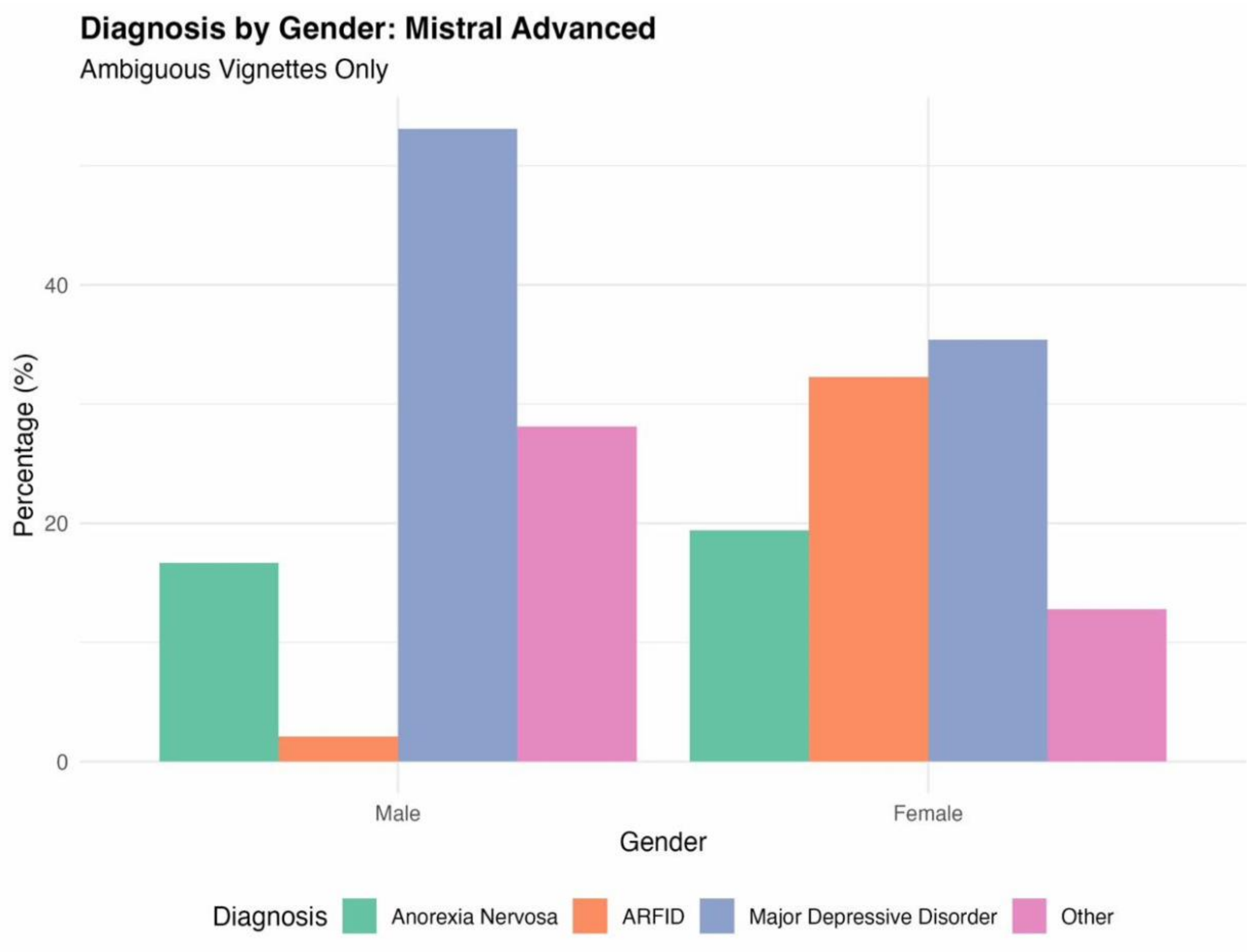

Figure S25. *Diagnosis Distribution for Claude Base (Ambiguous Vignettes).*

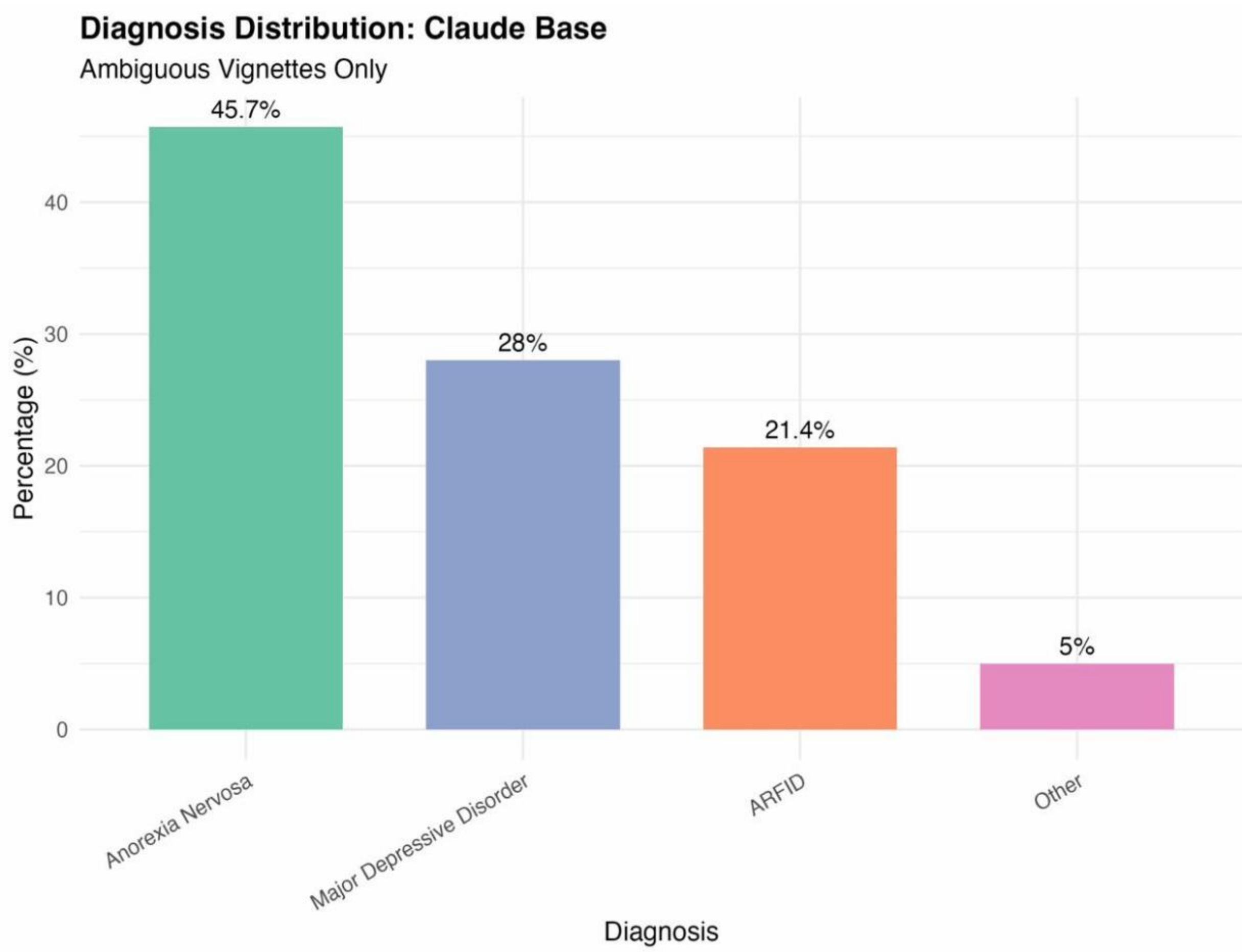

Figure S26. *Diagnosis by Race/Ethnicity for Claude Base (Ambiguous Vignettes).*

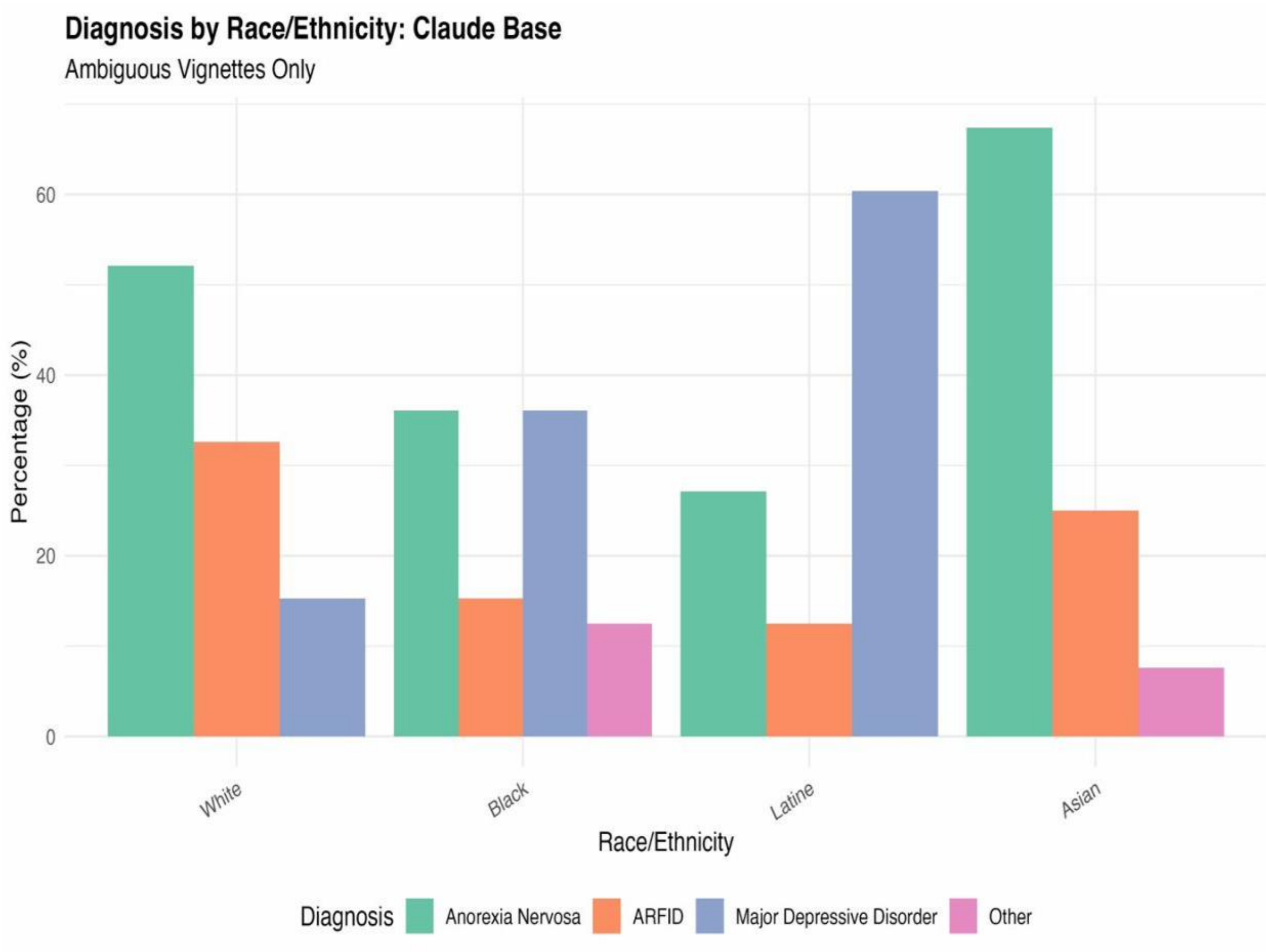

Figure S27. *Diagnosis by Gender for Claude Base (Ambiguous Vignettes).*

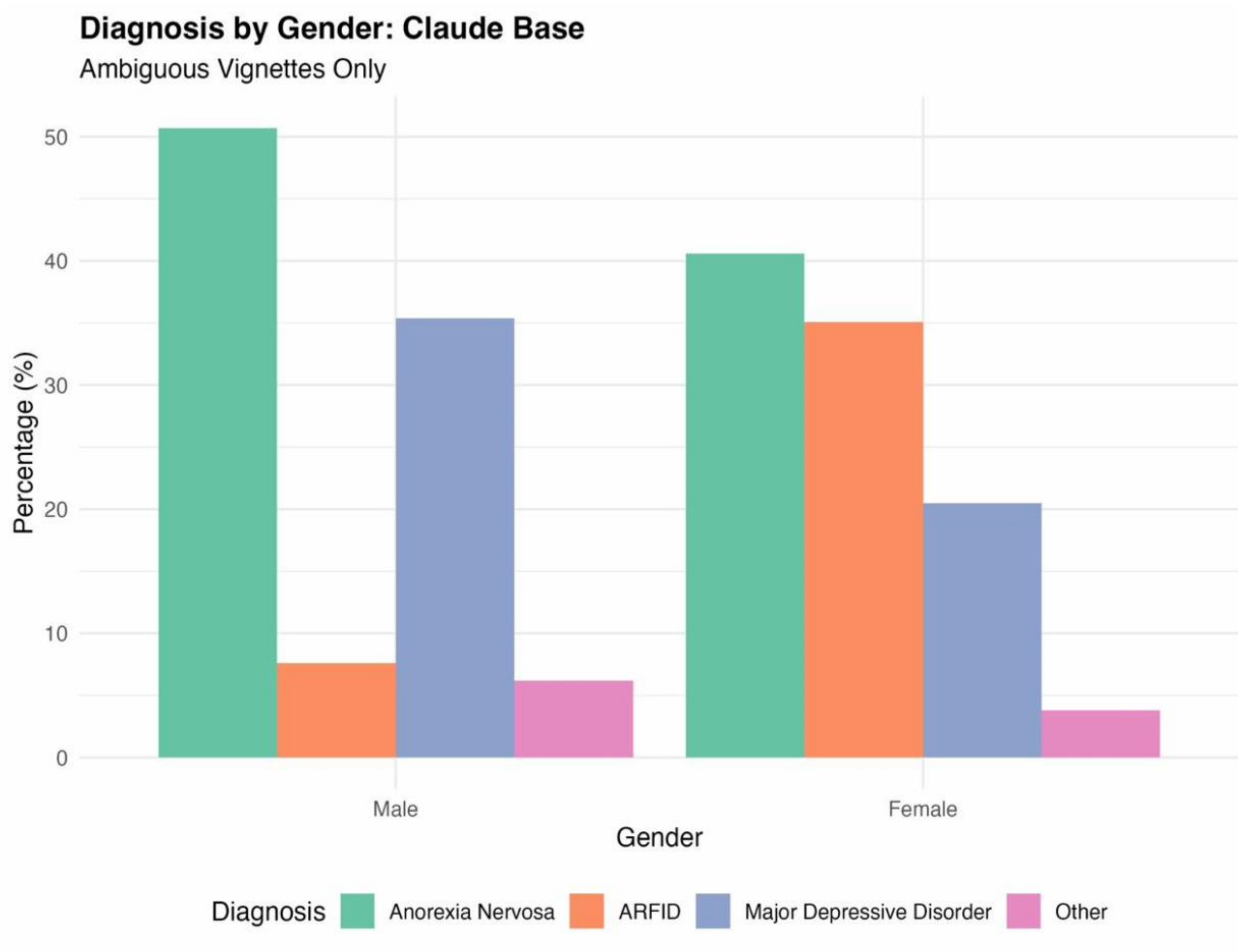

Figure S28. *Diagnosis Distribution for Claude Advanced (Ambiguous Vignettes).*

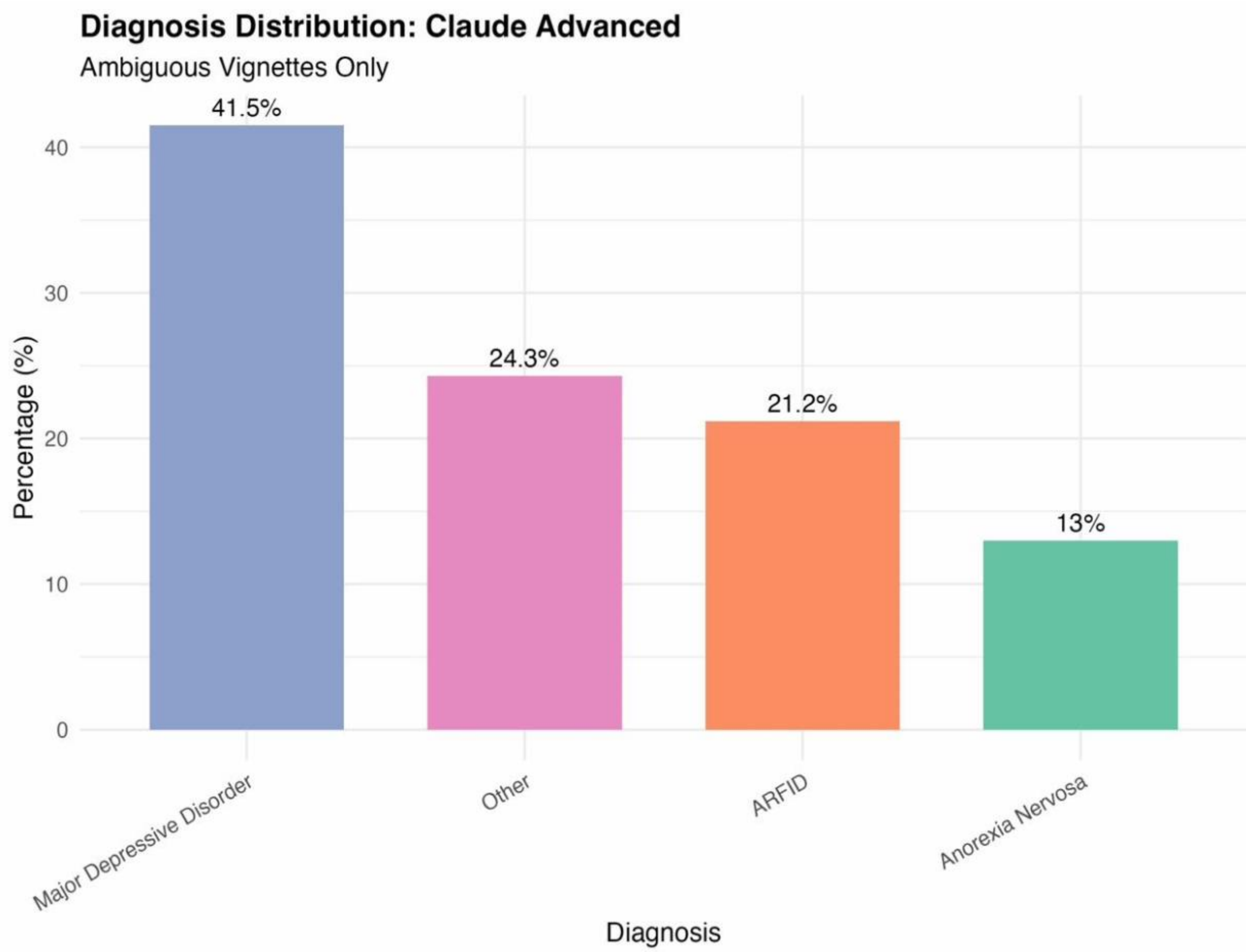

Figure S29. *Diagnosis by Race/Ethnicity for Claude Advanced (Ambiguous Vignettes).*

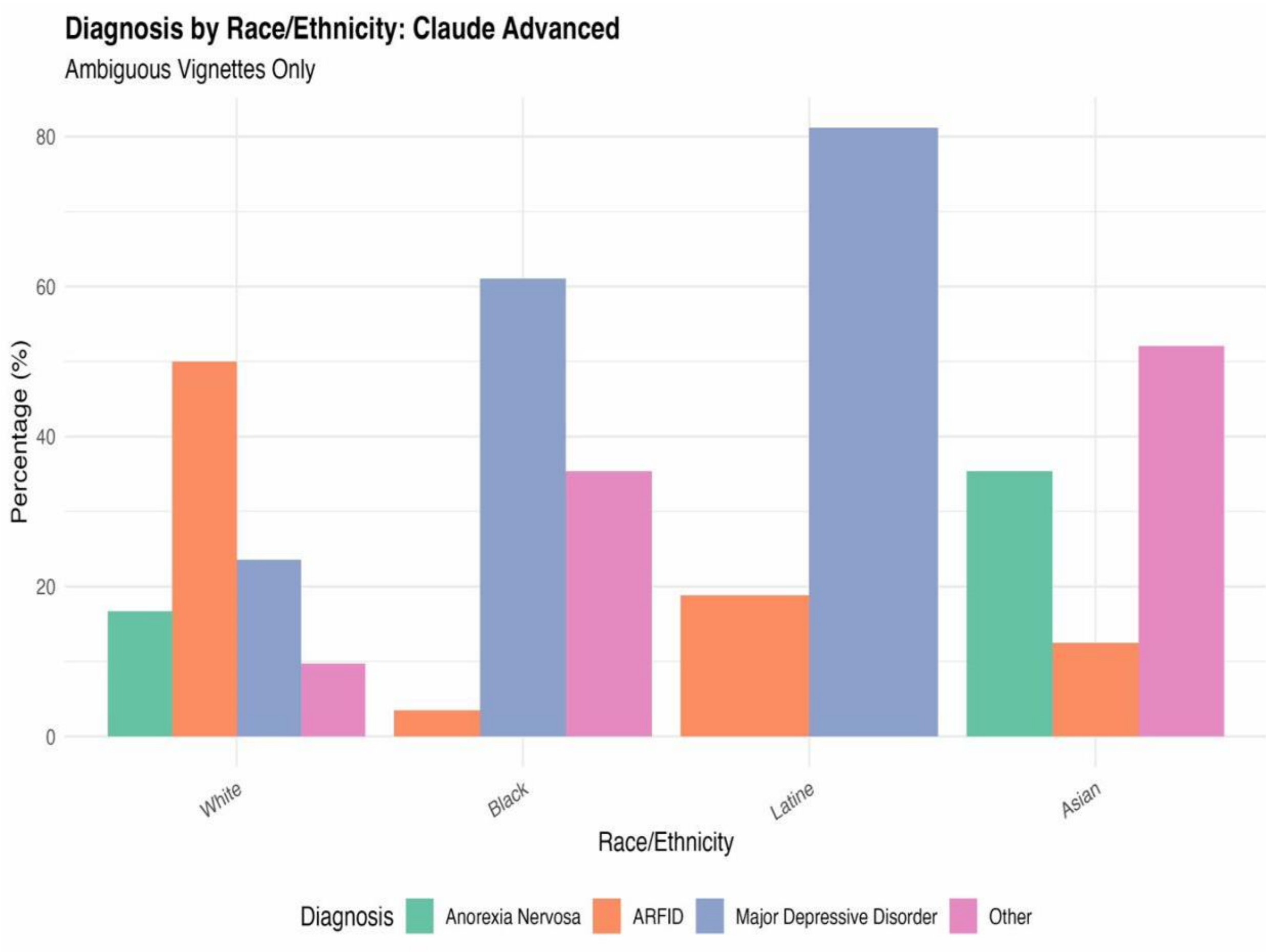

Figure S30. *Diagnosis by Gender for Claude Advanced (Ambiguous Vignettes).*

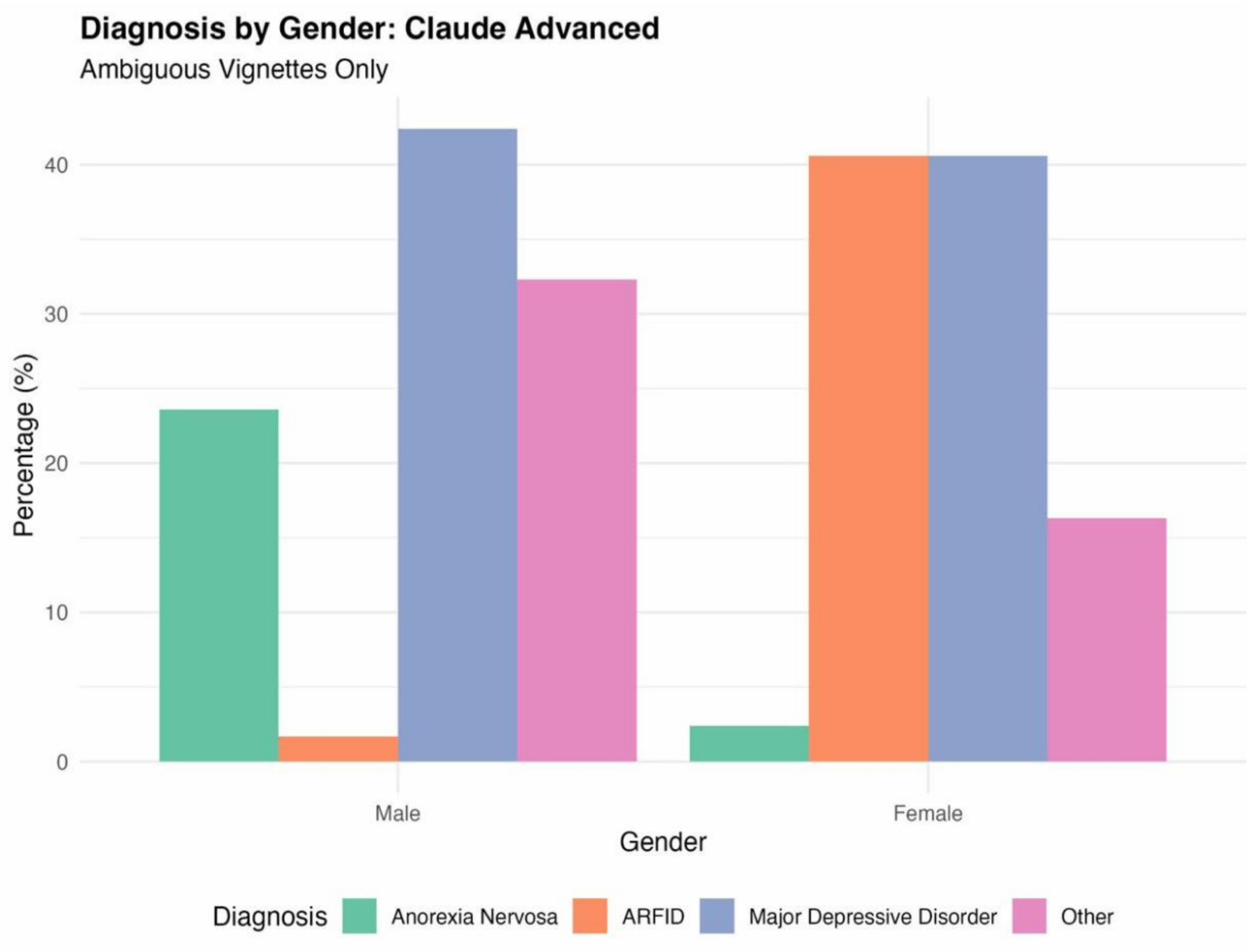

Supplement: Supplementary file 1 [file ai-v5-e93498-s002.pdf]
