# Supplementary material for: Implicit Bias in Large Language Model Diagnosis of Eating Disorders: Experimental Vignette Study
Source: JMIR AI. 2026 Sep 23;5:e93498. doi: 10.2196/93498 (PMC13600661; doi:10.2196/93498)
Supplement: Supplementary file 2 [file ai-v5-e93498-s001.docx]

**Multimedia Appendix 1**

Supplementary Tables S1–S4

**Table S1.** Per-Model Logistic Regression Odds Ratios for Demographic Predictors of Anorexia Nervosa Diagnosis (Ambiguous Vignettes).

| **LLM** | **Predictor** | **OR** | **95% CI** | **P** |
| --- | --- | --- | --- | --- |
| ChatGPT Base | Female vs Male | 0.36 | 0.23–0.57 | <.001 |
|  | Black vs White | 1.23 | 0.65–2.32 | 0.519 |
|  | Latine vs White | 0.51 | 0.24–1.06 | 0.072 |
|  | Asian vs White | 8.00 | 4.39–14.56 | <.001 |
|  | Young Adult vs Adolescent | 0.74 | 0.44–1.23 | 0.245 |
|  | Adult vs Adolescent | 0.33 | 0.19–0.58 | <.001 |
|  | Low SES vs High SES | 0.38 | 0.24–0.60 | <.001 |
|  | Limited vs Fluent English | 0.59 | 0.38–0.92 | 0.021 |
|  | Non-Urban vs Urban | 1.25 | 0.81–1.94 | 0.317 |
| ChatGPT Advanced | Female vs Male | 0.02 | 0.01–0.05 | <.001 |
|  | Black vs White | 0.02 | 0.00–0.15 | <.001 |
|  | Latine vs White | 0.08 | 0.03–0.26 | <.001 |
|  | Asian vs White | 10.12 | 4.64–22.07 | <.001 |
|  | Young Adult vs Adolescent | 1.51 | 0.68–3.35 | 0.313 |
|  | Adult vs Adolescent | 1.63 | 0.73–3.62 | 0.229 |
|  | Low SES vs High SES | 0.32 | 0.16–0.63 | 0.001 |
|  | Limited vs Fluent English | 0.62 | 0.32–1.18 | 0.144 |
|  | Non-Urban vs Urban | 0.76 | 0.40–1.46 | 0.414 |
| DeepSeek Base | Female vs Male | 0.03 | 0.01–0.07 | <.001 |
|  | Black vs White | 0.73 | 0.38–1.38 | 0.328 |
|  | Latine vs White | 0.04 | 0.01–0.12 | <.001 |
|  | Asian vs White | 29.31 | 12.46–68.99 | <.001 |
|  | Young Adult vs Adolescent | 4.02 | 2.11–7.67 | <.001 |
|  | Adult vs Adolescent | 2.74 | 1.45–5.18 | 0.002 |
|  | Low SES vs High SES | 0.53 | 0.32–0.89 | 0.015 |
|  | Limited vs Fluent English | 0.79 | 0.48–1.31 | 0.366 |
|  | Non-Urban vs Urban | 1.11 | 0.67–1.84 | 0.698 |
| DeepSeek Advanced | Female vs Male | 0.00 | 0.00–Inf | 0.990 |
|  | Black vs White | 0.00 | 0.00–Inf | 0.994 |
|  | Latine vs White | 0.00 | 0.00–Inf | 0.994 |
|  | Asian vs White | 45.45 | 10.35–199.69 | <.001 |
|  | Young Adult vs Adolescent | 20.11 | 4.74–85.40 | <.001 |
|  | Adult vs Adolescent | 18.81 | 4.41–80.25 | <.001 |
|  | Low SES vs High SES | 0.23 | 0.08–0.68 | 0.007 |
|  | Limited vs Fluent English | 0.34 | 0.12–0.96 | 0.042 |
|  | Non-Urban vs Urban | 1.39 | 0.50–3.83 | 0.525 |
| Llama Base | Female vs Male | 3.18 | 2.08–4.86 | <.001 |
|  | Black vs White | 1.74 | 1.00–3.02 | 0.052 |
|  | Latine vs White | 0.24 | 0.11–0.53 | <.001 |
|  | Asian vs White | 3.65 | 2.13–6.29 | <.001 |
|  | Young Adult vs Adolescent | 1.00 | 0.61–1.64 | 1.000 |
|  | Adult vs Adolescent | 0.74 | 0.45–1.23 | 0.247 |
|  | Low SES vs High SES | 0.80 | 0.53–1.21 | 0.297 |
|  | Limited vs Fluent English | 0.70 | 0.47–1.06 | 0.096 |
|  | Non-Urban vs Urban | 1.09 | 0.72–1.65 | 0.676 |
| Llama Advanced | Female vs Male | 0.26 | 0.14–0.48 | <.001 |
|  | Black vs White | 0.78 | 0.35–1.74 | 0.540 |
|  | Latine vs White | 0.18 | 0.06–0.58 | 0.004 |
|  | Asian vs White | 3.70 | 1.84–7.44 | <.001 |
|  | Young Adult vs Adolescent | 0.54 | 0.29–1.02 | 0.059 |
|  | Adult vs Adolescent | 0.23 | 0.11–0.48 | <.001 |
|  | Low SES vs High SES | 0.15 | 0.08–0.30 | <.001 |
|  | Limited vs Fluent English | 0.75 | 0.43–1.32 | 0.322 |
|  | Non-Urban vs Urban | 0.82 | 0.47–1.43 | 0.479 |
| Mistral Base | Female vs Male | 2.02 | 1.36–2.99 | <.001 |
|  | Black vs White | 0.76 | 0.46–1.27 | 0.297 |
|  | Latine vs White | 0.16 | 0.08–0.32 | <.001 |
|  | Asian vs White | 1.41 | 0.86–2.30 | 0.171 |
|  | Young Adult vs Adolescent | 0.90 | 0.56–1.42 | 0.639 |
|  | Adult vs Adolescent | 0.53 | 0.33–0.86 | 0.011 |
|  | Low SES vs High SES | 0.89 | 0.60–1.31 | 0.552 |
|  | Limited vs Fluent English | 0.89 | 0.60–1.31 | 0.552 |
|  | Non-Urban vs Urban | 1.00 | 0.68–1.48 | 1.000 |
| Mistral Advanced | Female vs Male | 1.22 | 0.79–1.89 | 0.373 |
|  | Black vs White | 0.18 | 0.09–0.37 | <.001 |
|  | Latine vs White | 0.41 | 0.23–0.74 | 0.003 |
|  | Asian vs White | 0.66 | 0.39–1.14 | 0.135 |
|  | Young Adult vs Adolescent | 0.59 | 0.35–1.02 | 0.059 |
|  | Adult vs Adolescent | 0.76 | 0.45–1.27 | 0.293 |
|  | Low SES vs High SES | 0.82 | 0.53–1.27 | 0.373 |
|  | Limited vs Fluent English | 1.05 | 0.68–1.63 | 0.824 |
|  | Non-Urban vs Urban | 1.05 | 0.68–1.63 | 0.824 |
| Claude Base | Female vs Male | 0.63 | 0.45–0.90 | 0.010 |
|  | Black vs White | 0.51 | 0.32–0.82 | 0.006 |
|  | Latine vs White | 0.33 | 0.20–0.55 | <.001 |
|  | Asian vs White | 1.93 | 1.19–3.14 | 0.008 |
|  | Young Adult vs Adolescent | 0.91 | 0.59–1.39 | 0.664 |
|  | Adult vs Adolescent | 0.65 | 0.42–1.00 | 0.050 |
|  | Low SES vs High SES | 0.69 | 0.49–0.99 | 0.041 |
|  | Limited vs Fluent English | 0.92 | 0.65–1.31 | 0.656 |
|  | Non-Urban vs Urban | 0.98 | 0.69–1.40 | 0.929 |
| Claude Advanced | Female vs Male | 0.04 | 0.01–0.09 | <.001 |
|  | Black vs White | 0.00 | 0.00–Inf | 0.988 |
|  | Latine vs White | 0.00 | 0.00–Inf | 0.988 |
|  | Asian vs White | 4.34 | 2.18–8.66 | <.001 |
|  | Young Adult vs Adolescent | 2.96 | 1.30–6.75 | 0.010 |
|  | Adult vs Adolescent | 1.56 | 0.68–3.56 | 0.296 |
|  | Low SES vs High SES | 0.35 | 0.18–0.69 | 0.002 |
|  | Limited vs Fluent English | 0.76 | 0.39–1.46 | 0.403 |
|  | Non-Urban vs Urban | 1.06 | 0.55–2.04 | 0.867 |

***Note:*** *Reference categories: Male (gender), White (race/ethnicity), Adolescent (age group), High SES (socioeconomic status), Fluent English (language), Urban (urbanicity). — indicates non-estimable value due to quasi-complete separation. OR=odds ratio; CI=confidence interval.*

**Table S2.** Per-Model Logistic Regression Odds Ratios for Demographic Predictors of Major Depressive Disorder Diagnosis (Ambiguous Vignettes).

| **LLM** | **Predictor** | **OR** | **95% CI** | **P** |
| --- | --- | --- | --- | --- |
| ChatGPT Base | Female vs Male | 0.25 | 0.17–0.38 | <.001 |
|  | Black vs White | 11.23 | 6.24–20.23 | <.001 |
|  | Latine vs White | 6.73 | 3.88–11.68 | <.001 |
|  | Asian vs White | 1.04 | 0.61–1.75 | 0.893 |
|  | Young Adult vs Adolescent | 1.69 | 1.05–2.72 | 0.031 |
|  | Adult vs Adolescent | 1.74 | 1.08–2.80 | 0.023 |
|  | Low SES vs High SES | 1.97 | 1.33–2.92 | <.001 |
|  | Limited vs Fluent English | 1.45 | 0.98–2.14 | 0.062 |
|  | Non-Urban vs Urban | 0.81 | 0.55–1.19 | 0.277 |
| ChatGPT Advanced | Female vs Male | 0.84 | 0.54–1.30 | 0.430 |
|  | Black vs White | 7.64 | 4.42–13.21 | <.001 |
|  | Latine vs White | 15.95 | 8.87–28.68 | <.001 |
|  | Asian vs White | 0.00 | 0.00–Inf | 0.974 |
|  | Young Adult vs Adolescent | 0.65 | 0.38–1.13 | 0.127 |
|  | Adult vs Adolescent | 0.43 | 0.25–0.75 | 0.003 |
|  | Low SES vs High SES | 1.32 | 0.85–2.06 | 0.216 |
|  | Limited vs Fluent English | 1.26 | 0.81–1.96 | 0.311 |
|  | Non-Urban vs Urban | 0.84 | 0.54–1.30 | 0.430 |
| DeepSeek Base | Female vs Male | 1.10 | 0.72–1.69 | 0.662 |
|  | Black vs White | 6.07 | 3.50–10.53 | <.001 |
|  | Latine vs White | 12.37 | 6.97–21.94 | <.001 |
|  | Asian vs White | 0.00 | 0.00–Inf | 0.975 |
|  | Young Adult vs Adolescent | 0.65 | 0.39–1.10 | 0.110 |
|  | Adult vs Adolescent | 0.51 | 0.30–0.86 | 0.012 |
|  | Low SES vs High SES | 1.53 | 1.00–2.36 | 0.051 |
|  | Limited vs Fluent English | 0.95 | 0.62–1.46 | 0.827 |
|  | Non-Urban vs Urban | 1.05 | 0.68–1.61 | 0.827 |
| DeepSeek Advanced | Female vs Male | 0.75 | 0.49–1.14 | 0.177 |
|  | Black vs White | 7.13 | 4.18–12.17 | <.001 |
|  | Latine vs White | 13.33 | 7.51–23.66 | <.001 |
|  | Asian vs White | 0.29 | 0.14–0.59 | <.001 |
|  | Young Adult vs Adolescent | 0.62 | 0.37–1.04 | 0.071 |
|  | Adult vs Adolescent | 0.49 | 0.29–0.83 | 0.007 |
|  | Low SES vs High SES | 1.47 | 0.96–2.23 | 0.073 |
|  | Limited vs Fluent English | 1.27 | 0.84–1.93 | 0.261 |
|  | Non-Urban vs Urban | 0.86 | 0.56–1.30 | 0.464 |
| Llama Base | Female vs Male | 0.11 | 0.07–0.17 | <.001 |
|  | Black vs White | 2.69 | 1.55–4.67 | <.001 |
|  | Latine vs White | 6.29 | 3.44–11.47 | <.001 |
|  | Asian vs White | 0.86 | 0.51–1.47 | 0.586 |
|  | Young Adult vs Adolescent | 1.10 | 0.67–1.78 | 0.710 |
|  | Adult vs Adolescent | 0.94 | 0.58–1.53 | 0.805 |
|  | Low SES vs High SES | 1.15 | 0.78–1.72 | 0.480 |
|  | Limited vs Fluent English | 1.30 | 0.88–1.94 | 0.190 |
|  | Non-Urban vs Urban | 0.90 | 0.61–1.34 | 0.614 |
| Llama Advanced | Female vs Male | 2.25 | 1.43–3.52 | <.001 |
|  | Black vs White | 10.82 | 5.42–21.57 | <.001 |
|  | Latine vs White | 8.52 | 4.27–17.01 | <.001 |
|  | Asian vs White | 0.00 | 0.00–Inf | 0.976 |
|  | Young Adult vs Adolescent | 1.08 | 0.62–1.88 | 0.778 |
|  | Adult vs Adolescent | 1.76 | 1.02–3.04 | 0.041 |
|  | Low SES vs High SES | 1.00 | 0.64–1.56 | 1.000 |
|  | Limited vs Fluent English | 1.23 | 0.79–1.92 | 0.365 |
|  | Non-Urban vs Urban | 0.86 | 0.55–1.34 | 0.497 |
| Mistral Base | Female vs Male | 0.34 | 0.23–0.49 | <.001 |
|  | Black vs White | 5.00 | 2.97–8.40 | <.001 |
|  | Latine vs White | 8.16 | 4.68–14.24 | <.001 |
|  | Asian vs White | 1.25 | 0.76–2.04 | 0.381 |
|  | Young Adult vs Adolescent | 1.00 | 0.64–1.57 | 1.000 |
|  | Adult vs Adolescent | 1.08 | 0.69–1.70 | 0.729 |
|  | Low SES vs High SES | 1.22 | 0.84–1.76 | 0.300 |
|  | Limited vs Fluent English | 1.17 | 0.81–1.70 | 0.397 |
|  | Non-Urban vs Urban | 0.98 | 0.68–1.42 | 0.925 |
| Mistral Advanced | Female vs Male | 0.36 | 0.24–0.54 | <.001 |
|  | Black vs White | 11.72 | 6.54–20.99 | <.001 |
|  | Latine vs White | 8.79 | 4.98–15.52 | <.001 |
|  | Asian vs White | 0.87 | 0.48–1.58 | 0.647 |
|  | Young Adult vs Adolescent | 1.63 | 1.00–2.66 | 0.049 |
|  | Adult vs Adolescent | 1.68 | 1.03–2.74 | 0.037 |
|  | Low SES vs High SES | 2.79 | 1.85–4.18 | <.001 |
|  | Limited vs Fluent English | 1.36 | 0.91–2.02 | 0.132 |
|  | Non-Urban vs Urban | 0.98 | 0.66–1.46 | 0.920 |
| Claude Base | Female vs Male | 0.35 | 0.23–0.55 | <.001 |
|  | Black vs White | 3.33 | 1.86–5.96 | <.001 |
|  | Latine vs White | 9.67 | 5.37–17.39 | <.001 |
|  | Asian vs White | 0.00 | 0.00–Inf | 0.975 |
|  | Young Adult vs Adolescent | 1.04 | 0.61–1.77 | 0.892 |
|  | Adult vs Adolescent | 1.16 | 0.68–1.97 | 0.587 |
|  | Low SES vs High SES | 1.31 | 0.85–2.03 | 0.224 |
|  | Limited vs Fluent English | 1.19 | 0.77–1.84 | 0.438 |
|  | Non-Urban vs Urban | 0.98 | 0.63–1.51 | 0.912 |
| Claude Advanced | Female vs Male | 0.88 | 0.57–1.37 | 0.573 |
|  | Black vs White | 5.55 | 3.27–9.42 | <.001 |
|  | Latine vs White | 15.95 | 8.83–28.82 | <.001 |
|  | Asian vs White | 0.00 | 0.00–Inf | 0.974 |
|  | Young Adult vs Adolescent | 0.44 | 0.26–0.77 | 0.004 |
|  | Adult vs Adolescent | 0.34 | 0.20–0.60 | <.001 |
|  | Low SES vs High SES | 1.14 | 0.73–1.77 | 0.573 |
|  | Limited vs Fluent English | 1.26 | 0.81–1.96 | 0.311 |
|  | Non-Urban vs Urban | 0.93 | 0.60–1.44 | 0.735 |

***Note:*** *Reference categories: Male (gender), White (race/ethnicity), Adolescent (age group), High SES (socioeconomic status), Fluent English (language), Urban (urbanicity). — indicates non-estimable value due to quasi-complete separation. OR=odds ratio; CI=confidence interval.*

**Table S3.** Cochran’s Rule Compliance for Chi-Square Tests by LLM and Demographic Variable.

| **LLM** | **Demographic** | **N Cells** | **Min Exp.** | **N < 5** | **% < 5** | **N < 1** | **Rule Met** |
| --- | --- | --- | --- | --- | --- | --- | --- |
| ChatGPT Base | gender | 8 | 2.00 | 2 | 25% | 0 | No |
|  | race/ethnicity | 16 | 1.00 | 4 | 25% | 0 | No |
|  | age/group | 12 | 1.33 | 3 | 25% | 0 | No |
|  | ses | 8 | 2.00 | 2 | 25% | 0 | No |
|  | language | 8 | 2.00 | 2 | 25% | 0 | No |
|  | urbanicity | 8 | 2.00 | 2 | 25% | 0 | No |
| ChatGPT Advanced | gender | 8 | 4.00 | 2 | 25% | 0 | No |
|  | race/ethnicity | 16 | 2.00 | 4 | 25% | 0 | No |
|  | age/group | 12 | 2.67 | 3 | 25% | 0 | No |
|  | ses | 8 | 4.00 | 2 | 25% | 0 | No |
|  | language | 8 | 4.00 | 2 | 25% | 0 | No |
|  | urbanicity | 8 | 4.00 | 2 | 25% | 0 | No |
| DeepSeek Base | gender | 8 | 10.00 | 0 | 0% | 0 | Yes |
|  | race/ethnicity | 16 | 5.00 | 0 | 0% | 0 | Yes |
|  | age/group | 12 | 6.67 | 0 | 0% | 0 | Yes |
|  | ses | 8 | 10.00 | 0 | 0% | 0 | Yes |
|  | language | 8 | 10.00 | 0 | 0% | 0 | Yes |
|  | urbanicity | 8 | 10.00 | 0 | 0% | 0 | Yes |
| DeepSeek Advanced | gender | 8 | 6.00 | 0 | 0% | 0 | Yes |
|  | race/ethnicity | 16 | 3.00 | 4 | 25% | 0 | No |
|  | age/group | 12 | 4.00 | 3 | 25% | 0 | No |
|  | ses | 8 | 6.00 | 0 | 0% | 0 | Yes |
|  | language | 8 | 6.00 | 0 | 0% | 0 | Yes |
|  | urbanicity | 8 | 6.00 | 0 | 0% | 0 | Yes |
| Llama Base | gender | 8 | 1.00 | 2 | 25% | 0 | No |
|  | race/ethnicity | 16 | 0.50 | 4 | 25% | 4 | No |
|  | age/group | 12 | 0.67 | 3 | 25% | 3 | No |
|  | ses | 8 | 1.00 | 2 | 25% | 0 | No |
|  | language | 8 | 1.00 | 2 | 25% | 0 | No |
|  | urbanicity | 8 | 1.00 | 2 | 25% | 0 | No |
| Llama Advanced | gender | 8 | 26.00 | 0 | 0% | 0 | Yes |
|  | race/ethnicity | 16 | 13.00 | 0 | 0% | 0 | Yes |
|  | age/group | 12 | 17.33 | 0 | 0% | 0 | Yes |
|  | ses | 8 | 26.00 | 0 | 0% | 0 | Yes |
|  | language | 8 | 26.00 | 0 | 0% | 0 | Yes |
|  | urbanicity | 8 | 26.00 | 0 | 0% | 0 | Yes |
| Mistral Base | gender | 8 | 20.50 | 0 | 0% | 0 | Yes |
|  | race/ethnicity | 16 | 10.25 | 0 | 0% | 0 | Yes |
|  | age/group | 12 | 13.67 | 0 | 0% | 0 | Yes |
|  | ses | 8 | 20.50 | 0 | 0% | 0 | Yes |
|  | language | 8 | 20.50 | 0 | 0% | 0 | Yes |
|  | urbanicity | 8 | 20.50 | 0 | 0% | 0 | Yes |
| Mistral Advanced | gender | 8 | 49.50 | 0 | 0% | 0 | Yes |
|  | race/ethnicity | 16 | 24.75 | 0 | 0% | 0 | Yes |
|  | age/group | 12 | 33.00 | 0 | 0% | 0 | Yes |
|  | ses | 8 | 49.50 | 0 | 0% | 0 | Yes |
|  | language | 8 | 49.50 | 0 | 0% | 0 | Yes |
|  | urbanicity | 8 | 49.50 | 0 | 0% | 0 | Yes |
| Claude Base | gender | 8 | 14.50 | 0 | 0% | 0 | Yes |
|  | race/ethnicity | 16 | 7.25 | 0 | 0% | 0 | Yes |
|  | age/group | 12 | 9.67 | 0 | 0% | 0 | Yes |
|  | ses | 8 | 14.50 | 0 | 0% | 0 | Yes |
|  | language | 8 | 14.50 | 0 | 0% | 0 | Yes |
|  | urbanicity | 8 | 14.50 | 0 | 0% | 0 | Yes |
| Claude Advanced | gender | 8 | 37.50 | 0 | 0% | 0 | Yes |
|  | race/ethnicity | 16 | 18.75 | 0 | 0% | 0 | Yes |
|  | age/group | 12 | 25.00 | 0 | 0% | 0 | Yes |
|  | ses | 8 | 37.50 | 0 | 0% | 0 | Yes |
|  | language | 8 | 37.50 | 0 | 0% | 0 | Yes |
|  | urbanicity | 8 | 37.50 | 0 | 0% | 0 | Yes |

***Note:*** *Cochran’s rule requires no expected frequency < 1 and no more than 20% of cells with expected frequency < 5.*

**Table S4.** Pairwise McNemar Post-Hoc Comparisons of Diagnosis Rates Across LLM Configurations (Ambiguous Vignettes).

| **Comparison** | **χ²** | **P** | **P (adjusted)** | **Significant** |
| --- | --- | --- | --- | --- |
| chatgpt_base vs chatgpt_advanced | 16.80 | <.001 | <.001 | Yes |
| chatgpt_base vs deepseek_base | 13.83 | <.001 | <.001 | Yes |
| chatgpt_base vs deepseek_advanced | 15.01 | <.001 | <.001 | Yes |
| chatgpt_base vs llama_base | 0.00 | 1.000 | 1.000 | No |
| chatgpt_base vs llama_advanced | 48.94 | <.001 | <.001 | Yes |
| chatgpt_base vs mistral_base | 0.65 | 0.420 | 0.472 | No |
| chatgpt_base vs mistral_advanced | 11.24 | <.001 | 0.001 | Yes |
| chatgpt_base vs claude_base | 76.90 | <.001 | <.001 | Yes |
| chatgpt_base vs claude_advanced | 55.71 | <.001 | <.001 | Yes |
| chatgpt_advanced vs deepseek_base | 77.29 | <.001 | <.001 | Yes |
| chatgpt_advanced vs deepseek_advanced | 0.00 | 1.000 | 1.000 | No |
| chatgpt_advanced vs llama_base | 7.77 | 0.005 | 0.007 | Yes |
| chatgpt_advanced vs llama_advanced | 6.78 | 0.009 | 0.012 | Yes |
| chatgpt_advanced vs mistral_base | 13.95 | <.001 | <.001 | Yes |
| chatgpt_advanced vs mistral_advanced | 0.00 | 1.000 | 1.000 | No |
| chatgpt_advanced vs claude_base | 156.01 | <.001 | <.001 | Yes |
| chatgpt_advanced vs claude_advanced | 13.02 | <.001 | <.001 | Yes |
| deepseek_base vs deepseek_advanced | 80.01 | <.001 | <.001 | Yes |
| deepseek_base vs llama_base | 7.76 | 0.005 | 0.007 | Yes |
| deepseek_base vs llama_advanced | 91.35 | <.001 | <.001 | Yes |
| deepseek_base vs mistral_base | 4.19 | 0.041 | 0.050 | Yes |
| deepseek_base vs mistral_advanced | 32.17 | <.001 | <.001 | Yes |
| deepseek_base vs claude_base | 45.26 | <.001 | <.001 | Yes |
| deepseek_base vs claude_advanced | 107.01 | <.001 | <.001 | Yes |
| deepseek_advanced vs llama_base | 7.12 | 0.008 | 0.010 | Yes |
| deepseek_advanced vs llama_advanced | 6.19 | 0.013 | 0.016 | Yes |
| deepseek_advanced vs mistral_base | 12.77 | <.001 | <.001 | Yes |
| deepseek_advanced vs mistral_advanced | 0.00 | 1.000 | 1.000 | No |
| deepseek_advanced vs claude_base | 155.01 | <.001 | <.001 | Yes |
| deepseek_advanced vs claude_advanced | 15.72 | <.001 | <.001 | Yes |
| llama_base vs llama_advanced | 25.78 | <.001 | <.001 | Yes |
| llama_base vs mistral_base | 0.83 | 0.363 | 0.418 | No |
| llama_base vs mistral_advanced | 10.96 | <.001 | 0.001 | Yes |
| llama_base vs claude_base | 53.93 | <.001 | <.001 | Yes |
| llama_base vs claude_advanced | 23.71 | <.001 | <.001 | Yes |
| llama_advanced vs mistral_base | 39.25 | <.001 | <.001 | Yes |
| llama_advanced vs mistral_advanced | 3.89 | 0.049 | 0.058 | No |
| llama_advanced vs claude_base | 180.01 | <.001 | <.001 | Yes |
| llama_advanced vs claude_advanced | 0.02 | 0.890 | 0.977 | No |
| mistral_base vs mistral_advanced | 11.53 | <.001 | 0.001 | Yes |
| mistral_base vs claude_base | 35.51 | <.001 | <.001 | Yes |
| mistral_base vs claude_advanced | 35.75 | <.001 | <.001 | Yes |
| mistral_advanced vs claude_base | 143.31 | <.001 | <.001 | Yes |
| mistral_advanced vs claude_advanced | 6.25 | 0.012 | 0.016 | Yes |
| claude_base vs claude_advanced | 182.01 | <.001 | <.001 | Yes |

***Note:*** *P values adjusted using the Benjamini-Hochberg procedure to control the false discovery rate across 45 pairwise comparisons.*
